# Rate Constants and Activation Energies for Gas‐Phase Reactions of Three Cyclic Volatile Methyl Siloxanes with the Hydroxyl Radical

**DOI:** 10.1002/kin.20919

**Published:** 2015-04-23

**Authors:** Andreas Safron, Michael Strandell, Amelie Kierkegaard, Matthew Macleod

**Affiliations:** ^1^ Department of Environmental Science and Analytical Chemistry (ACES)Stockholm UniversitySE‐10691StockholmSweden

## Abstract

Reaction with hydroxyl radicals (OH) is the major pathway for removal of cyclic volatile methyl siloxanes (cVMS) from air. We present new measurements of second‐order rate constants for reactions of the cVMS octamethylcyclotetrasiloxane (D_4_), decamethylcyclopentasiloxane (D_5_), and dodecamethylcyclohexasiloxane (D_6_) with OH determined at temperatures between 313 and 353 K. Our measurements were made using the method of relative rates with cyclohexane as a reference substance and were conducted in a 140‐mL gas‐phase reaction chamber with online mass spectrometry analysis. When extrapolated to 298 K, our measured reaction rate constants of D_4_ and D_5_ with the OH radical are 1.9 × 10^−12^ (95% confidence interval (CI): (1.7–2.2) × 10^−12^) and 2.6 × 10^−12^ (CI: (2.3–2.9) × 10^−12^) cm^3^ molecule^−1^ s^−1^, respectively, which are 1.9× and 1.7× faster than previous measurements. Our measured rate constant for D_6_ is 2.8 × 10^−12^ (CI: (2.5–3.2) × 10^−12^) cm^3^ molecule^−1^ s^−1^ and to our knowledge there are no comparable laboratory measurements in the literature. Reaction rates for D_5_ were 33% higher than for D_4_ (CI: 30–37%), whereas the rates for D_6_ were only 8% higher than for D_5_ (CI: 5–10%). The activation energies of the reactions of D_4_, D_5_, and D_6_ with OH were not statistically different and had a value of 4300 ± 2800 J/mol.

## INTRODUCTION

**Figure 1:**
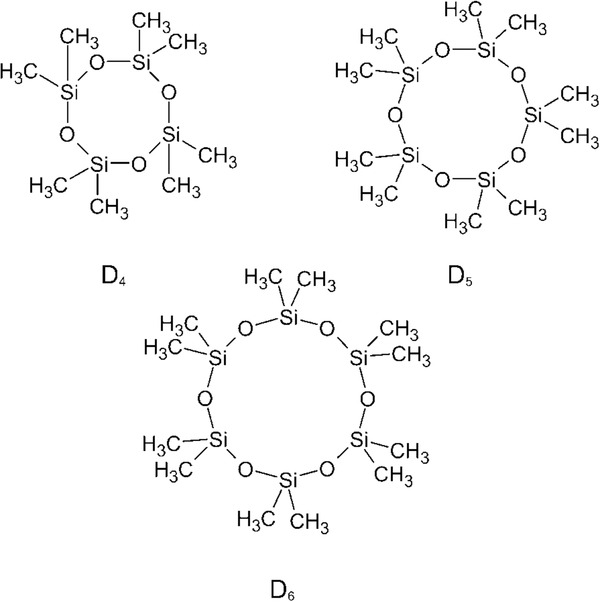
Chemical structures of D_4_, D_5_, and D_6._

Cyclic volatile methyl siloxanes (cVMS) are high production volume chemicals used extensively in personal care products and cosmetics as neutral carriers and to improve the spreading of the product and provide a silky feel 1, 2, 3, 4. The cVMS are composed of several (CH_3_)_2_–Si–O– units forming a ring with a Si–O– backbone (Fig. 1). Their abbreviated nomenclature is based on General Electric's siloxane notation 5. Using information provided by an industry group, the U.K. Environment Agency estimated that the usage of cVMS in personal care products within the European Union in 2004 amounted to 579 tons of octamethyltetrasiloxane (D_4_), 17,300 tons of decamethylpentasiloxane (D_5_), and 1989 tons of dodecamethylhexasiloxane (D_6_) 6, 7, 8. The estimated amounts of cVMS used in other applications, such as household products, industrial dry cleaning, and as a chemical intermediate, remain confidential 6, 7, 8.

Measurements in air at remote locations and global fate modeling studies have shown that cVMS are globally distributed 9, 10, 11, 12, 13, 14, 15, 16. Atmospheric modeling has indicated that reaction with the OH radical is the dominant process that removes D_5_ from the atmosphere 15. The degradation products of cVMS are considerably less volatile than the parent compounds and have recently been suggested to contribute to fine particle formation in the atmosphere 17. Currently, only one set of laboratory measurements of the reaction rates of hexamethyltrisiloxane (D_3_), D_4_, and D_5_ with the OH radical in the gas phase at 298 K has been reported 18. No experimental value has been published for the reaction rate of D_6_ with OH, but it was estimated in one study based on spatial variability in monitoring data and a Junge relationship 19. These available data suggest atmospheric lifetimes of 22 days for D_3_, 11 days for D_4_, 7 days for D_5_, and 6 days for D_6_ when assuming a temperature of 298 K, and a 24‐h average concentration of OH radicals of 10^6^ molecules cm^–3^. The temperature dependence of the reaction rates of cVMS with the OH radical has up to now not been determined.

Here we report new measurements of the temperature dependence of the reaction of D_4_, D_5_, and D_6_ with the OH radical. To our knowledge, these are the first reported experimental data on reaction rates of D_6_ with the OH radical. We measured the reaction rates in a gas‐phase reaction chamber at temperatures between 313 and 353 K using the method of relative rates with cyclohexane as a reference substance. Our measurements follow the approach pioneered by Anderson and Hites 20 that has been applied to a range of semivolatile compounds 21, 22, 23, 24, 25, 26, 27, 28, 29, 30, 31, 32, 33, 34. We use the Arrhenius equation to calculate activation energies of the reactions and to extrapolate the reaction rates to ambient temperatures.

## MATERIALS AND METHODS

### Reaction Chamber

**Figure 2:**
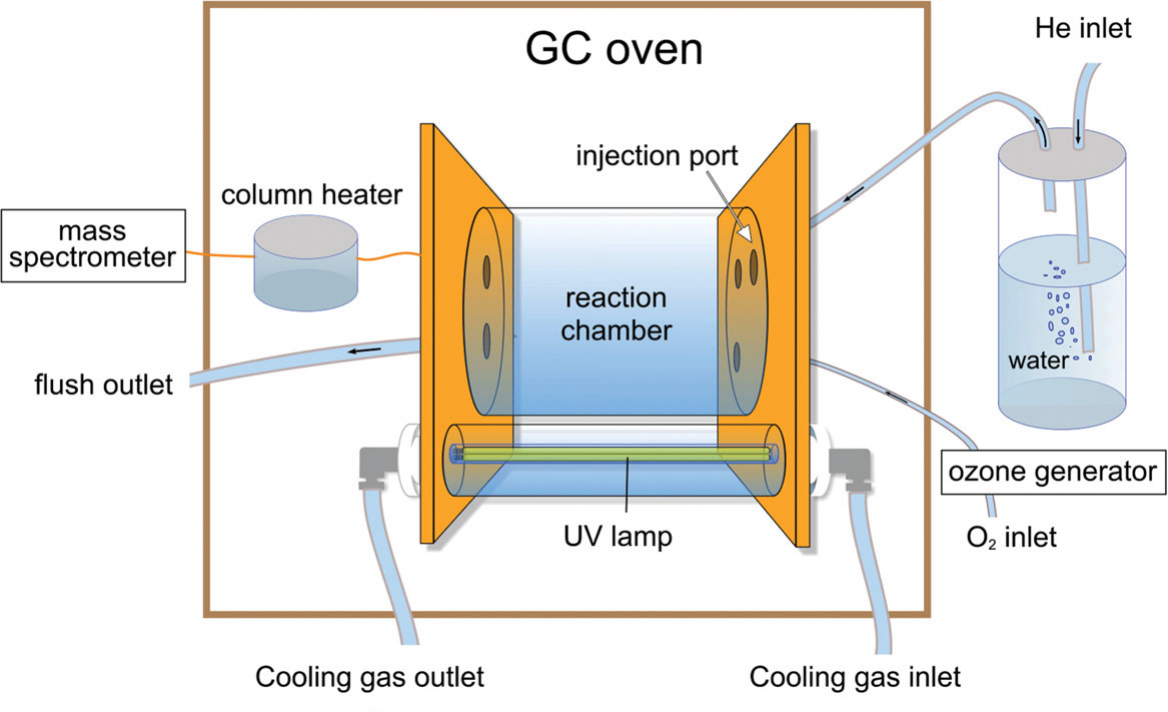
Schematic illustration of the 140 mL gas‐phase reaction chamber with online mass spectrometry analysis.

We constructed a small‐scale gas‐phase reaction chamber with chemical analysis by online mass spectrometry (Fig. 2), that is similar to the system described by Hites and co‐workers in several publications 20, 34. The chamber is a quartz glass cylinder (4.2 cm i.d. × 10 cm length, 140 mL in volume) capped by two gold‐coated stainless steel plates. There are three inlet ports on the influent side for the introduction of ozone, water‐saturated helium, and the tested substances via either a split/splitless injector or direct injection into the chamber. Only the direct injection port was used in the experiments described in this article. Ozone is generated by a corona discharge ozone generator (Type C300; Sander GmbH, Wuppertal Germany) and is introduced into the chamber through a Teflon tube (0.6 mm i.d.). Water‐saturated helium is produced by bubbling helium gas through MilliQ water in a gas washing bottle. There are two ports on the effluent side, one connecting the chamber to a Trace DSQ mass spectrometer (Thermo Finnigan, Stockholm Sweden) and one for flushing the chamber between experiments. The chamber is connected to the mass spectrometer by a deactivated fused silica capillary (0.1 mm i.d. × 5 m length) that is heated to 523 K (250°C) in a cylindrical metal block. With the exception of the capillary columns connecting the chamber to the mass spectrometer and to the split‐splitless injector, all connections to the reaction chamber can be closed by toggle valves to avoid gas leakage during experiments. A pen‐shaped UV lamp (Ultra‐Violet Products Ltd, Cambridge UK) is mounted inside a quartz glass cylinder at a distance of 2 cm from the reaction chamber. Because the lifetime of the UV lamp is reduced by operating temperatures above 313 K, it was cooled by a constant flow of room temperature air through the quartz‐glass cylinder surrounding it. The reaction chamber and the UV lamp are mounted inside a gas chromatography oven that holds the entire apparatus at a constant temperature during experiments. The mass spectrometer was operated with electron impact ionization in either selective ion monitoring mode or full‐scan mode.

### Reagents

Cyclohexane (Merck, Darmstadt Germany; 99.5%), dichloromethane (Merck; purity 99.8%), D_4_ (Fluka, Stockholm Sweden; purity 99.0%), D_5_ (Fluka; purity 97.0%), and D_6_ (Fluorochem, Derbyshire UK; purity not stated) were used in our experiments. Oxygen was of 99.6% purity, helium was of 99.999% purity. Deionized, filtered water with a resistivity *R* = 18.2 MΩ cm was taken from a Millipore system.

### Experimental Procedure

In a typical experiment, the reaction chamber was first flushed for 10 min with a flow of 44 mL min^−1^ He gas and 6.8 mL min^−1^ O_2_‐O_3_ gas mixture. Next, the gas flows were stopped, but the He inlet port was left open to maintain the pressure in the reactor close to 1 atm. The mass spectrometer was started, recording the signals of ions *m/z* 56 for the reference compound cyclohexane, and *m/z* 281 for D_4_, *m/z* 355 for D_5_, and *m/z* 429 for D_6_, which are the [M – CH_3_]^+^ fragment ions. These fragment ions were selected because they have stronger signals than the parent ions and were not subject to interferences from other components of the mixture or their degradation products, as determined by full‐scan analysis of each component individually, and several preexperiments using only one of the cVMS compounds at a time. The signal intensity of each ion (*i_X_*) was interpreted as a measure of the gas‐phase concentration of the relevant species. After recording the background signal at these *m/z* for 1 min, 1 μL of a solution containing mole fractions of 3.3% D_4_, 2.3% D_5_, 2.0% D_6_, 20.6% cyclohexane, and 71.8% dichloromethane was injected. The mass spectrometer signals typically reached a stable plateau for all injected compounds after 5 min, sometimes after 8 min for D_6_. When stable signal intensities were observed for a period of at least 5 min, the UV lamp was turned on to initiate the production of OH radicals 21. Reactions were allowed to proceed for an average duration of 3 min. Data collection on the mass spectrometer was then ended, and the chamber was flushed with the He–O_2_–O_3_ mixture for a period of at least 10 min to prepare for the next experiment.

Cyclohexane was selected as the reference compound because it has similar reactivity with the OH radical as the cVMS. It also meets several additional selection criteria for a reference compound, such as availability of a temperature‐dependent rate constant, stability when exposed to UV radiation, and no interference of its fragment ions with the fragment ions of the cVMS or degradation products thereof 33. The GC oven was operated at a temperature between 313 and 353 K in all experiments reported here.

### Calculation of Reaction Rates

The temperature‐dependent rate constant for OH radical reactions with cyclohexane (*k*_cyclohexane_) between 290 and 500 K is 35

(1) k cyclohexane (T)=3.26×10−17T2e−262±33T cm 3 molecule −1s−1

We use *k*_cyclohexane_ calculated with Eq. (1) and the ratios (*k*_cVMS_/*k*_cyclohexane_) calculated with the two methods described below to estimate *k*_cVMS_ from the signal intensities observed during our experiments.

When the same concentration of OH radicals is available to the cVMS and cyclohexane, their concentrations in the reaction chamber at time *t* during an experiment are given by 36

(2) ln[ cVMS ]0[ cVMS ]t=k cVMS k cyclohexane ln[ cyclohexane ]0[ cyclohexane ]t

where [cVMS]_0_, [cVMS]*_t_*, [cyclohexane]_0_, and [cyclohexane]*_t_* denote concentrations of the cVMS and cyclohexane at the beginning of the reaction period and at time *t* during the reaction. *k*_cVMS_ and *k*_cyclohexane_ denote the second‐order reaction rates of cyclohexane and the cVMS with the OH radical. It follows from Eq. (2) that a plot of ln(*i*_cVMS_,_0_/*i*_cVMS_,*_t_*) versus ln(*i*_cyclohexane,0_/*i*_cyclohexane,*t*_) constructed using signal intensities collected at time *t* corresponding to each individual scan cycle of the mass spectrometer is linear with a slope of *k*_cVMS_/*k*_cyclohexane_. This calculation method has the advantage that it is valid if the OH concentration is not constant during the experiment and that covariant measurement errors of the cVMS and cyclohexane signal intensities will cancel out 37. Constructing plots according to Eq. (2) is the most common method of calculating second‐order rate constants measured in relative rate studies 18, 20, 33, 38.

A potential drawback of calculations based on Eq. (2) is that uncertainty and variability in the ratio (*i*_cyclohexane,0_/*i*_cyclohexane,*t*_) is not propagated through linear regression, and the ratio (*k*_cVMS_/*k*_cyclohexane_) will therefore be biased to some degree by regression dilution. Regression dilution occurs if the independent variable in a regression is subject to uncertainty and causes a bias in the calculated slope of the regression toward zero 39.

Here, we explore an alternative method of calculating second‐order rate constants for the cVMS from our experimental data that avoids potential bias from regression dilution. If the concentration of OH is constant over the course of an experiment, the observed signal intensities *i*_cyclohexane_ and *i*_cVMS_ will decline exponentially. In that case, the time series of ln‐transformed signal intensities for cyclohexane and the cVMS can be fitted with separate linear regression equations:

(3) ln(i cyclohexane )=s cyclohexane ×t+c cyclohexane

(4) ln(i cVMS )=s cVMS ×t+c cVMS

*s*_cyclohexane_ and *s*_cVMS_ are the slopes of ln(*i*_cyclohexane_) and ln(*i*_cVMS_) plotted against time, and *c*_cyclohexane_ and *c*_cVMS_ are the intercept values of the linear regressions. The ratio of the two slopes determined in each individual experiment can be used to derive *k*_cVMS_ from the known *k*_cyclohexane_:

(5) k cVMS k cyclohexane =s cVMS s cyclohexane

In contrast to regressions based on Eq. (2), which implicitly assume that there is no measurement error attached to ln(*i*_cyclohexane,0_/*i*_cyclohexane,*t*_), Eq. (5) has the advantage that it allows for propagation of uncertainty in *s*_cyclohexane_. Since linear regressions based on Eqs. (3) and (4) are carried out with the well‐characterized *t* as an independent variable, estimates of *k*_cVMS_ calculated with Eq. (5) should suffer from less bias introduced by regression dilution compared to the method based on Eq. (2). Here we apply and compare both methods to determine *k*_cVMS_.

We estimated the activation energy *E_a_* of the reactions and extrapolated to other temperatures by making relative rate measurements at a range of temperatures between 313 and 353 K and applying the Arrhenius equation:

(6) $$\begin{array}{c} k\left(T\right)=A\times e^{-\frac{E_{a}}{RT}} \end{array}$$

where *A* is a substance‐specific preexponential factor and *R* is the universal gas constant.

In most experiments, the mass spectrometer was operated in selective ion monitoring mode, but some experiments were conducted in full‐scan mode with injections of only D_6_ in dichloromethane, but otherwise maintaining the same experimental protocol. The full‐scan experiments were used to identify possible degradation products of D_6_ in the reactor.

## RESULTS

**Figure 3:**
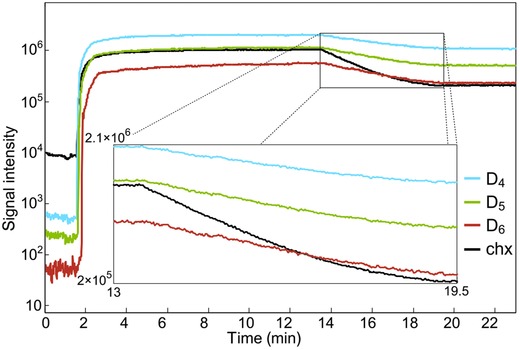
Ion traces for cyclohexane (chx) and the cVMS (D_4_, D_5_, and D_6_) from a typical experiment. The sample was injected after 1 min. Reactions of cyclohexane and the cVMS are evident during the period between minute 13 and minute 19.5 and are shown enlarged in the figure inset.

An ion trace from a typical experiment is shown in Fig. 3 on a logarithmic intensity axis. The sample was injected after 1 min, and the ion intensities increase shortly thereafter, followed by a stabilization period. On the basis of measurements using KI iodometry 40, we estimate that concentrations of ozone in the reactor were (1.4–2.5) × 10^16^ molecules cm^–3^, which is very similar to the concentrations reported by Brubaker and Hites 22, 23, 24. In the experiment shown in Fig. 3, stable concentrations of all substances are observed from minute 5 to minute 13, indicating minimal losses through a reaction with ozone, leaks or by adsorption to the chamber walls. After 13 min of signal recording, the UV lamp was turned on, producing OH radicals that resulted in the decay of the substances. The decay is exponential for a period beginning shortly after the lamp is turned on, then the slopes begin to flatten, possibly as a result of depletion of ozone in the reactor. Only the period with exponential decay of the analytes was selected to determine the reaction rate constants of the cVMS by both calculation methods and also to estimate the steady‐state concentration of OH radicals.

**Figure 4:**
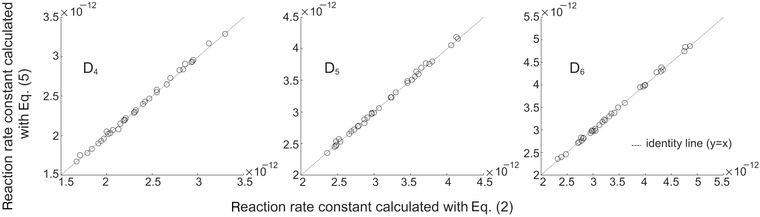
Comparison between *k*_cVMS_ calculated with the two alternative methods. The dotted line is the identity line (*y* = *x*), and units on both axes are cm^3^molecule^−1^ s^−1^.

The reaction rates of D_4_, D_5_, and D_6_ in each individual experiment calculated with a linear regression based on Eq. (2) and with the method involving Eqs. (3)–(5) are reported in the Supporting Information. The coefficient of determination (*R* ^2^) for the linear regressions based on Eq. (2) ranged from 0.772 to 0.999. The regressions for cyclohexane based on Eq. (3) had *R* ^2^ values ranging from 0.969 to 0.999, and those for the cVMS based on Eq. (4) had values that ranged from 0.852 to 0.998 for D_4_, 0.862 to 0.997 for D_5_, and 0.746 to 0.995 for D_6_. On the basis of the observed rate of decay of cyclohexane in a regression based on Eq. (3) and its recommended rate constant (Eq. (1)), we estimated that the OH radical concentration in our reactor ranged from 1.42 × 10^8^ to 2.16 × 10^9^ molecules cm^−3^ in the 35 individual experiments that we conducted. The reaction rates of cVMS with OH calculated with the two methods differ only marginally, and there is no evidence of a significant bias in the method based on Eq. (2) (Fig. 4).

**Figure 5:**
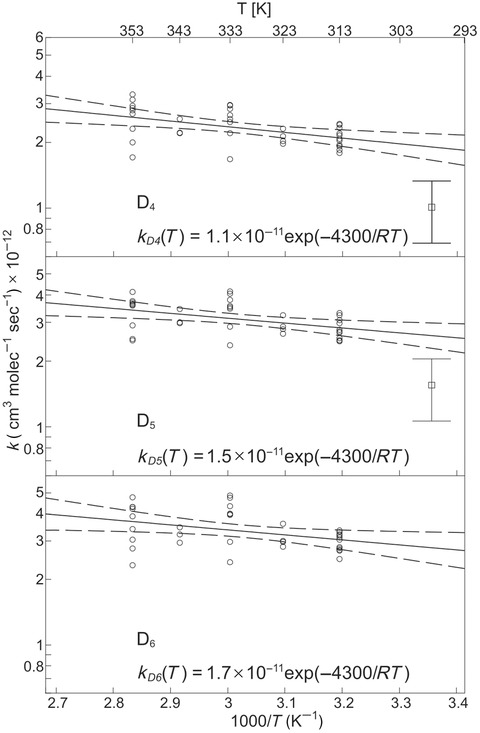
Arrhenius plots of measured reaction rate constants of D_4_, D_5_, and D_6_ with OH calculated from experimental data with Eq. (5). The solid lines show the log‐linear regressions from a linear mixed model used to evaluate the activation energies, and the dotted lines show the 95% confidence intervals for the slope of the regression. Reaction rates of D_4_ and D_5_ from smog chamber studies 18 are shown as squares together with their corresponding 95% CI.

When we constructed Arrhenius plots using rate constants calculated with Eq. (5) and applied a normal linear regression model, we observed a clear overlap of the confidence intervals of the slopes for D_4_, D_5_, and D_6_ (see the Supporting Information). A test of the null hypothesis that the slopes of the three plots were not different gave a *p* value of 0.51, indicating that the activation energies (*E_a_*) for the three cVMS are not significantly different. Furthermore, we observed that the residuals for the three cVMS measured in each experiment were covariate. Therefore, we combined the measurements for D_4_, D_5_, and D_6_ in a linear mixed model that assumes the slope of the Arrhenius plots for all three cVMS is the same, but that the intercepts may differ. The model assumes that random errors occur additively at two levels; in each experiment and for each substance independently. The linear mixed model was implemented using the “mixed” command in Stata 13 (www.stata.com) and is illustrated in Fig. 5.

The common estimate of *E_a_* for all three cVMS from the linear mixed model is 4300 J/mol, with a 95% confidence interval (CI) ranging from 1600 to 7100 J/mol. The preexponential factors, *A* in Eq. (6), are, for D_4_, 1.14 × 10^−11^ cm^3^ molecule^−1^ s^−1^ (95% CI: (0.42–3.09) × 10^−11^); for D_5_, 1.53 × 10^−11^ cm^3^ molecule^−1^ s^−1^ (CI: (0.56–4.17) × 10^−11^); and for D_6_, 1.7 × 10^−11^ cm^3^ molecule^–1^ s^−1^ (CI: (0.56–4.61) × 10^−11^).

The calculated Arrhenius preexponential factors for D_4_, D_5_, and D_6_ are not significantly different from each other. However, because the three cVMS were injected simultaneously and the measured reaction rate constants were covariate, it was possible to discern the difference in their reaction rates in the linear mixed model. The reaction of D_5_ with the OH radical was 33% faster than the reaction of D_4_ (CI: 30–37%). Similarly, the reaction of D_6_ with the OH radical was on average 8% faster than the reaction of D_5_ (CI: 5–10%).

We identified two major ions of a degradation product of D_6_ in full‐scan experiments that included only D_6_ in a dichloromethane solution. When the degradation began, the intensity of the ions *m/z* 399 and *m/z* 415 increased, a signal also seen for the isotopic peaks *m/z* 400 and *m/z* 416. These ions may correspond to [M – 31]^+^ and [M – 47]^+^ fragment ions of a silanol that would be called D_5_TOH according to the abbreviated siloxane nomenclature. A previous study 41 identified the analogous silanol D_4_TOH as a product of the reaction of D_5_ with OH, and major fragments in the spectra of D_4_TOH are the [M – 31]^+^ and [M – 47]^+^ ions. Another study 42 identified D_3_TOH as a degradation product of D_4_. The D_3_TOH fragment ions reported in 42 include [M – 31]^+^ but not [M – 47]^+^.

## DISCUSSION

A major challenge during the development of the reactor system was the condition of the mass spectrometer. Regular cleaning was performed to remove metal oxides and other deposits from surfaces in the ion source. We assume that the frequent cleaning cycles were required because the metal was oxidized by ozone, an effect which was exacerbated by the high temperatures in the ion source. We attempted to minimize the duration of each individual experiment and the flushing period and filled the reaction chamber with He gas during nights and weekends to reduce the exposure of the mass spectrometer to oxidants. At temperatures below 313 K, the mass spectrometer signal required 15 min or more to stabilize after injections, so we did not attempt repeated measurements at low temperatures.

During some of our initial experiments in the development phase of the reactor system, we involuntarily introduced droplets of liquid water into the reaction chamber. After several experiments, we noticed small black dots had formed on the gold coating of the stainless steel caps at the ends of the reactor. We presume that the black material was gold(III)oxide, an oxidation product formed when aqueous OH radicals react with the metallic gold 43, 44. As a consequence of these observations, we installed a nonfritted He introduction line into the gas washing bottle, which reduced turbulence in the water. After this change to the experimental setup, water droplets were no longer observed in the reaction chamber, and no further oxidation of the gold surface was observed.

The possibility that the cVMS or cyclohexane could partition to the chamber walls is a potential complication in the experiments. We were not able to characterize the fraction of cVMS attached to the chamber walls, but we designed our reaction chamber with a size similar to previous successful setups to avoid this problem 20, 21, 22, 23, 24, 25, 26, 27, 28, 29, 30, 31, 32, 33, 34. Furthermore, we did not observe any “dark losses” during the stabilization period of the reaction chamber, which indicates that the loss of the substances to the reaction chamber walls prior to activating the UV lamp was negligible.

In this study, we investigated the potential for regression dilution to introduce bias in the rate constants calculated with Eq. (2) 36. The potential for bias exists because measurement data are used as the independent variable in a regression 39. However, we did not observe any significant bias and therefore conclude that the method described by Cox and Sheppard 36 is appropriate.

Statistical analysis of the temperature dependence of the reactions of D_4_, D_5_, and D_6_ with OH demonstrated that the activation energies were not significantly different. Therefore, we decided to combine our observations in a linear mixed model to obtain a best estimate of the activation energy for reaction of all three cVMS with OH. Our data analysis thus assumes that reactions of all three substances follow the same mechanism, and our observation of fragment ions that may correspond to D_5_TOH as a degradation product of D_6_ supports this assumption. However, it is unlikely that the activation energies are exactly identical in reality. It is more likely that the precision of our measurements was not sufficient to resolve the differences in *E_a_* between D_4_, D_5_, and D_6_.

We extrapolated our measured reaction rates to 298 K using Eq. (6) with our estimated values for *E_a_* and *A* and obtained estimated reaction rates (in units of cm^3^ molecules^−1^ s^−1^) of 1.9 × 10^−12^ for D_4_ (CI: (1.7–2.2) × 10^−12^), 2.6 × 10^−12^ for D_5_ (CI: (2.3–2.9) × 10^−12^), and 2.8 × 10^−12^ for D_6_ (CI: (2.5–3.2) × 10^−12^). Our measured reaction rate constants of D_4_ and D_5_ with the OH radical are 1.9× and 1.7× faster than the previous measurements, i.e. 1.01 ± 0.32 × 10^−12^ for D_4_ and 1.55 ± 0.49 × 10^−12^ for D_5_ 18, and the CIs of the two measurements do not overlap. The reaction rate constant we calculated for D_6_ at 298 K is a factor of 1.8 lower than the value estimated by MacLeod et al. 19, i.e., 5.1 × 10^−12^, with a Junge relationship constructed from spatially variable monitoring data and the D_3_, D_4_, and D_5_ reaction rate constants published by Atkinson [18].

In addition, we also extrapolated the reaction rates to 255 K, which is an estimate of global average tropospheric temperature 45. The estimated reaction rates at this temperature are for D_4_ 1.45 × 10^−12^ (CI: (1.02–1.96) × 10^−12^), for D_5_ 1.94 × 10^−12^ (CI: (1.43–2.61) ×10^−12^), and for D_6_ 2.09 × 10^−12^ (CI: (1.54–2.81) × 10^−12^). These values correspond to atmospheric lifetimes of 8 days for D_4_ (CI: 6–11 days), 6 days for D_5_ (CI: 4–8 days), and 6 days for D_6_ (CI: 4–8 days). A global average OH concentration of 10^6^ molecules cm^−3^ was assumed for the calculation of these atmospheric lifetimes 46.

## Supporting information

Disclaimer: Supplementary materials have been peer‐reviewed but not copyedited.

Supplementary Material
