# Supplementary material for: Rate Constants and Activation Energies for Gas‐Phase Reactions of Three Cyclic Volatile Methyl Siloxanes with the Hydroxyl Radical
Source: Int J Chem Kinet. 2015 Apr 23;47(7):420–8. doi: 10.1002/kin.20919 (PMC5029797; doi:10.1002/kin.20919)
Supplement: Supplementary file 1 — Supplementary Material [file KIN-47-420-s001.pdf]

Supplementary Material for:

Rate constants and activation energies for gas-phase reactions of three cyclic volatile methyl siloxanes with the hydroxyl radical

*Andreas Safron, Michael Strandell, Amelie Kierkegaard, Matthew MacLeod**

Department of Environmental Science and Analytical Chemistry (ACES), Stockholm University, SE-10691 Stockholm, Sweden.

Table SM1: Reaction rate constants for each of the 35 individual experiments calculated using eq. (2) and eq. (5), and coefficients of determination for all linear regression calculations.

		k_{cVMS} calculated with eq. (2) [$cm^3 molecule^{-1} sec^{-1}$]			R^2 for the linear regressions based on eq. (2)			k_{cVMS} calculated with eq. (5) [$cm^3 molecule^{-1} sec^{-1}$]			R^2 for the linear regressions based on eq. (3) and (4)			
Exp. #	T[K]	k_{D4}	k_{D5}	k_{D6}	$\ln(i_{D4,0}/i_{D4,t})$ vs $\ln(i_{cyclohexane,0}/i_{cyclohexane,t})$	$\ln(i_{D5,0}/i_{D5,t})$ vs $\ln(i_{cyclohexane,0}/i_{cyclohexane,t})$	$\ln(i_{D6,0}/i_{D6,t})$ vs $\ln(i_{cyclohexane,0}/i_{cyclohexane,t})$	k_{D4}	k_{D5}	k_{D6}	$\ln(i_{cyclohexane,t})$ vs t	$\ln(i_{D4,t})$ vs t	$\ln(i_{D5,t})$vs t	$\ln(i_{D6,t})$ vs t
267	313	2.40E-12	3.24E-12	3.31E-12	0.999	0.996	0.989	2.40E-12	3.23E-12	3.30E-12	0.999	0.998	0.996	0.989
268	313	2.19E-12	2.97E-12	3.18E-12	0.997	0.992	0.975	2.19E-12	2.97E-12	3.18E-12	0.997	0.995	0.99	0.974
269	313	2.42E-12	3.30E-12	3.35E-12	0.997	0.994	0.989	2.43E-12	3.31E-12	3.36E-12	0.994	0.998	0.995	0.989
270	313	2.04E-12	2.78E-12	3.04E-12	0.996	0.982	0.957	2.03E-12	2.77E-12	3.03E-12	0.996	0.991	0.978	0.952
296	313	1.94E-12	2.54E-12	2.78E-12	0.975	0.918	0.807	1.93E-12	2.53E-12	2.76E-12	0.997	0.968	0.91	0.798
297	313	1.91E-12	2.49E-12	3.23E-12	0.973	0.878	0.802	1.90E-12	2.48E-12	3.21E-12	0.994	0.963	0.869	0.791
298	313	2.15E-12	2.69E-12	2.73E-12	0.994	0.982	0.950	2.15E-12	2.69E-12	2.73E-12	0.997	0.996	0.982	0.948
299	313	1.84E-12	2.48E-12	2.81E-12	0.977	0.942	0.872	1.83E-12	2.46E-12	2.82E-12	0.997	0.965	0.93	0.876
300	313	2.07E-12	2.73E-12	2.71E-12	0.991	0.971	0.929	2.07E-12	2.72E-12	2.71E-12	0.999	0.989	0.967	0.93
301	313	1.79E-12	2.46E-12	2.48E-12	0.979	0.931	0.853	1.78E-12	2.45E-12	2.46E-12	0.997	0.967	0.922	0.841
302	313	2.32E-12	3.07E-12	3.12E-12	0.997	0.993	0.978	2.32E-12	3.06E-12	3.11E-12	0.998	0.996	0.991	0.975
289	323	2.31E-12	3.23E-12	3.61E-12	0.996	0.996	0.991	2.30E-12	3.23E-12	3.60E-12	0.999	0.993	0.993	0.988
290	323	2.13E-12	2.87E-12	3.01E-12	0.931	0.945	0.854	2.08E-12	2.82E-12	2.98E-12	0.986	0.879	0.903	0.831
291	323	1.97E-12	2.66E-12	2.82E-12	0.989	0.985	0.896	1.96E-12	2.65E-12	2.81E-12	0.992	0.978	0.976	0.884
292	323	2.02E-12	2.79E-12	2.98E-12	0.994	0.989	0.940	2.02E-12	2.78E-12	2.97E-12	0.992	0.985	0.979	0.931
271	333	2.95E-12	4.15E-12	4.75E-12	0.996	0.995	0.989	2.96E-12	4.16E-12	4.75E-12	1.000	0.996	0.996	0.989
272	333	2.84E-12	3.80E-12	4.34E-12	0.998	0.998	0.987	2.84E-12	3.80E-12	4.34E-12	0.999	0.995	0.996	0.986
273	333	2.20E-12	2.87E-12	2.98E-12	0.989	0.990	0.958	2.20E-12	2.88E-12	2.99E-12	0.997	0.992	0.993	0.962
274	333	1.67E-12	2.36E-12	2.39E-12	0.968	0.963	0.772	1.67E-12	2.35E-12	2.40E-12	0.998	0.96	0.957	0.773
275	333	2.66E-12	3.52E-12	4.00E-12	0.994	0.992	0.973	2.65E-12	3.51E-12	4.00E-12	0.999	0.988	0.987	0.97
276	333	2.94E-12	4.06E-12	4.86E-12	0.996	0.991	0.964	2.94E-12	4.06E-12	4.86E-12	0.999	0.993	0.989	0.962
287	333	2.55E-12	3.56E-12	3.99E-12	0.999	0.999	0.994	2.55E-12	3.56E-12	3.99E-12	1.000	0.998	0.997	0.993
288	333	2.47E-12	3.46E-12	3.96E-12	0.996	0.996	0.991	2.47E-12	3.46E-12	3.96E-12	1.000	0.994	0.995	0.99
293	343	2.21E-12	3.00E-12	2.95E-12	0.974	0.973	0.952	2.22E-12	2.99E-12	2.93E-12	0.975	0.943	0.93	0.906
294	343	2.55E-12	3.46E-12	3.48E-12	0.980	0.983	0.959	2.58E-12	3.49E-12	3.50E-12	0.987	0.972	0.975	0.949
295	343	2.20E-12	2.97E-12	3.21E-12	0.983	0.984	0.958	2.20E-12	2.98E-12	3.23E-12	0.991	0.968	0.966	0.945
277	353	2.80E-12	3.65E-12	4.22E-12	0.959	0.964	0.933	2.83E-12	3.70E-12	4.28E-12	0.997	0.949	0.957	0.927
282	353	3.30E-12	3.76E-12	4.30E-12	0.896	0.912	0.775	3.29E-12	3.76E-12	4.30E-12	0.988	0.854	0.88	0.746
283	353	2.30E-12	2.91E-12	3.05E-12	0.897	0.919	0.833	2.29E-12	2.91E-12	2.98E-12	0.986	0.852	0.88	0.765
284	353	2.86E-12	3.71E-12	4.31E-12	0.992	0.994	0.989	2.91E-12	3.77E-12	4.39E-12	0.998	0.995	0.996	0.992
285	353	2.70E-12	3.58E-12	3.90E-12	0.990	0.993	0.988	2.73E-12	3.64E-12	3.95E-12	0.998	0.988	0.992	0.986
286	353	3.12E-12	4.13E-12	4.77E-12	0.993	0.996	0.994	3.17E-12	4.19E-12	4.84E-12	0.998	0.993	0.996	0.995
303	353	2.00E-12	2.52E-12	2.32E-12	0.929	0.952	0.903	2.05E-12	2.57E-12	2.36E-12	0.969	0.919	0.934	0.874
304	353	1.71E-12	2.48E-12	2.77E-12	0.916	0.947	0.940	1.75E-12	2.53E-12	2.83E-12	0.972	0.906	0.933	0.927
305	353	2.93E-12	3.61E-12	3.41E-12	0.942	0.913	0.864	2.93E-12	3.60E-12	3.38E-12	0.972	0.887	0.862	0.803

Figure SM1: Arrhenius plot for D_4 using rate constants calculated with eq. (5) and a log-linear regression model.

Exp. #	T [K]	*k* (from eq. 5) [$cm^3 molecule^{-1} sec^{-1}$]	1/T	ln(*k*)
267	313	2.40E-12	0.003195	-26.7556
268	313	2.19E-12	0.003195	-26.8471
269	313	2.43E-12	0.003195	-26.7431
270	313	2.03E-12	0.003195	-26.923
296	313	1.93E-12	0.003195	-26.9735
297	313	1.90E-12	0.003195	-26.9892
298	313	2.15E-12	0.003195	-26.8656
299	313	1.83E-12	0.003195	-27.0267
300	313	2.07E-12	0.003195	-26.9035
301	313	1.78E-12	0.003195	-27.0544
302	313	2.32E-12	0.003195	-26.7895
289	323	2.30E-12	0.003096	-26.7981
290	323	2.08E-12	0.003096	-26.8987
291	323	1.96E-12	0.003096	-26.9581
292	323	2.02E-12	0.003096	-26.9279
271	333	2.96E-12	0.003003	-26.5458
272	333	2.84E-12	0.003003	-26.5872
273	333	2.20E-12	0.003003	-26.8426
274	333	1.67E-12	0.003003	-27.1182
275	333	2.65E-12	0.003003	-26.6565
276	333	2.94E-12	0.003003	-26.5526
287	333	2.55E-12	0.003003	-26.6949
288	333	2.47E-12	0.003003	-26.7268
293	343	2.22E-12	0.002915	-26.8335
294	343	2.58E-12	0.002915	-26.6832
295	343	2.20E-12	0.002915	-26.8426
277	353	2.83E-12	0.002833	-26.5907
282	353	3.29E-12	0.002833	-26.4401
283	353	2.29E-12	0.002833	-26.8025
284	353	2.91E-12	0.002833	-26.5629
285	353	2.73E-12	0.002833	-26.6267
286	353	3.17E-12	0.002833	-26.4773
303	353	2.05E-12	0.002833	-26.9132
304	353	1.75E-12	0.002833	-27.0714
305	353	2.93E-12	0.002833	-26.556

D_4

SUMMARY OUTPUT

Regression Statistics	
Multiple R	0.508843277
R Square	0.258921481
Adjusted R Square	0.236464556
Standard Error	0.157391748
Observations	35

ANOVA

	df	*SS*	*MS*	*F*	*Significance F*
Regression	1	0.285615463	0.285615463	11.5296944	0.001799609
Residual	33	0.817481362	0.024772162		
Total	34	1.103096825			

	Coefficients	*Standard Error*	*t Stat*	*P-value*	*Lower 95%*	*Upper 95%*	*Lower 95.0%*	*Upper 95.0%*
Intercept	-24.8702417	0.565401549	-43.9868652	7.03862E-31	-26.0205598	-23.7199236	-26.0205598	-23.7199236
X Variable 1	-634.442711	186.8458748	-3.39554037	0.001799609	-1014.5835	-254.30192	-1014.5835	-254.30192

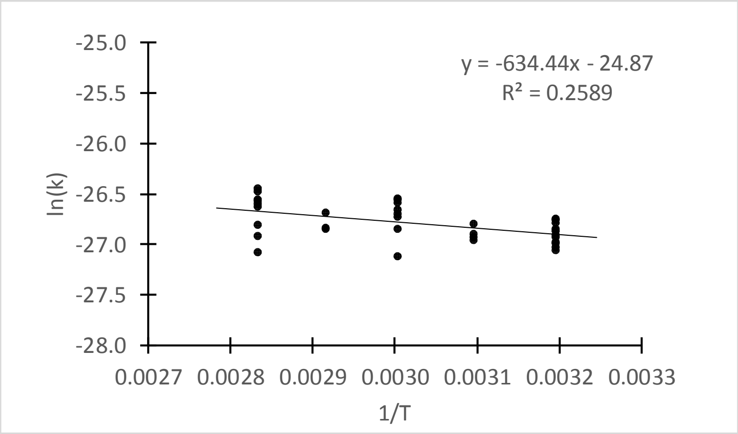

	Value	Lower 95%	Upper 95%
Ea (J/mol)	-5275	-8435	-2114
A	1.58E-11	5.01E-12	5.00E-11

Figure SM1: Arrhenius plot for D_5 using rate constants calculated with eq. (5) and a log-linear regression model.

Exp. #	T [K]	*k* (from eq. 5) [$cm^3 molecule^{-1} sec^{-1}$]	1/T	ln(*k*)
267	313	3.23E-12	0.003195	-26.4585
268	313	2.97E-12	0.003195	-26.5425
269	313	3.31E-12	0.003195	-26.4341
270	313	2.77E-12	0.003195	-26.6122
296	313	2.53E-12	0.003195	-26.7028
297	313	2.48E-12	0.003195	-26.7228
298	313	2.69E-12	0.003195	-26.6415
299	313	2.46E-12	0.003195	-26.7309
300	313	2.72E-12	0.003195	-26.6304
301	313	2.45E-12	0.003195	-26.7349
302	313	3.06E-12	0.003195	-26.5126
289	323	3.23E-12	0.003096	-26.4585
290	323	2.82E-12	0.003096	-26.5943
291	323	2.65E-12	0.003096	-26.6565
292	323	2.78E-12	0.003096	-26.6086
271	333	4.16E-12	0.003003	-26.2055
272	333	3.80E-12	0.003003	-26.296
273	333	2.88E-12	0.003003	-26.5732
274	333	2.35E-12	0.003003	-26.7766
275	333	3.51E-12	0.003003	-26.3754
276	333	4.06E-12	0.003003	-26.2298
287	333	3.56E-12	0.003003	-26.3613
288	333	3.46E-12	0.003003	-26.3898
293	343	2.99E-12	0.002915	-26.5357
294	343	3.49E-12	0.002915	-26.3811
295	343	2.98E-12	0.002915	-26.5391
277	353	3.70E-12	0.002833	-26.3227
282	353	3.76E-12	0.002833	-26.3066
283	353	2.91E-12	0.002833	-26.5629
284	353	3.77E-12	0.002833	-26.3039
285	353	3.64E-12	0.002833	-26.339
286	353	4.19E-12	0.002833	-26.1983
303	353	2.57E-12	0.002833	-26.6871
304	353	2.53E-12	0.002833	-26.7028
305	353	3.60E-12	0.002833	-26.3501

D_5

SUMMARY OUTPUT

Regression Statistics	
Multiple R	0.470842846
R Square	0.221692986
Adjusted R Square	0.198107925
Standard Error	0.151841344
Observations	35

ANOVA

	df	*SS*	*MS*	*F*	*Significance F*
Regression	1	0.21671802	0.21671802	9.399720672	0.004308042
Residual	33	0.76084119	0.023055794		
Total	34	0.97755921			

	Coefficients	*Standard Error*	*t Stat*	*P-value*	*Lower 95%*	*Upper 95%*	*Lower 95.0%*	*Upper 95.0%*
Intercept	-24.8288911	0.545462717	-45.5189517	2.3149E-31	-25.9386433	-23.7191388	-25.9386433	-23.7191388
X Variable 1	-552.64859	180.2567731	-3.06589639	0.004308042	-919.383752	-185.913427	-919.383752	-185.913427

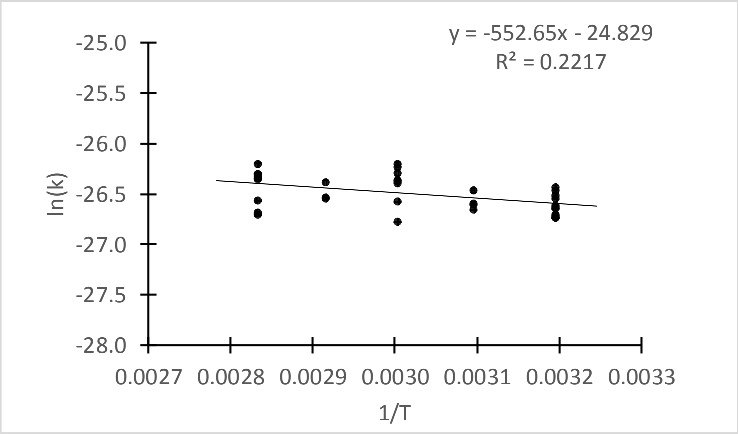

	Value	Lower 95%	Upper 95%
Ea (J/mol)	-4595	-7644	-1546
A	1.65E-11	5.43E-12	5.00E-11

Figure SM1: Arrhenius plot for D_6 using rate constants calculated with eq. (5) and a log-linear regression model.

Exp. #	T [K]	*k* (from eq. 5) [$cm^3 molecule^{-1} sec^{-1}$]	1/T	ln(*k*)	**D_6**
267	313	3.30E-12	0.003195	-26.4371	
268	313	3.18E-12	0.003195	-26.4741	
269	313	3.36E-12	0.003195	-26.4191	
270	313	3.03E-12	0.003195	-26.5225	
296	313	2.76E-12	0.003195	-26.6158	
297	313	3.21E-12	0.003195	-26.4648	
298	313	2.73E-12	0.003195	-26.6267	
299	313	2.82E-12	0.003195	-26.5943	
300	313	2.71E-12	0.003195	-26.6341	
301	313	2.46E-12	0.003195	-26.7309	
302	313	3.11E-12	0.003195	-26.4964	
289	323	3.60E-12	0.003096	-26.3501	
290	323	2.98E-12	0.003096	-26.5391	
291	323	2.81E-12	0.003096	-26.5978	
292	323	2.97E-12	0.003096	-26.5425	
271	333	4.75E-12	0.003003	-26.0729	
272	333	4.34E-12	0.003003	-26.1631	
273	333	2.99E-12	0.003003	-26.5357	
274	333	2.40E-12	0.003003	-26.7556	
275	333	4.00E-12	0.003003	-26.2447	
276	333	4.86E-12	0.003003	-26.05	
287	333	3.99E-12	0.003003	-26.2472	
288	333	3.96E-12	0.003003	-26.2548	
293	343	2.93E-12	0.002915	-26.556	
294	343	3.50E-12	0.002915	-26.3783	
295	343	3.23E-12	0.002915	-26.4585	
277	353	4.28E-12	0.002833	-26.1771	
282	353	4.30E-12	0.002833	-26.1724	
283	353	2.98E-12	0.002833	-26.5391	
284	353	4.39E-12	0.002833	-26.1517	
285	353	3.95E-12	0.002833	-26.2573	
286	353	4.84E-12	0.002833	-26.0541	
303	353	2.36E-12	0.002833	-26.7724	
304	353	2.83E-12	0.002833	-26.5907	
305	353	3.38E-12	0.002833	-26.4131	

SUMMARY OUTPUT

Regression Statistics	
Multiple R	0.394480243
R Square	0.155614662
Adjusted R Square	0.130027227
Standard Error	0.190568017
Observations	35

ANOVA

	df	*SS*	*MS*	*F*	*Significance F*
Regression	1	0.220863424	0.220863424	6.081682868	0.019026298
Residual	33	1.198433583	0.036316169		
Total	34	1.419297007			

	Coefficients	*Standard Error*	*t Stat*	*P-value*	*Lower 95%*	*Upper 95%*	*Lower 95.0%*	*Upper 95.0%*
Intercept	-24.7390454	0.684581327	-36.137482	4.06014E-28	-26.1318366	-23.3462542	-26.1318366	-23.3462542
X Variable 1	-557.909112	226.2307157	-2.46610682	0.019026298	-1018.17896	-97.63926	-1018.17896	-97.63926

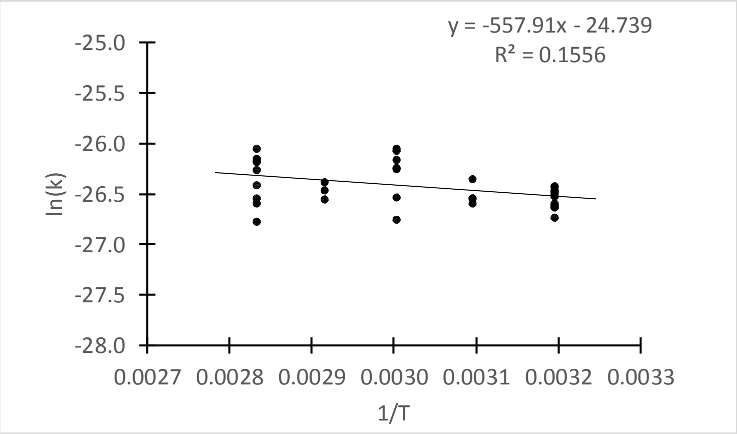

	Value	Lower 95%	Upper 95%
Ea (J/mol)	-4638	-8465	-812
A	1.80E-11	4.48E-12	7.26E-11

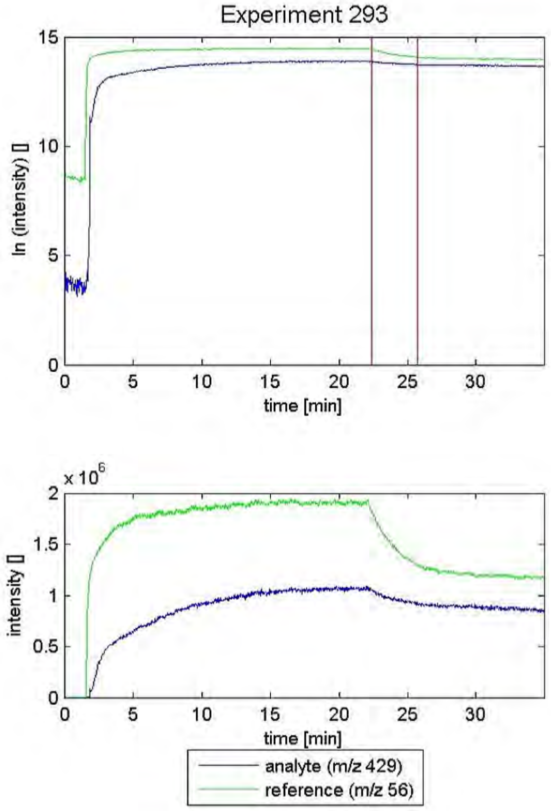

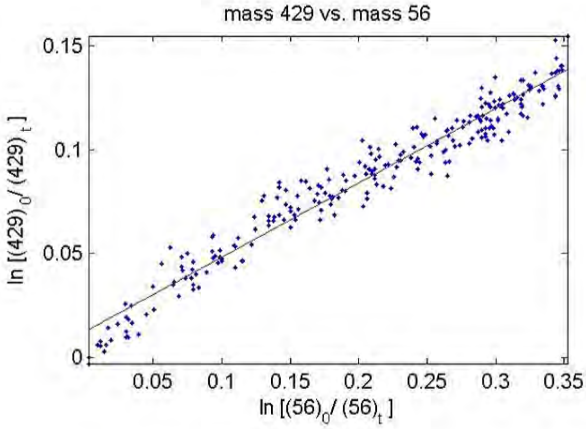

y= 0.012 + 0.358 * x
r= 0.976 n= 251
oven temperature: 70°C
analyte rate constant:
2.95e-12 cm^3 $molec^{-3}$ sec^{-1}
uncertainty range:
[2.68e-12;3.25e-12]
ln(rate)=-26.55
reference shift: 0.0 minutes

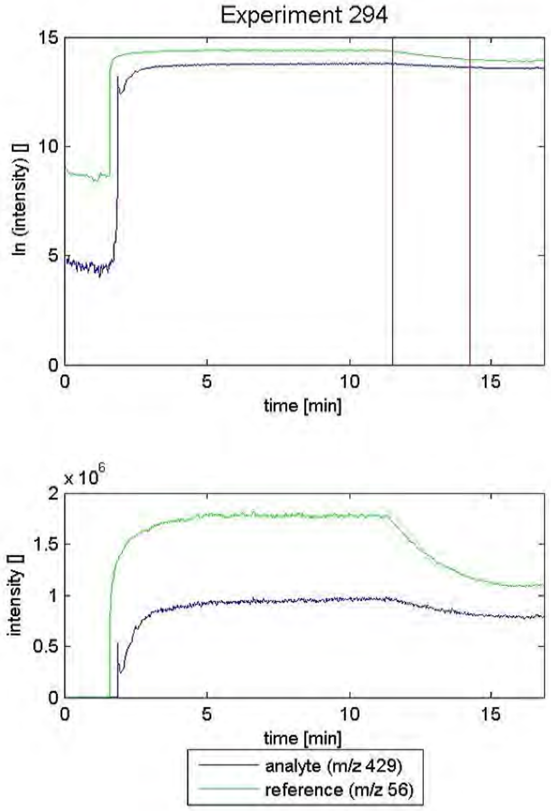

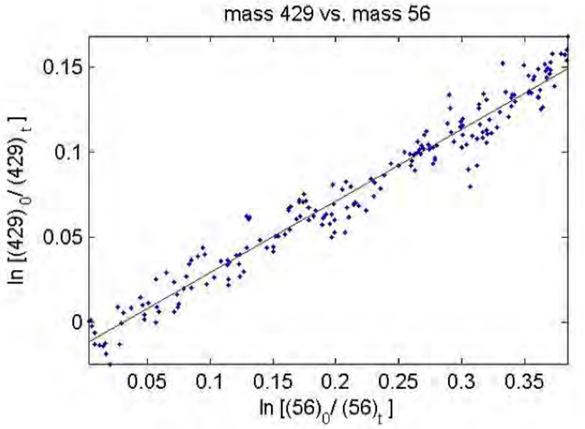

y= -0.013 + 0.422 * x
r= 0.979 n= 201
oven temperature: 70°C
analyte rate constant:
3.48e-12 cm^3 $molec^{-3}$ sec^{-1}
uncertainty range:
[3.16e-12;3.83e-12]
ln(rate)=-26.39
reference shift: 0.0 minutes

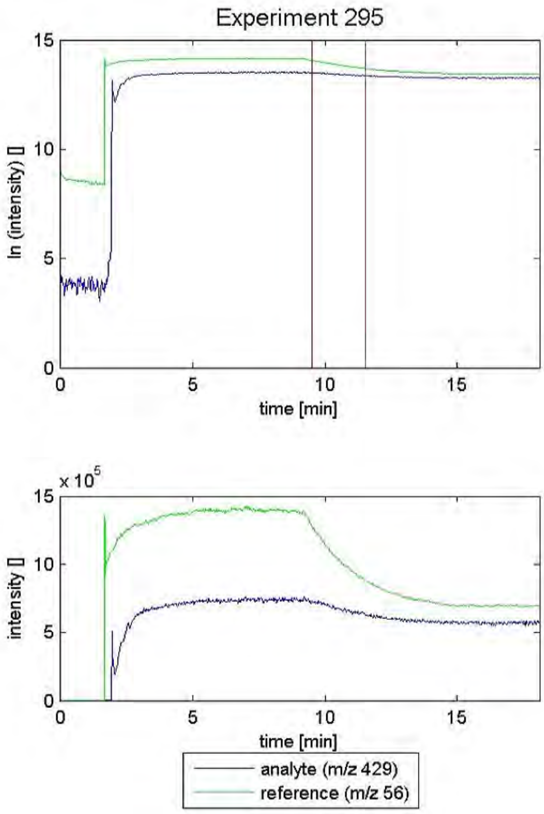

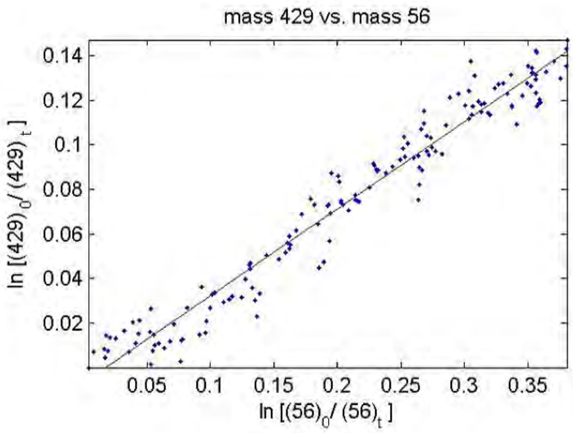

y= -0.007 + 0.390 * x
r= 0.979 n= 151
oven temperature: 70°C
analyte rate constant:
3.21e-12 cm^3 $molec^{-3}$ sec^{-1}
uncertainty range:
[2.92e-12;3.54e-12]
ln(rate)=-26.46
reference shift: 0.0 minutes

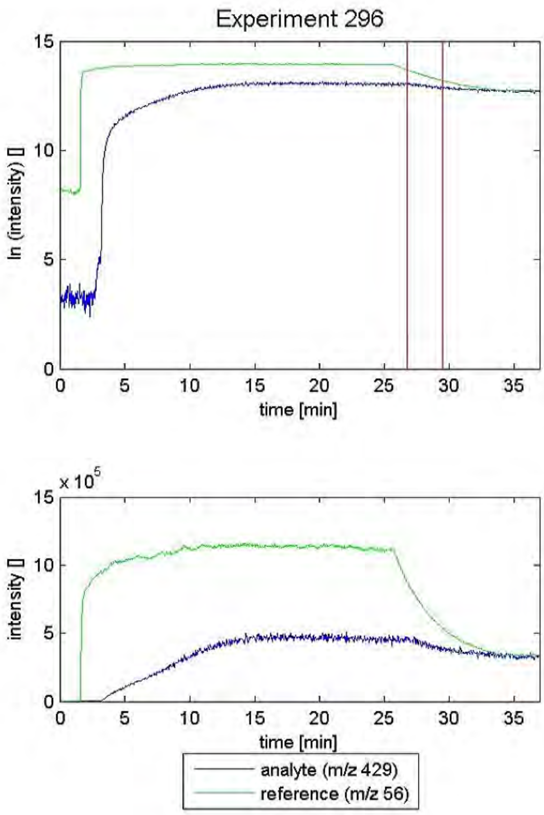

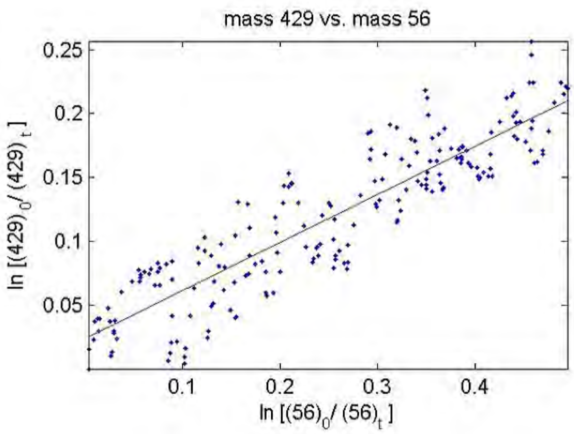

y= 0.024 + 0.377 * x
r= 0.898 n= 201
oven temperature: 40°C
analyte rate constant:
2.78e-12 cm^3 $molec^{-3}$ sec^{-1}
uncertainty range:
[2.50e-12;3.09e-12]
ln(rate)=-26.61
reference shift: 0.0 minutes

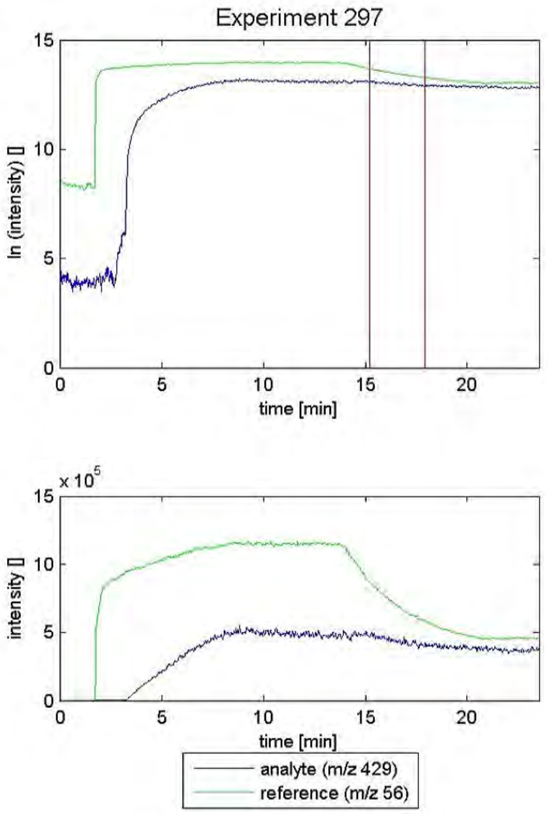

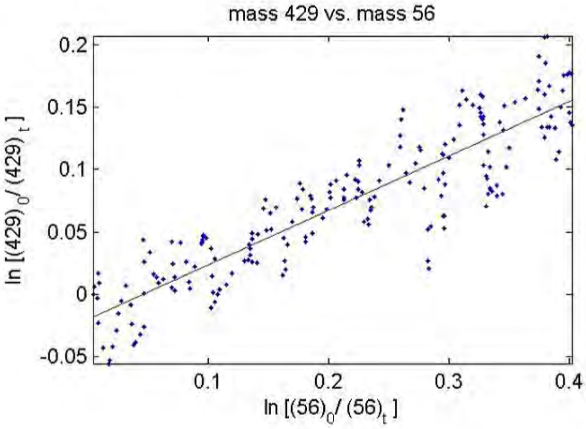

y= -0.020 + 0.437 * x
r= 0.895 n= 201
oven temperature: 40°C
analyte rate constant:
3.23e-12 cm^3 $molec^{-3}$ sec^{-1}
uncertainty range:
[2.90e-12;3.59e-12]
ln(rate)=-26.46
reference shift: 0.0 minutes

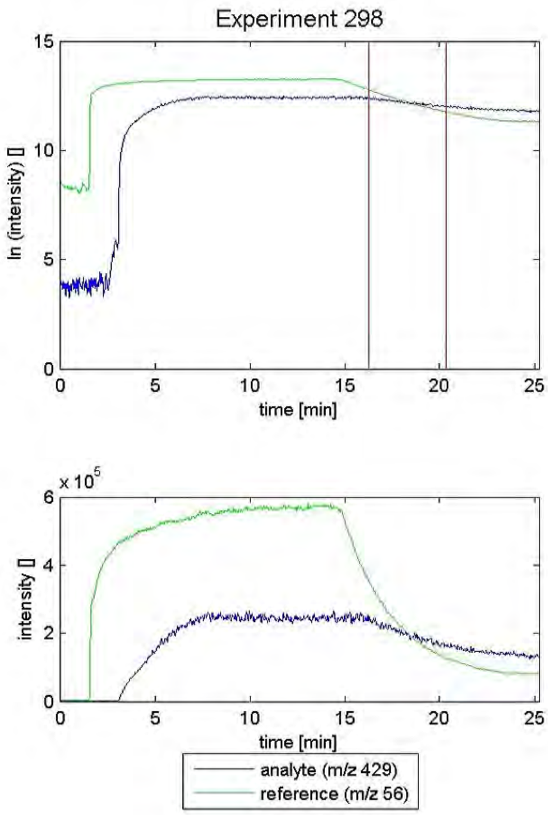

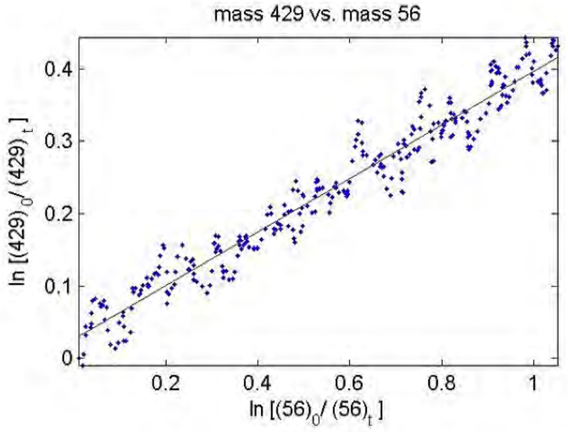

y= 0.026 + 0.371 * x
r= 0.975 n= 301
oven temperature: 40°C
analyte rate constant:
2.73e-12 cm^3 $molec^{-3}$ sec^{-1}
uncertainty range:
[2.46e-12;3.04e-12]
ln(rate)=-26.63
reference shift: 0.0 minutes

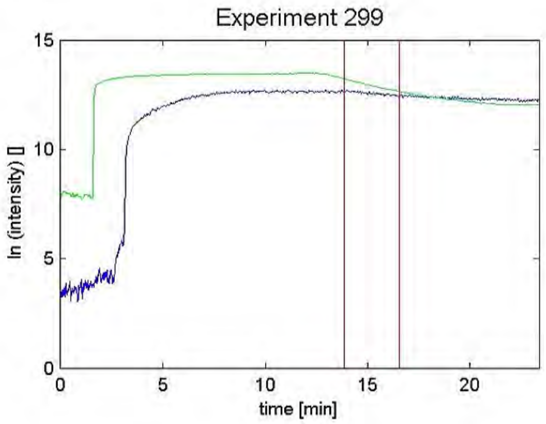

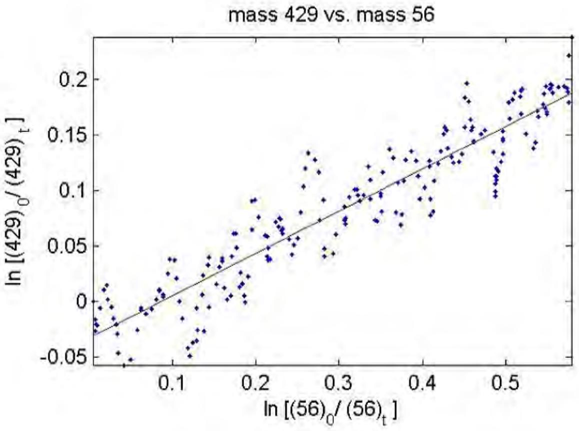

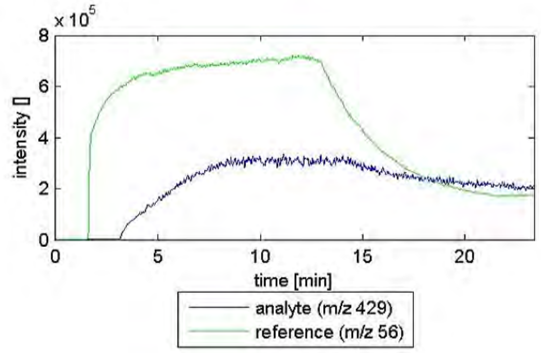

y= -0.033 + 0.381 * x
r= 0.934 n= 201
oven temperature: 40°C
analyte rate constant:
2.81e-12 cm^3 $molec^{-3}$ sec^{-1}
uncertainty range:
[2.53e-12;3.13e-12]
ln(rate)=-26.60
reference shift: 0.0 minutes

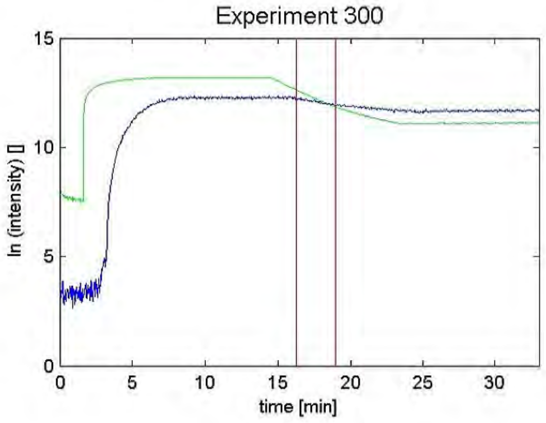

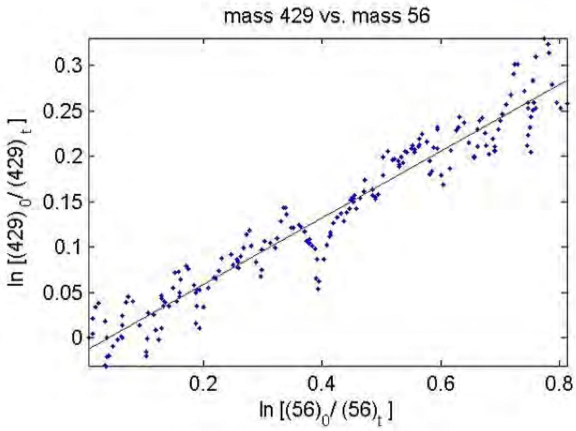

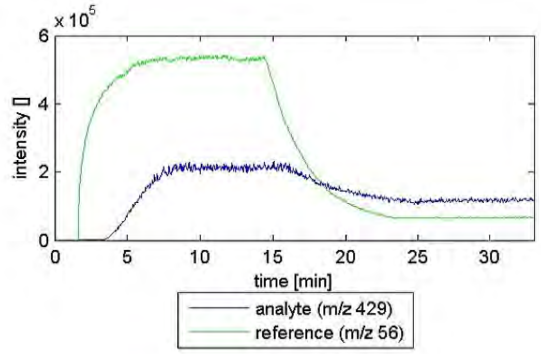

y= -0.015 + 0.368 * x
r= 0.964 n= 201
oven temperature: 40°C
analyte rate constant:
2.71e-12 cm^3 $molec^{-3}$ sec^{-1}
uncertainty range:
[2.44e-12;3.02e-12]
ln(rate)=-26.63
reference shift: 0.0 minutes

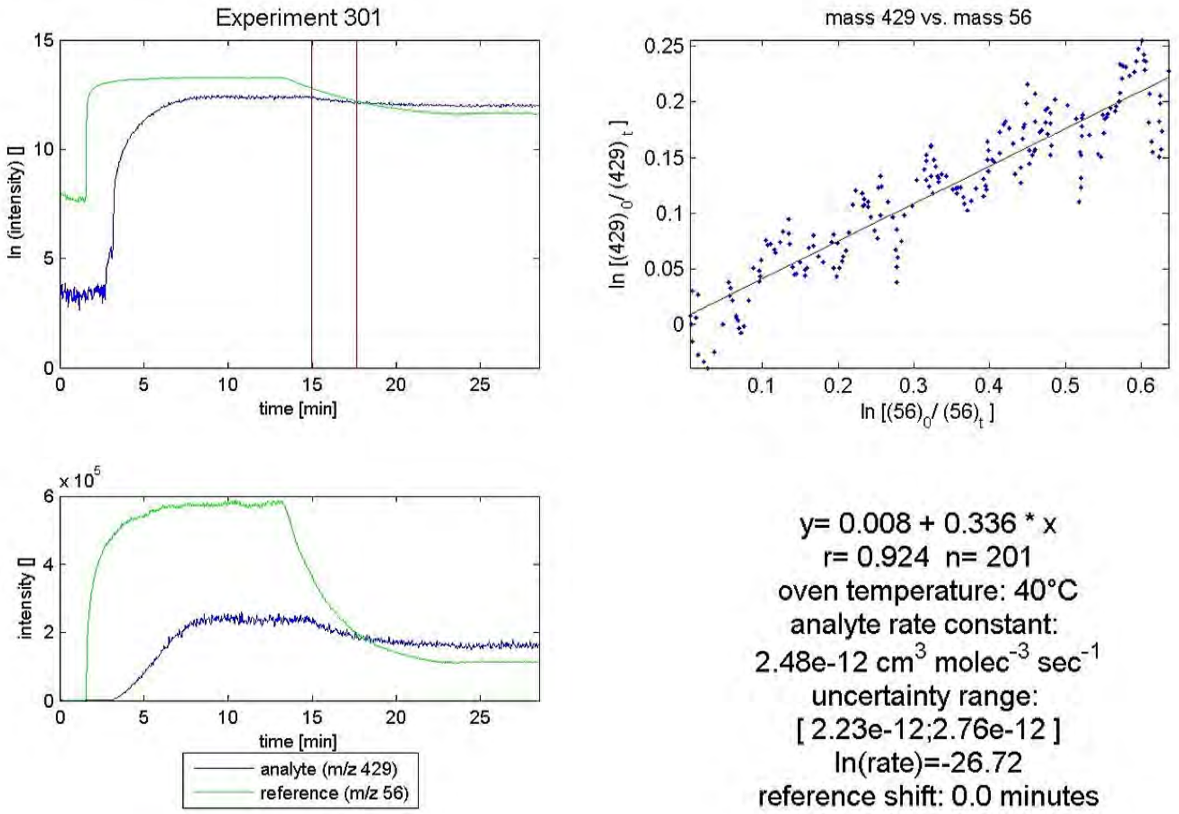

y= 0.008 + 0.336 * x
r= 0.924 n= 201
oven temperature: 40°C
analyte rate constant:
2.48e-12 cm^3 $molec^{-3}$ sec^{-1}
uncertainty range:
[2.23e-12;2.76e-12]
ln(rate)=-26.72
reference shift: 0.0 minutes

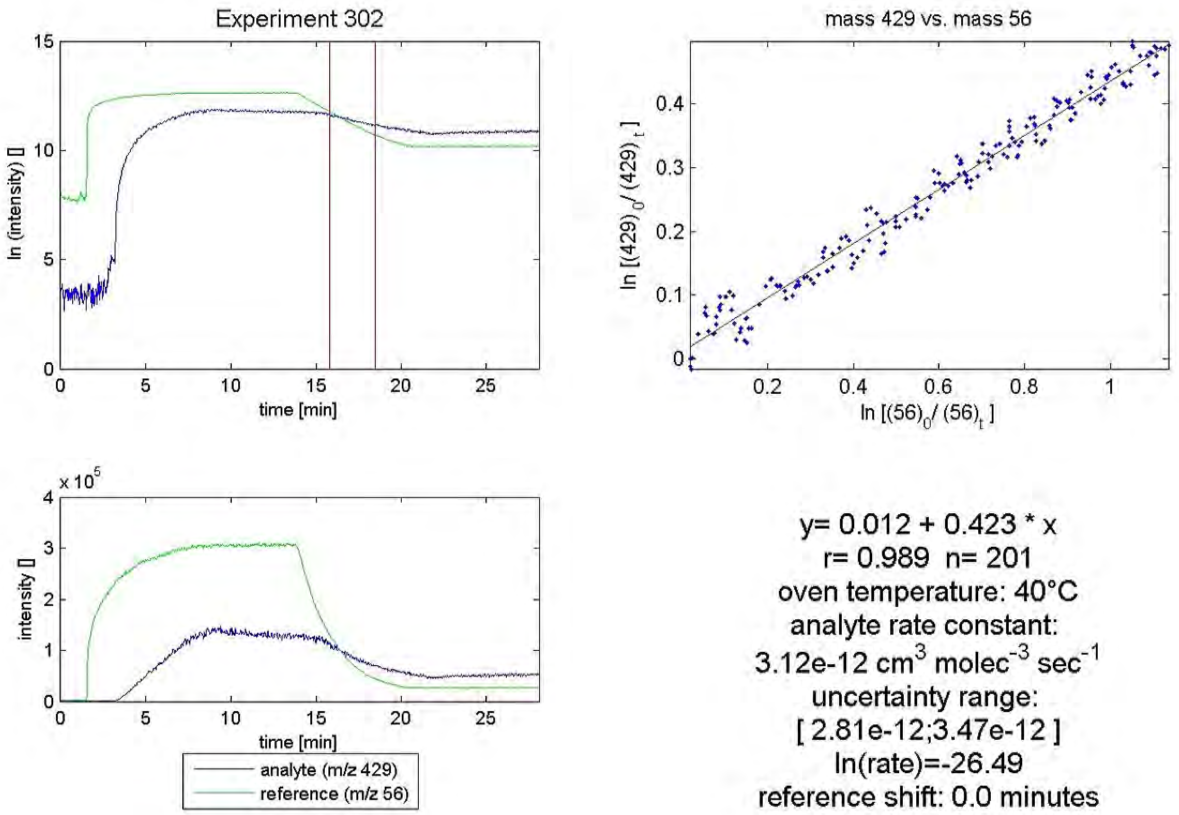

y= 0.012 + 0.423 * x
r= 0.989 n= 201
oven temperature: 40°C
analyte rate constant:
3.12e-12 cm^3 $molec^{-3}$ sec^{-1}
uncertainty range:
[2.81e-12;3.47e-12]
ln(rate)=-26.49
reference shift: 0.0 minutes

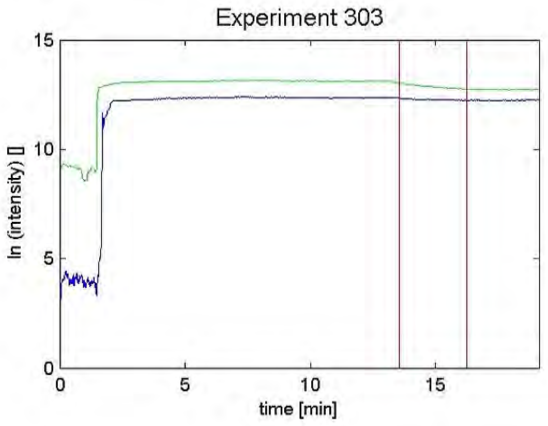

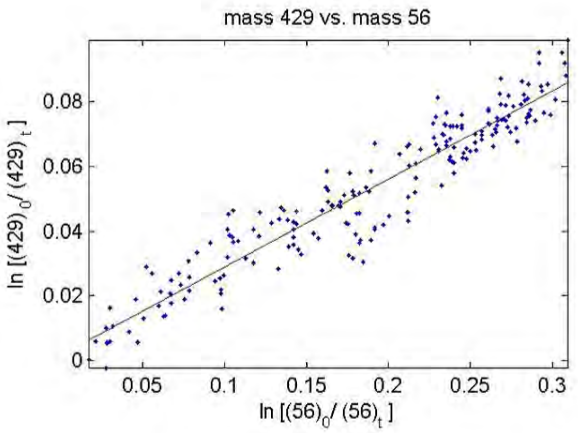

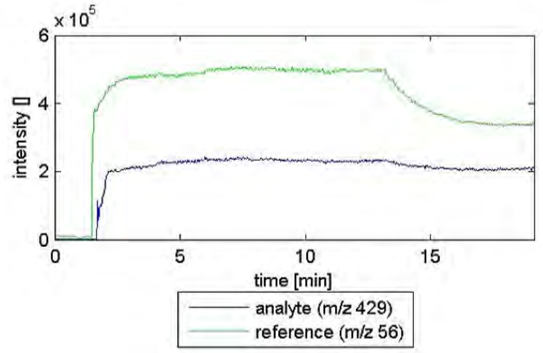

y= 0.002 + 0.272 * x
r= 0.950 n= 201
oven temperature: 80°C
analyte rate constant:
2.32e-12 cm^3 molec^{-3} sec^{-1}
uncertainty range:
[2.12e-12;2.55e-12]
ln(rate)=-26.79
reference shift: 0.0 minutes

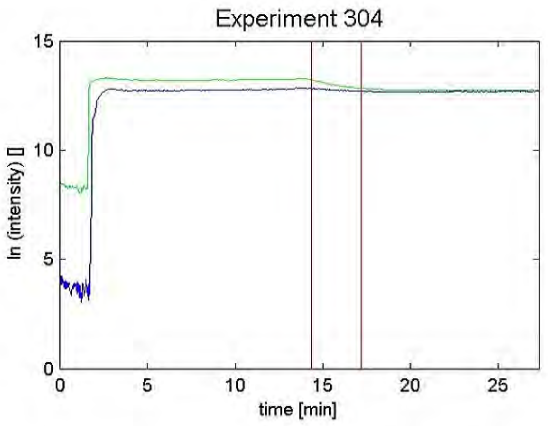

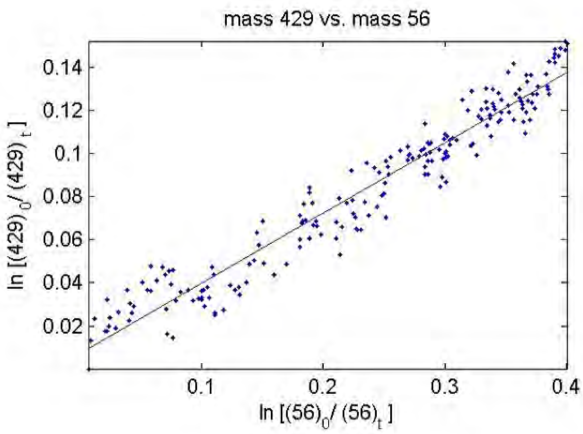

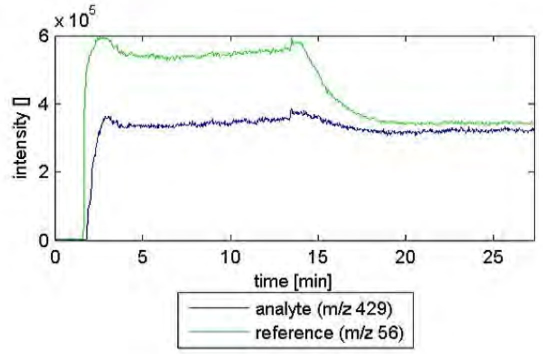

y= 0.007 + 0.325 * x
r= 0.969 n= 201
oven temperature: 80°C
analyte rate constant:
2.77e-12 cm^3 molec^{-3} sec^{-1}
uncertainty range:
[2.53e-12;3.05e-12]
ln(rate)=-26.61
reference shift: 0.0 minutes

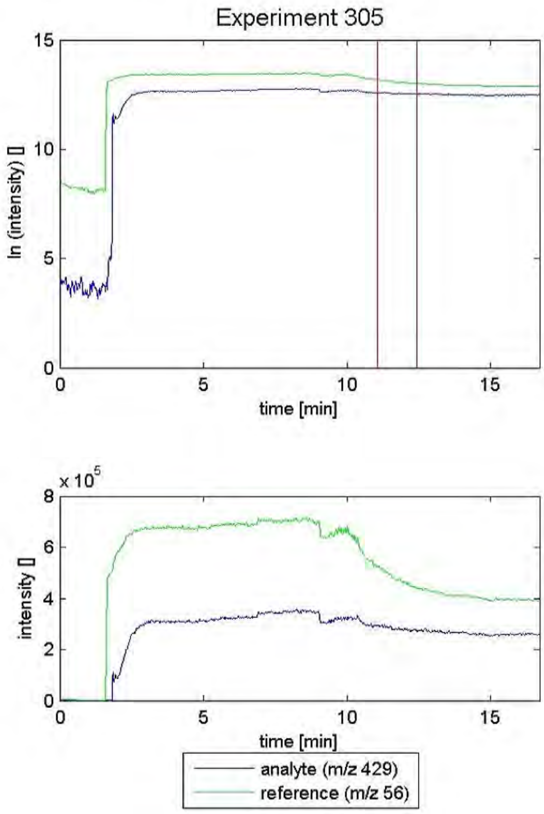

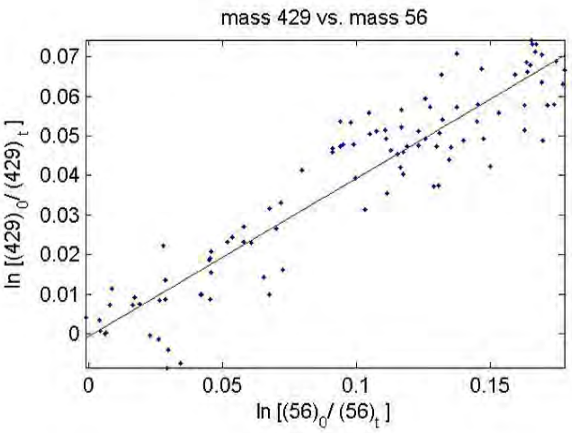

y= -0.001 + 0.399 * x
r= 0.930 n= 101
oven temperature: 80°C
analyte rate constant:
3.41e-12 cm^3 $molec^{-3}$ sec^{-1}
uncertainty range:
[3.11e-12;3.74e-12]
ln(rate)=-26.40
reference shift: 0.0 minutes

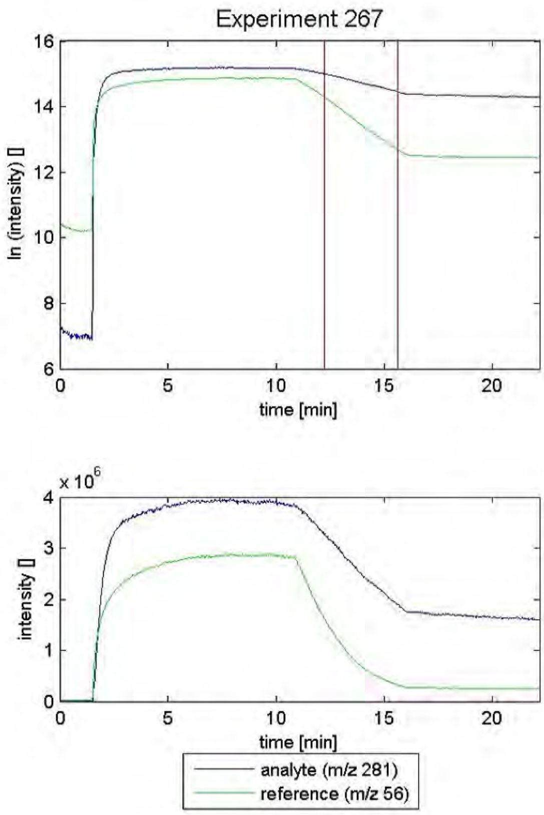

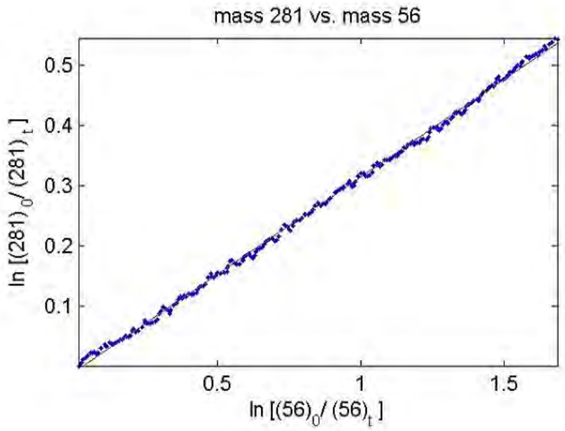

y= -0.010 + 0.325 * x
r= 0.999 n= 251
oven temperature: 40°C
analyte rate constant:
2.40e-12 cm^3 $molec^{-3}$ sec^{-1}
uncertainty range:
[2.16e-12;2.67e-12]
ln(rate)=-26.76
reference shift: 0.0 minutes

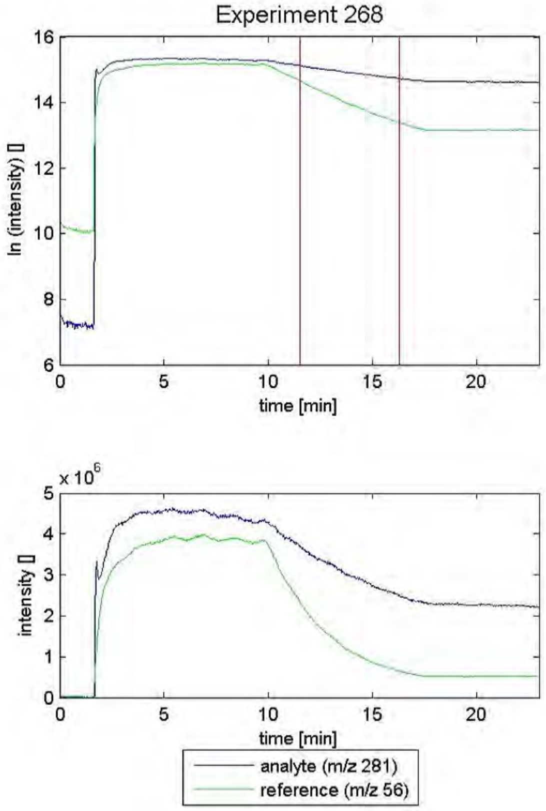

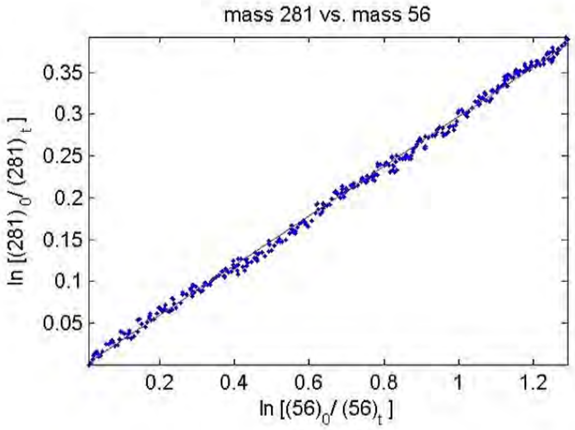

y= -0.000 + 0.297 * x
r= 0.999 n= 351
oven temperature: 40°C
analyte rate constant:
2.19e-12 cm^3 $molec^{-3}$ sec^{-1}
uncertainty range:
[1.97e-12;2.44e-12]
ln(rate)=-26.85
reference shift: 0.0 minutes

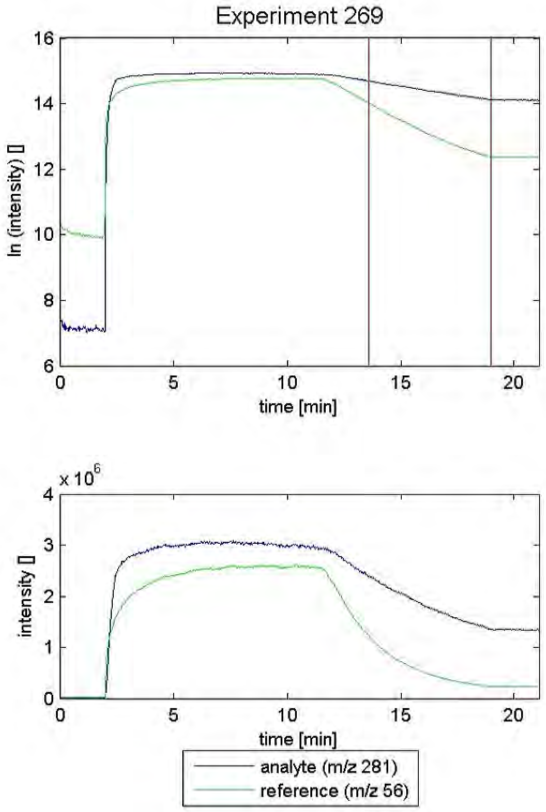

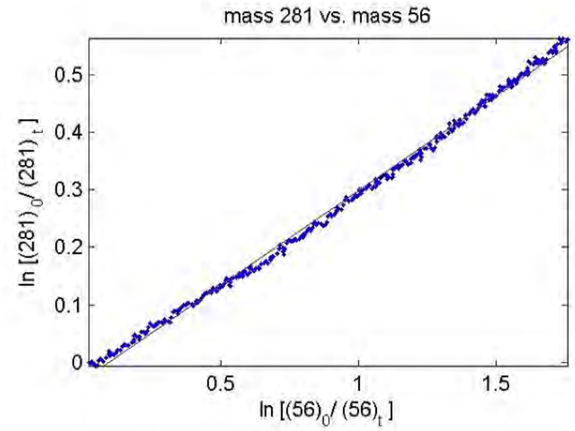

y= -0.030 + 0.328 * x
r= 0.998 n= 401
oven temperature: 40°C
analyte rate constant:
2.42e-12 cm^3 $molec^{-3}$ sec^{-1}
uncertainty range:
[2.18e-12;2.69e-12]
ln(rate)=-26.75
reference shift: 0.0 minutes

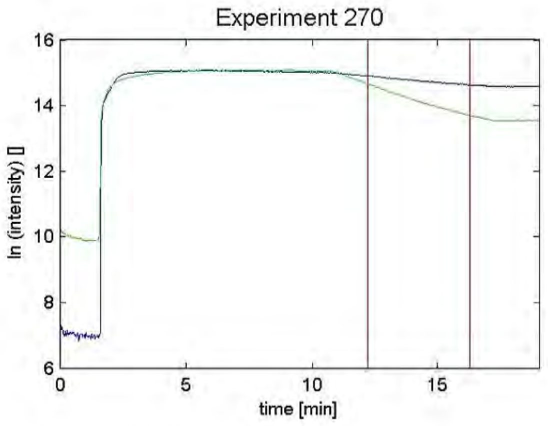

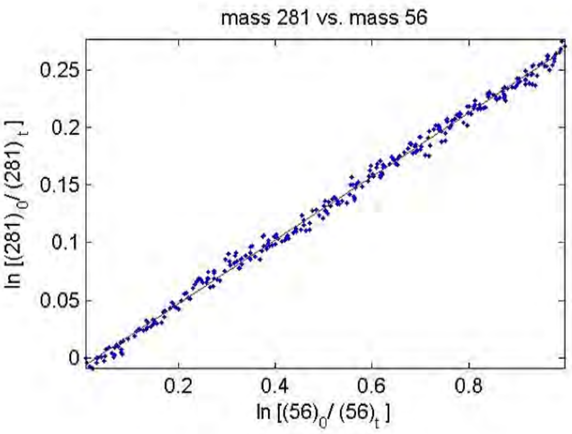

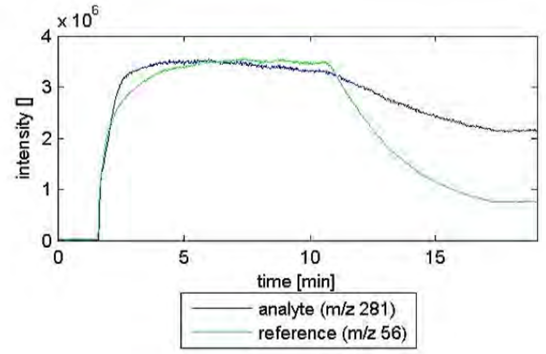

y= -0.008 + 0.276 * x

r= 0.998 n= 301

oven temperature: 40°C

analyte rate constant:

2.04e-12 cm^3 $molec^{-3}$ sec^{-1}

uncertainty range:

[1.83e-12;2.26e-12]

ln(rate)=-26.92

reference shift: 0.0 minutes

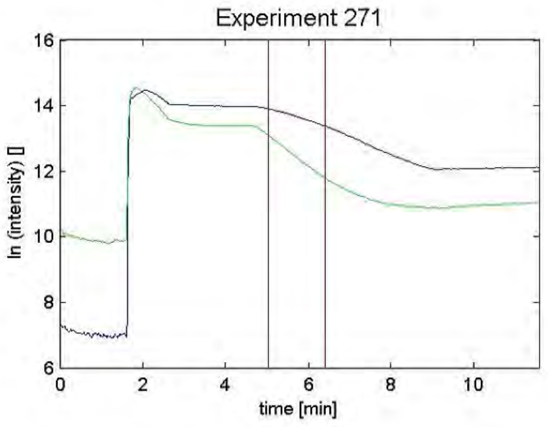

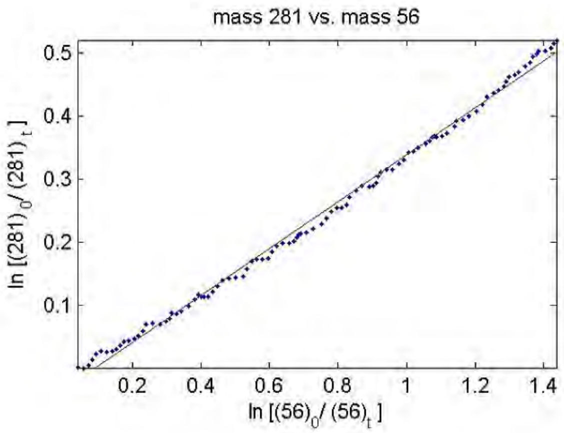

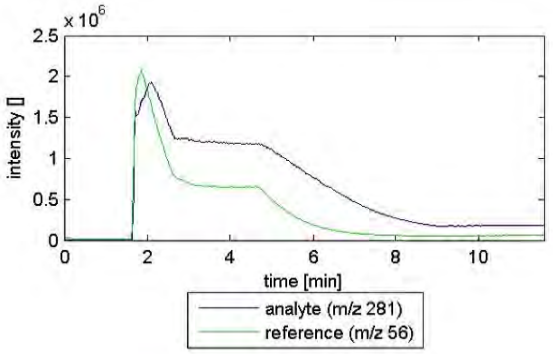

y= -0.034 + 0.372 * x

r= 0.998 n= 101

oven temperature: 60°C

analyte rate constant:

2.95e-12 cm^3 $molec^{-3}$ sec^{-1}

uncertainty range:

[2.68e-12;3.26e-12]

ln(rate)=-26.55

reference shift: 0.0 minutes

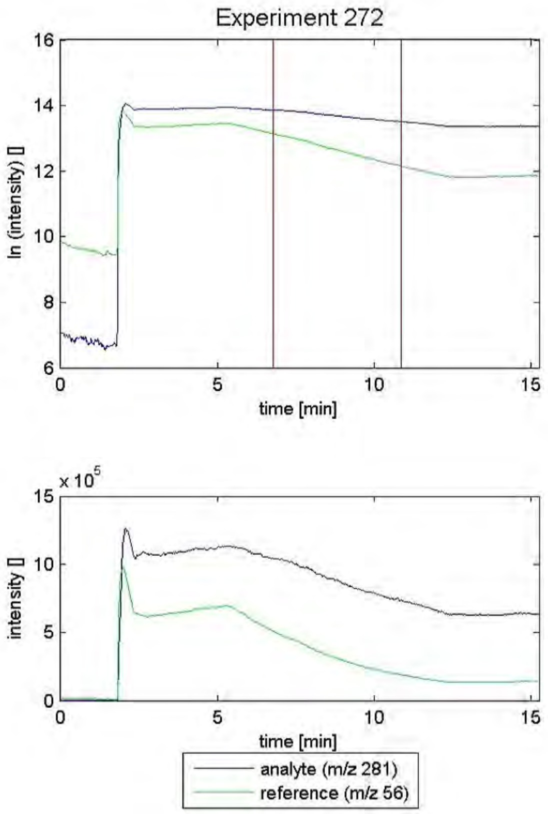

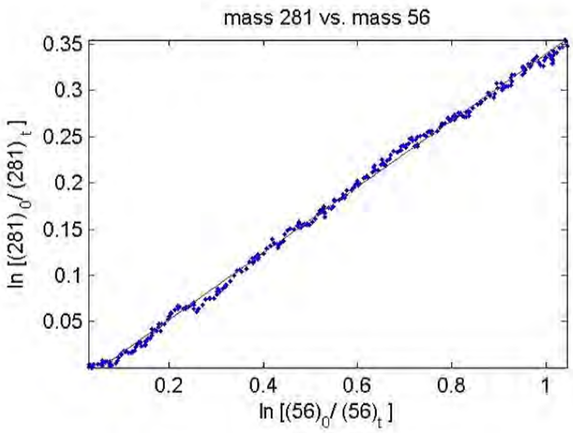

y= -0.019 + 0.358 * x
r= 0.999 n= 301
oven temperature: 60°C
analyte rate constant:
2.84e-12 cm^3 $molec^{-3}$ sec^{-1}
uncertainty range:
[2.57e-12;3.14e-12]
ln(rate)=-26.59
reference shift: 0.0 minutes

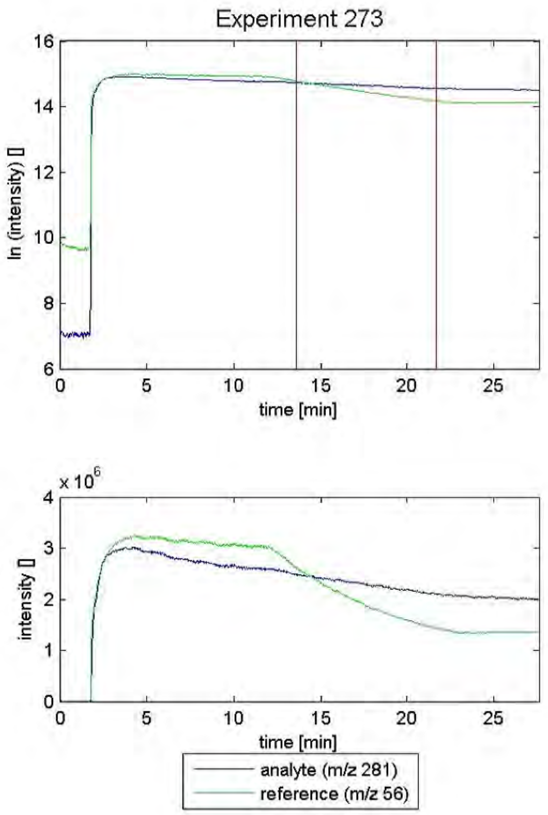

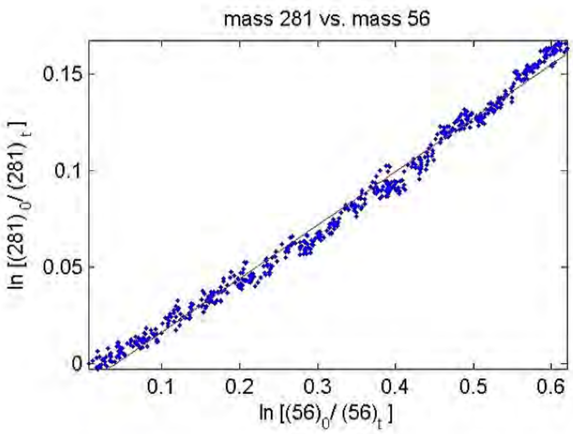

y= -0.011 + 0.277 * x
r= 0.995 n= 601
oven temperature: 60°C
analyte rate constant:
2.20e-12 cm^3 $molec^{-3}$ sec^{-1}
uncertainty range:
[1.99e-12;2.43e-12]
ln(rate)=-26.84
reference shift: 0.0 minutes

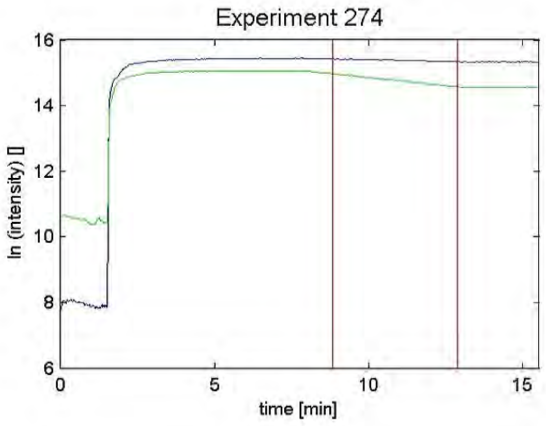

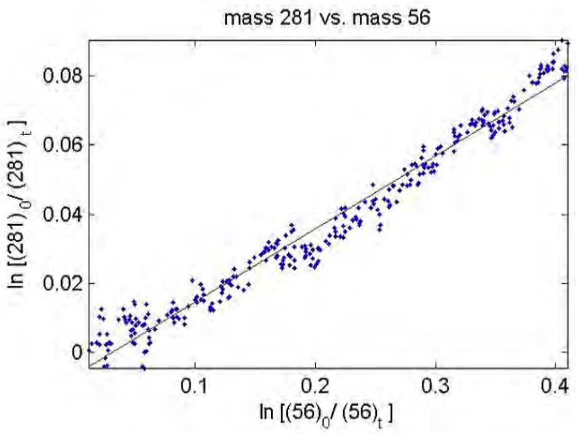

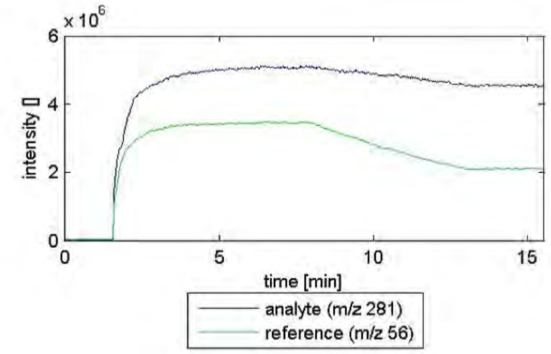

y= -0.006 + 0.211 * x
r= 0.984 n= 301
oven temperature: 60°C
analyte rate constant:
1.67e-12 cm^3 molec^{-3} sec^{-1}
uncertainty range:
[1.52e-12;1.85e-12]
ln(rate)=-27.12
reference shift: 0.0 minutes

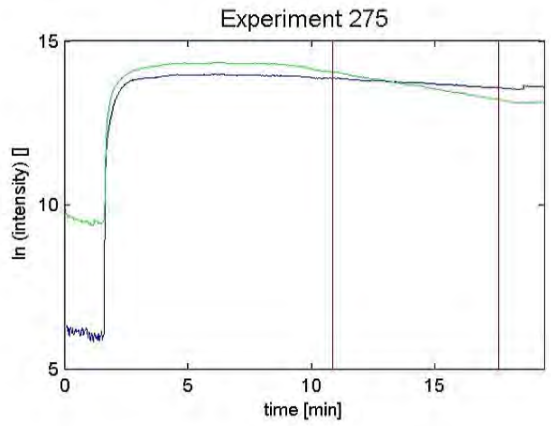

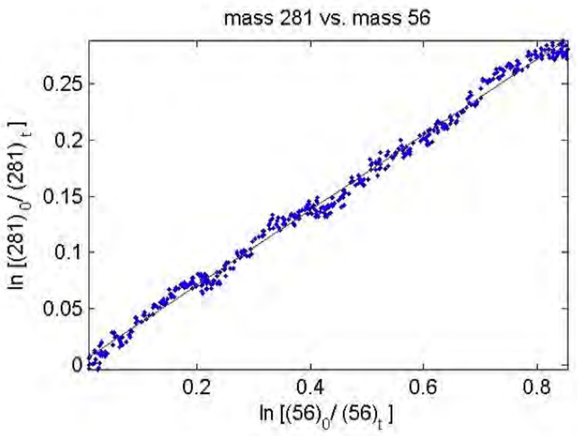

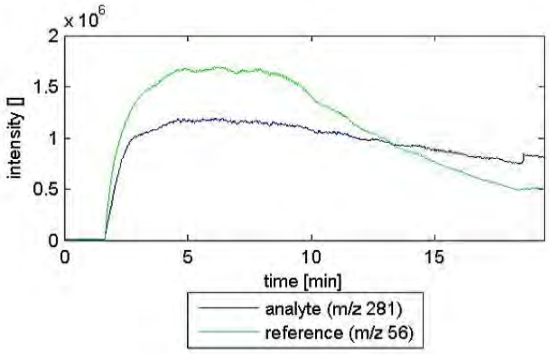

y= 0.004 + 0.334 * x
r= 0.997 n= 501
oven temperature: 60°C
analyte rate constant:
2.66e-12 cm^3 molec^{-3} sec^{-1}
uncertainty range:
[2.41e-12;2.93e-12]
ln(rate)=-26.65
reference shift: 0.0 minutes

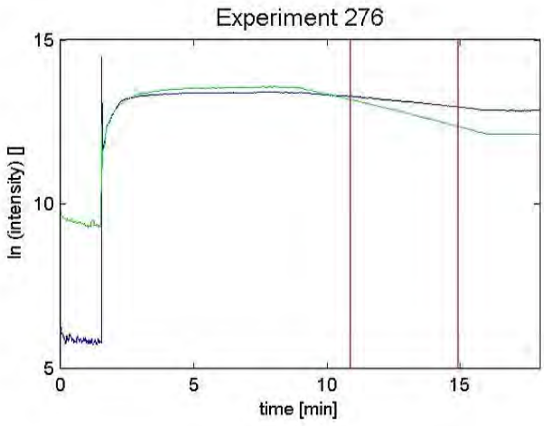

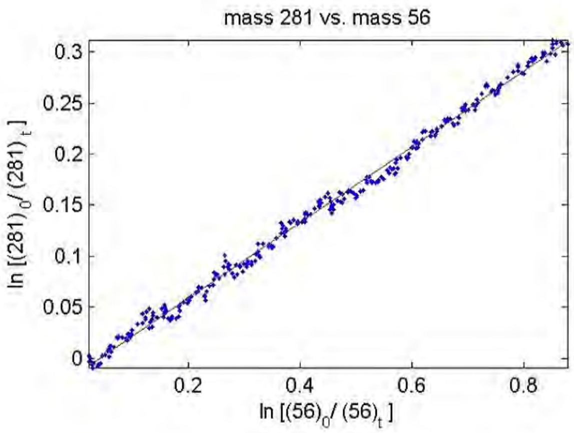

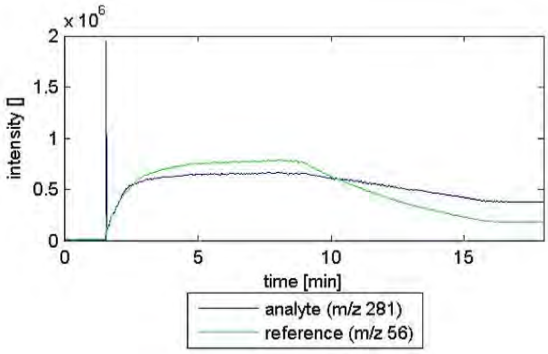

y= -0.015 + 0.370 * x
r= 0.998 n= 301
oven temperature: 60°C
analyte rate constant:
2.94e-12 cm^3 $molec^{-3}$ sec^{-1}
uncertainty range:
[2.66e-12;3.25e-12]
ln(rate)=-26.55
reference shift: 0.0 minutes

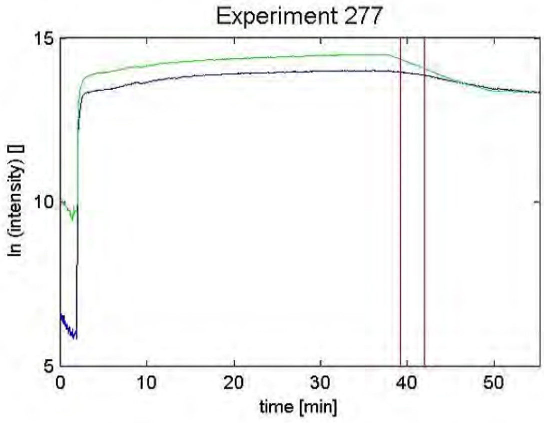

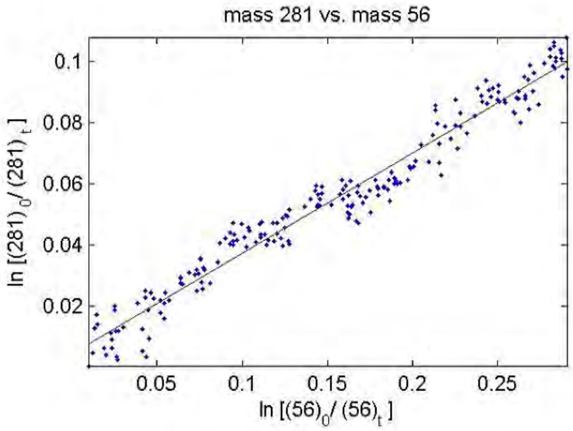

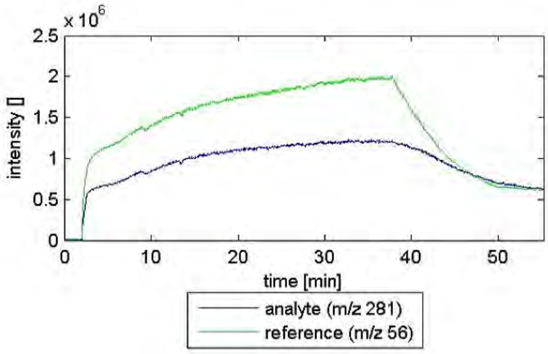

y= 0.004 + 0.328 * x
r= 0.980 n= 201
oven temperature: 80°C
analyte rate constant:
2.80e-12 cm^3 $molec^{-3}$ sec^{-1}
uncertainty range:
[2.55e-12;3.07e-12]
ln(rate)=-26.60
reference shift: 0.0 minutes

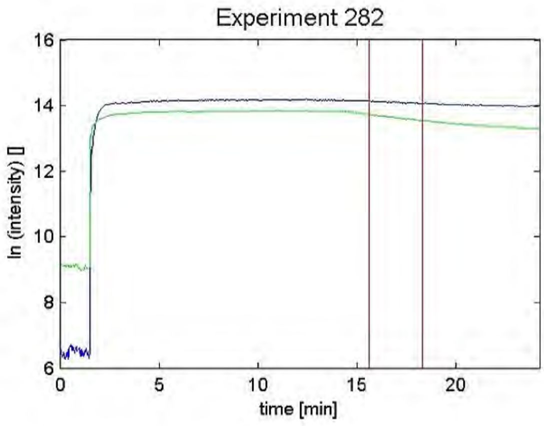

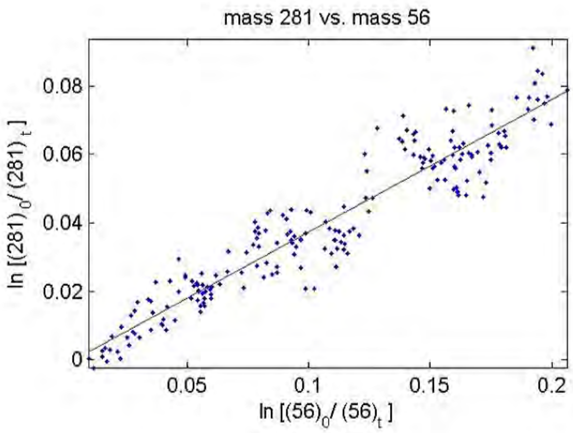

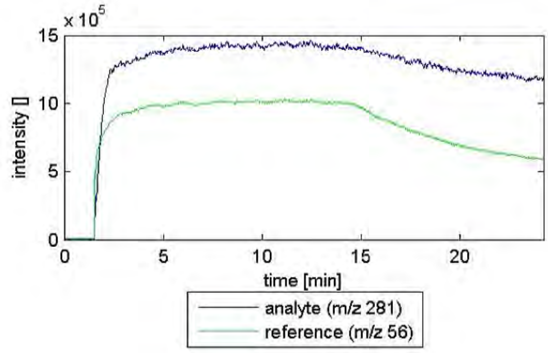

y= -0.001 + 0.387 * x
r= 0.947 n= 201
oven temperature: 80°C
analyte rate constant:
3.30e-12 cm^3 $molec^{-3}$ sec^{-1}
uncertainty range:
[3.01e-12;3.63e-12]
ln(rate)=-26.44
reference shift: 0.0 minutes

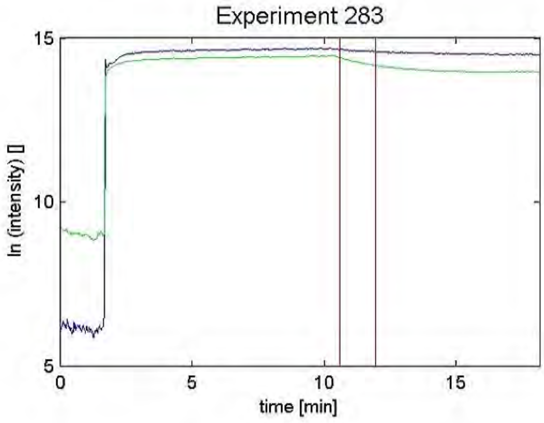

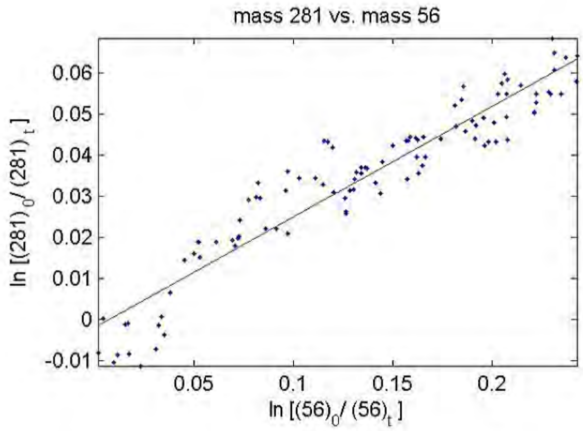

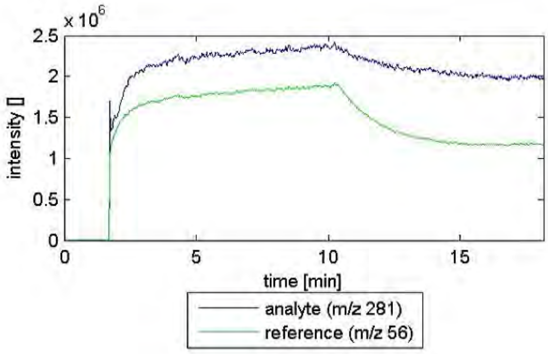

y= -0.002 + 0.270 * x
r= 0.947 n= 101
oven temperature: 80°C
analyte rate constant:
2.30e-12 cm^3 $molec^{-3}$ sec^{-1}
uncertainty range:
[2.10e-12;2.53e-12]
ln(rate)=-26.80
reference shift: 0.0 minutes

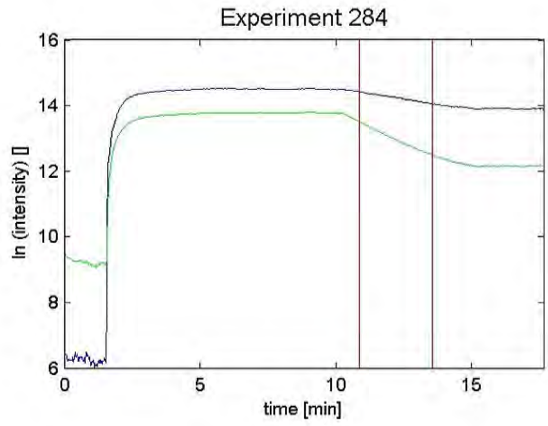

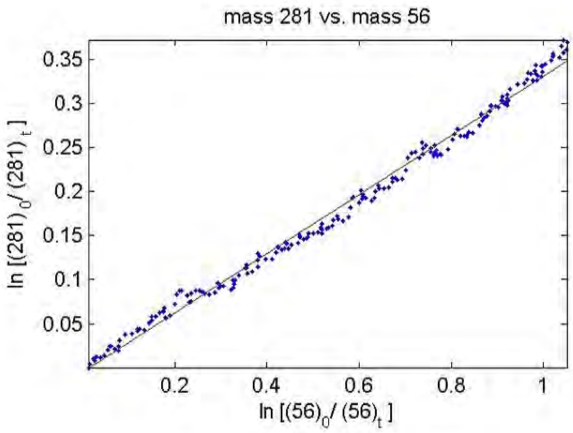

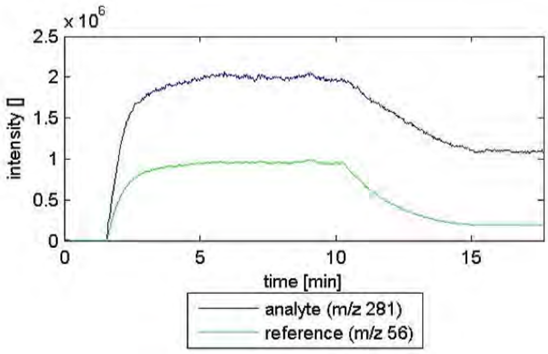

y= -0.005 + 0.335 * x
r= 0.996 n= 201
oven temperature: 80°C
analyte rate constant:
2.86e-12 cm^3 $molec^{-3}$ sec^{-1}
uncertainty range:
[2.61e-12;3.14e-12]
ln(rate)=-26.58
reference shift: 0.0 minutes

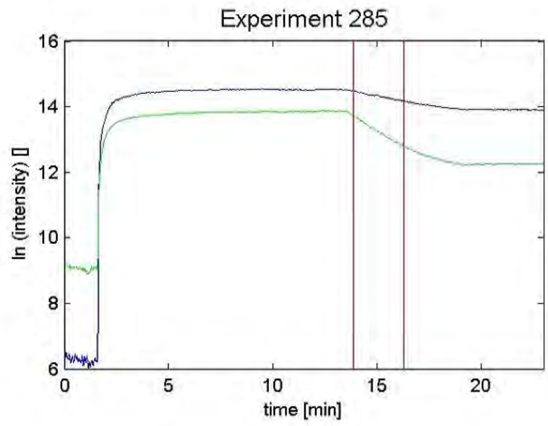

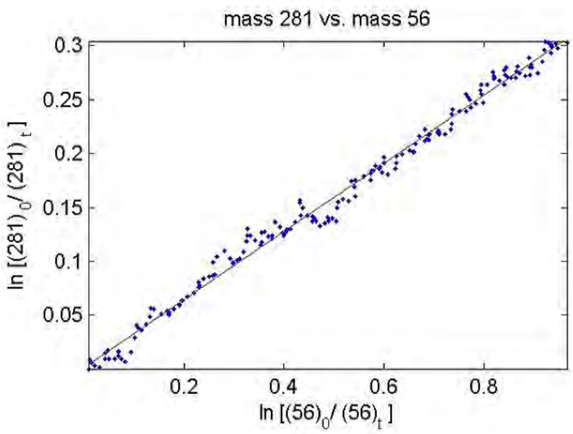

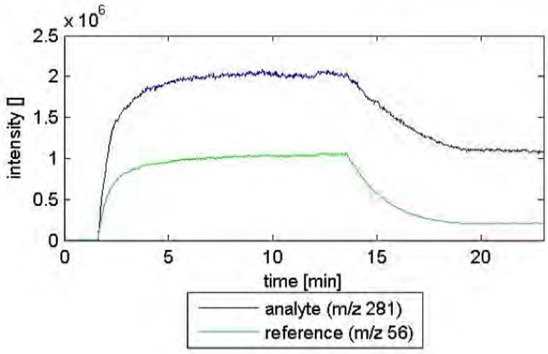

y= 0.001 + 0.316 * x
r= 0.995 n= 181
oven temperature: 80°C
analyte rate constant:
2.70e-12 cm^3 $molec^{-3}$ sec^{-1}
uncertainty range:
[2.45e-12;2.96e-12]
ln(rate)=-26.64
reference shift: 0.0 minutes

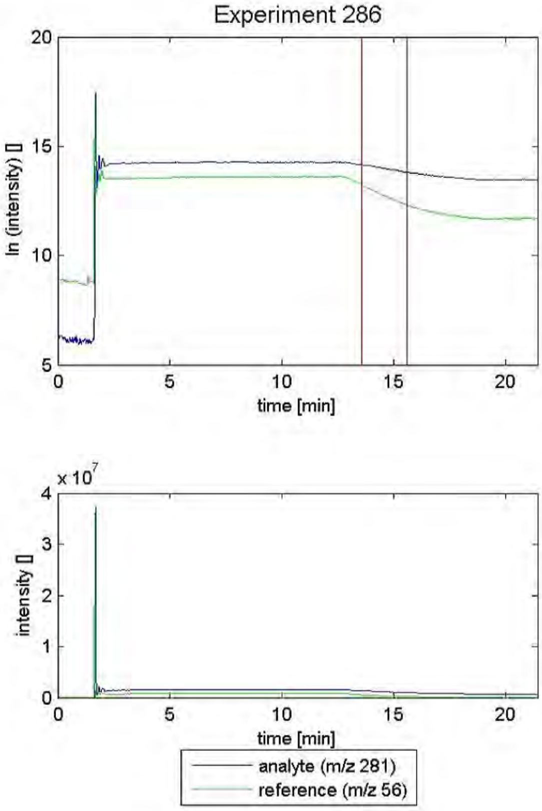

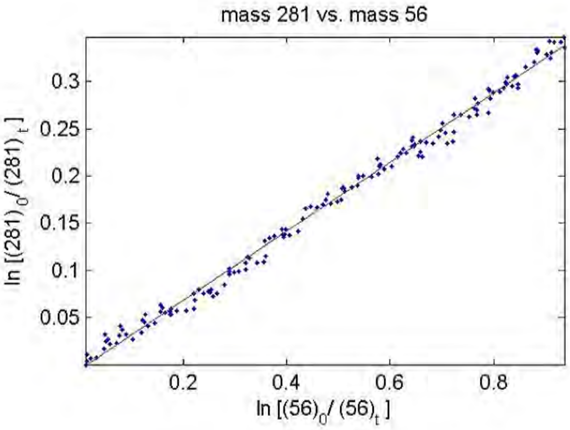

y= -0.005 + 0.366 * x
r= 0.997 n= 151
oven temperature: 80°C
analyte rate constant:
3.12e-12 cm^3 $molec^{-3}$ sec^{-1}
uncertainty range:
[2.84e-12;3.43e-12]
ln(rate)=-26.49
reference shift: 0.0 minutes

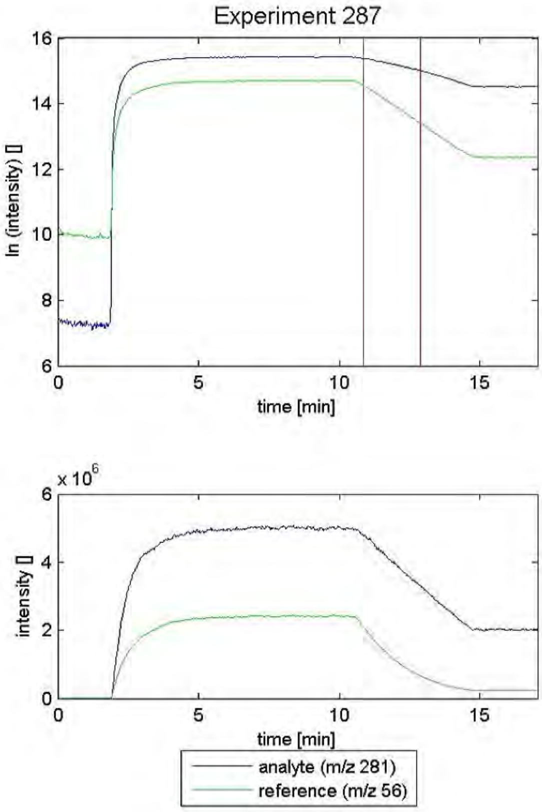

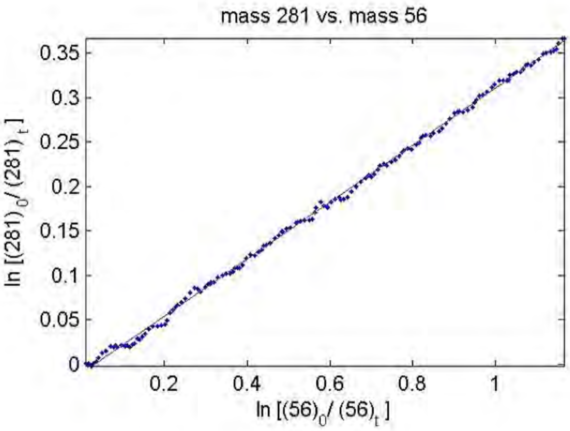

y= -0.011 + 0.321 * x
r= 0.999 n= 151
oven temperature: 60°C
analyte rate constant:
2.55e-12 cm^3 $molec^{-3}$ sec^{-1}
uncertainty range:
[2.31e-12;2.82e-12]
ln(rate)=-26.69
reference shift: 0.0 minutes

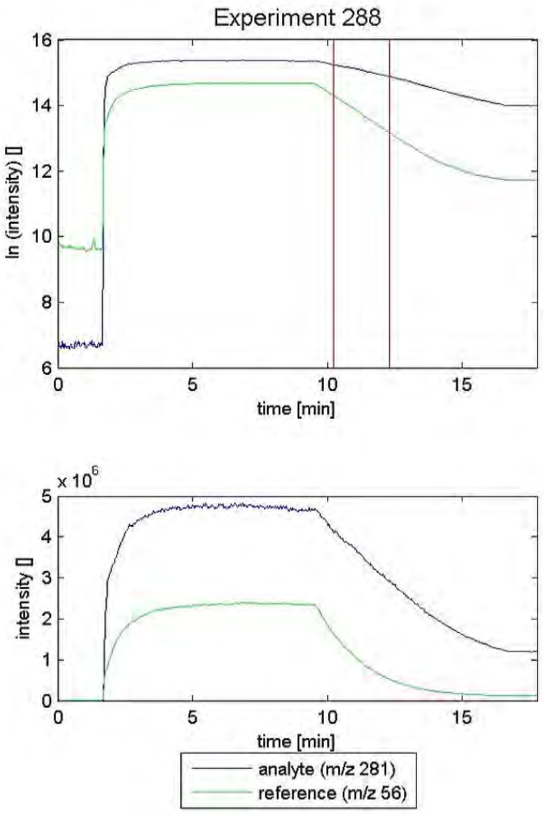

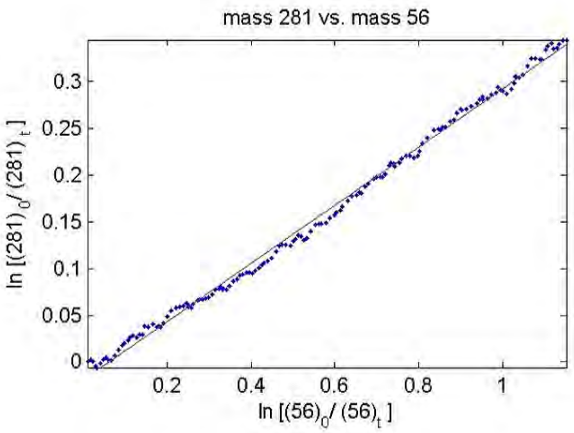

y= -0.019 + 0.311 * x
r= 0.998 n= 151
oven temperature: 60°C
analyte rate constant:
2.47e-12 cm^3 $molec^{-3}$ sec^{-1}
uncertainty range:
[2.24e-12;2.73e-12]
ln(rate)=-26.73
reference shift: 0.0 minutes

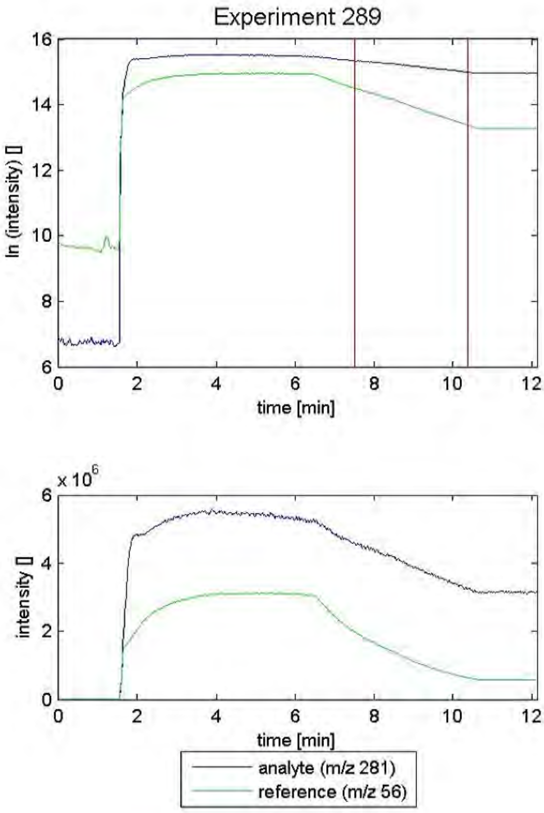

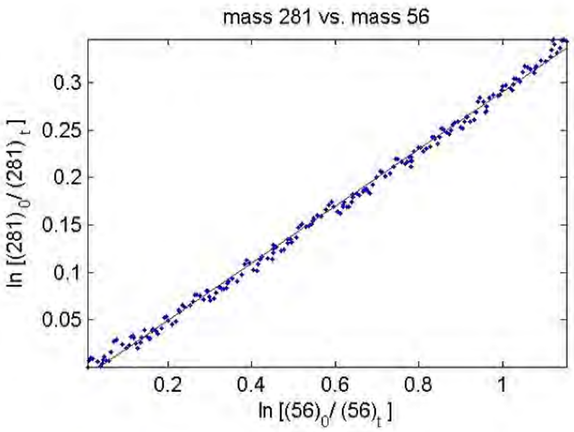

y= -0.011 + 0.301 * x
r= 0.998 n= 201
oven temperature: 50°C
analyte rate constant:
2.31e-12 cm^3 $molec^{-3}$ sec^{-1}
uncertainty range:
[2.08e-12;2.56e-12]
ln(rate)=-26.79
reference shift: 0.0 minutes

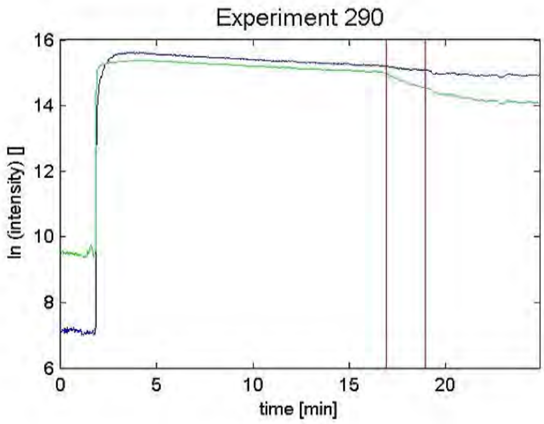

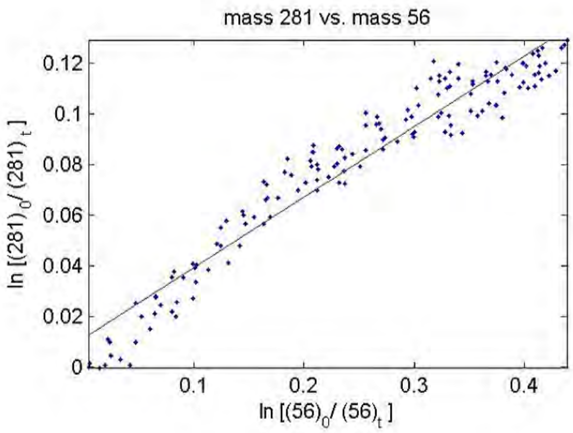

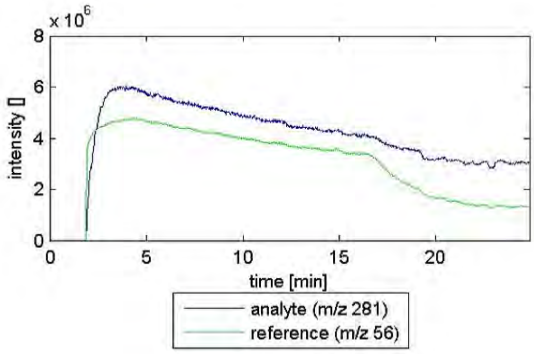

y= 0.012 + 0.278 * x
r= 0.965 n= 151
oven temperature: 50°C
analyte rate constant:
2.13e-12 cm^3 $molec^{-3}$ sec^{-1}
uncertainty range:
[1.92e-12;2.36e-12]
ln(rate)=-26.88
reference shift: 0.0 minutes

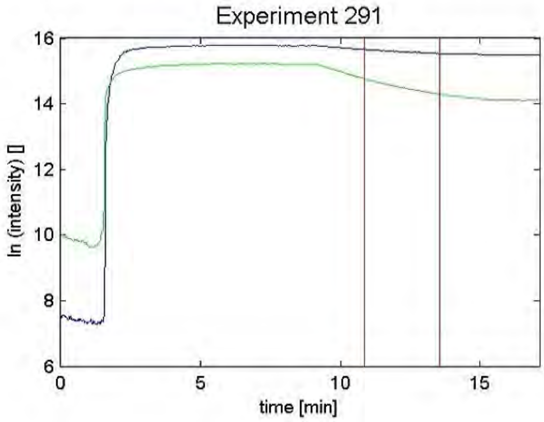

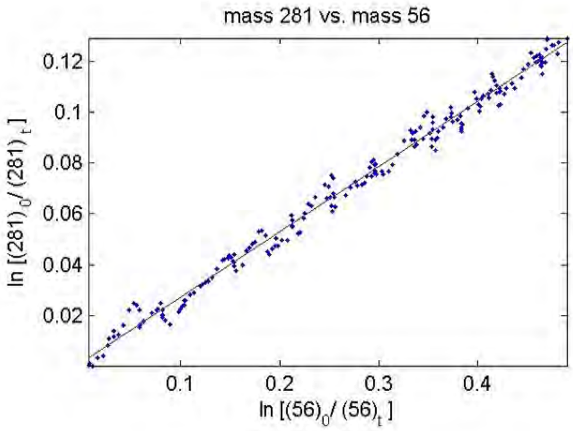

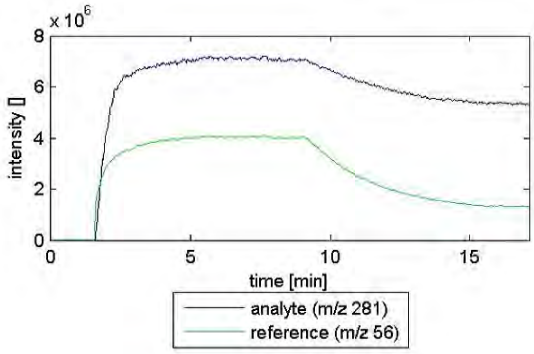

y= 0.002 + 0.257 * x
r= 0.995 n= 201
oven temperature: 50°C
analyte rate constant:
1.97e-12 cm^3 $molec^{-3}$ sec^{-1}
uncertainty range:
[1.78e-12;2.18e-12]
ln(rate)=-26.95
reference shift: 0.0 minutes

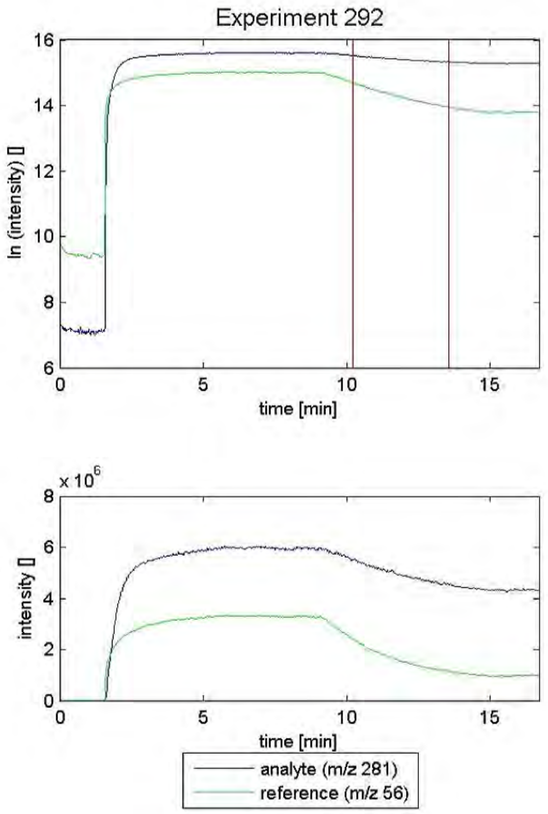

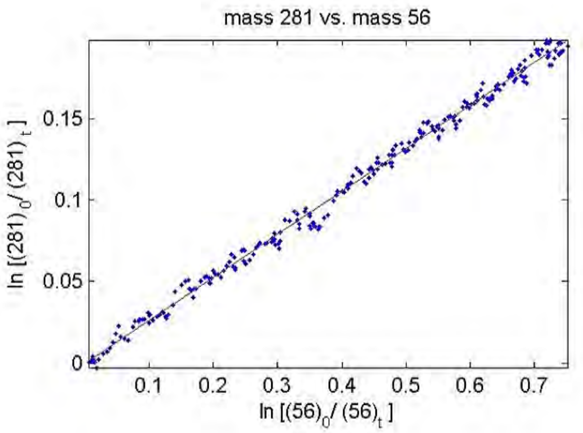

y= -0.000 + 0.264 * x
r= 0.997 n= 251
oven temperature: 50°C
analyte rate constant:
2.02e-12 cm^3 $molec^{-3}$ sec^{-1}
uncertainty range:
[1.83e-12;2.24e-12]
ln(rate)=-26.93
reference shift: 0.0 minutes

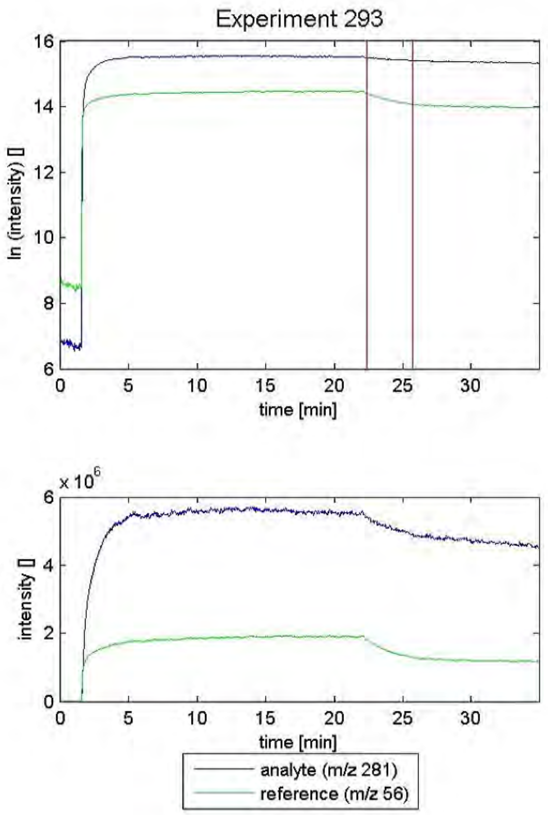

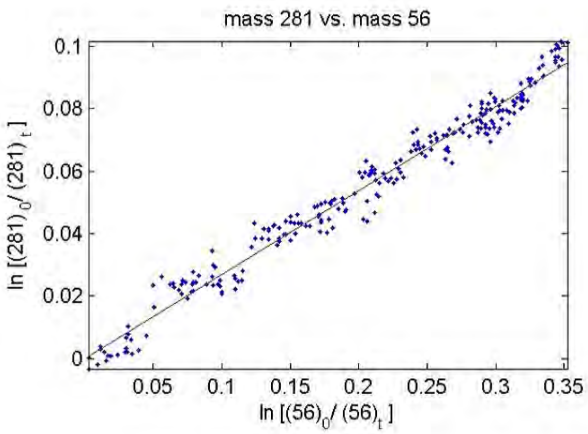

y= 0.000 + 0.268 * x
r= 0.987 n= 251
oven temperature: 70°C
analyte rate constant:
2.21e-12 cm^3 $molec^{-3}$ sec^{-1}
uncertainty range:
[2.01e-12;2.43e-12]
ln(rate)=-26.84
reference shift: 0.0 minutes

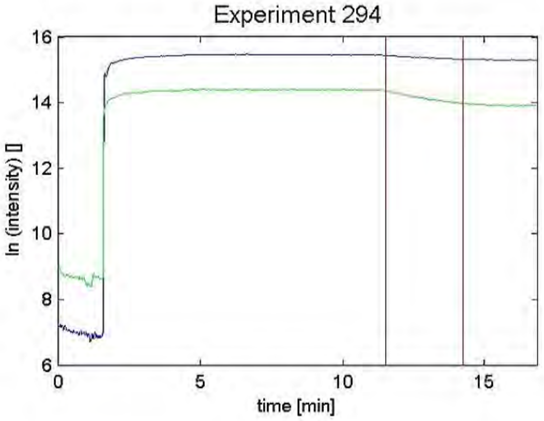

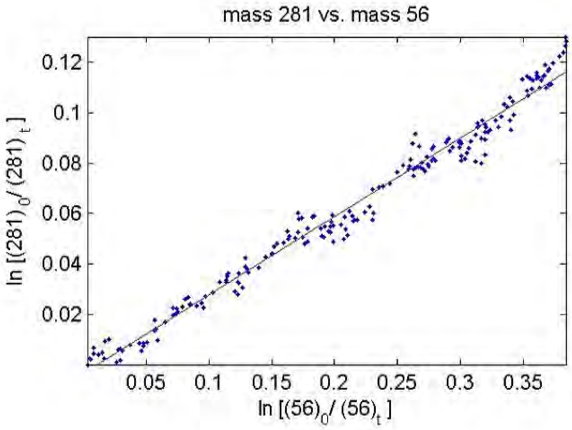

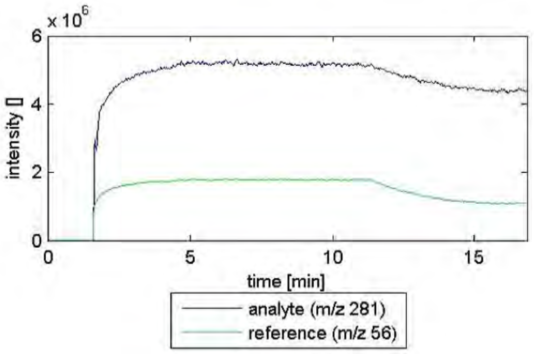

y= -0.003 + 0.310 * x
r= 0.990 n= 201
oven temperature: 70°C
analyte rate constant:
2.55e-12 cm^3 molec^{-3} sec^{-1}
uncertainty range:
[2.32e-12;2.81e-12]
ln(rate)=-26.69
reference shift: 0.0 minutes

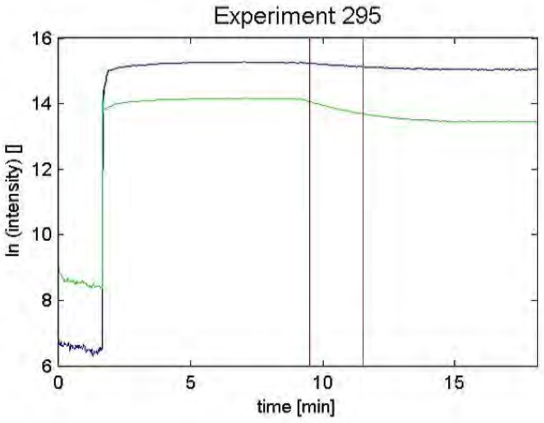

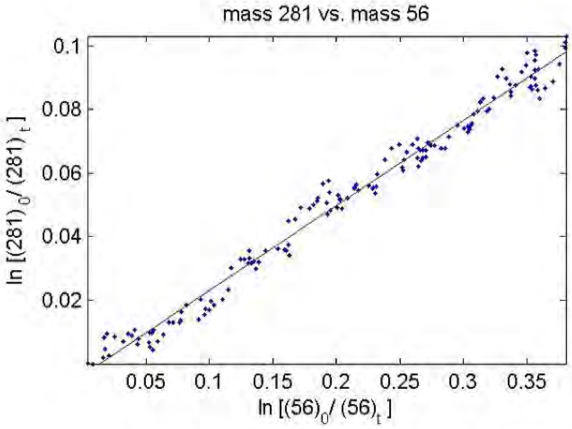

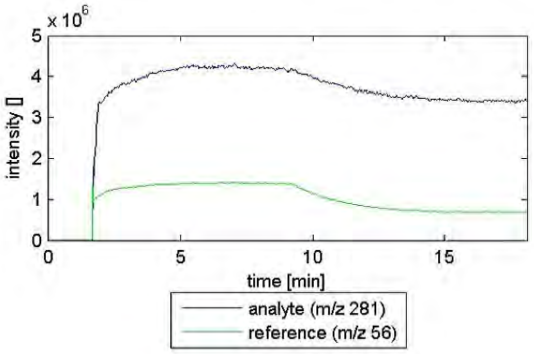

y= -0.004 + 0.267 * x
r= 0.992 n= 151
oven temperature: 70°C
analyte rate constant:
2.20e-12 cm^3 molec^{-3} sec^{-1}
uncertainty range:
[1.99e-12;2.42e-12]
ln(rate)=-26.84
reference shift: 0.0 minutes

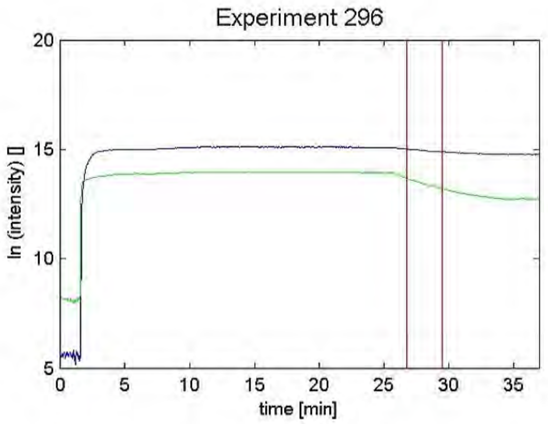

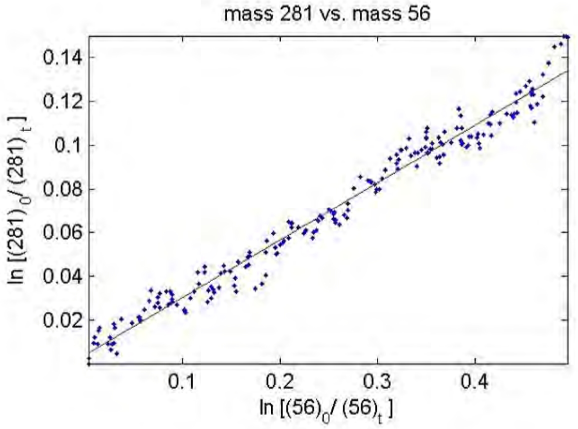

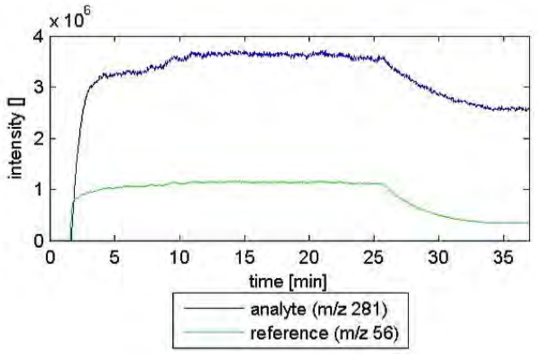

y= 0.004 + 0.263 * x
r= 0.987 n= 201
oven temperature: 40°C
analyte rate constant:
1.94e-12 cm^3 $molec^{-3}$ sec^{-1}
uncertainty range:
[1.75e-12;2.16e-12]
ln(rate)=-26.97
reference shift: 0.0 minutes

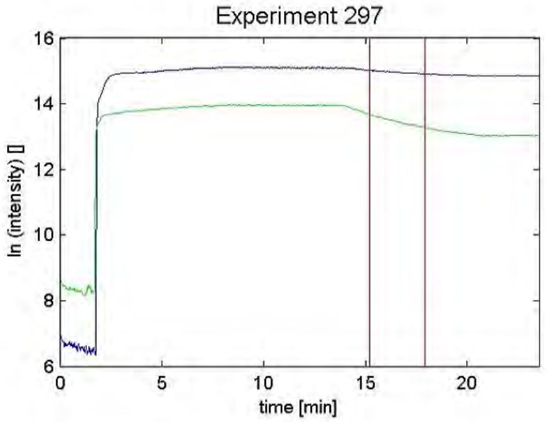

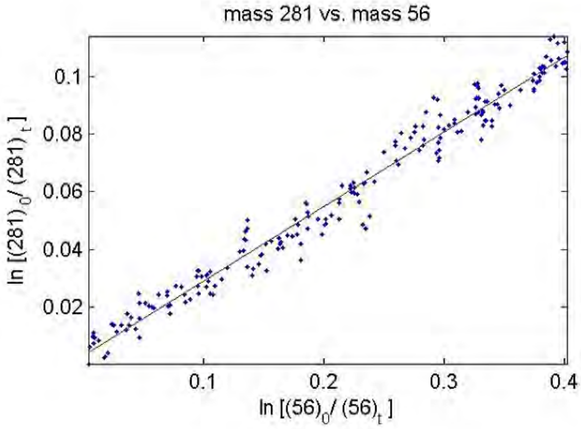

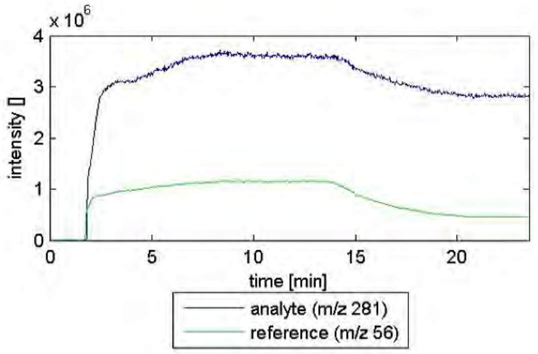

y= 0.003 + 0.259 * x
r= 0.987 n= 201
oven temperature: 40°C
analyte rate constant:
1.91e-12 cm^3 $molec^{-3}$ sec^{-1}
uncertainty range:
[1.72e-12;2.12e-12]
ln(rate)=-26.98
reference shift: 0.0 minutes

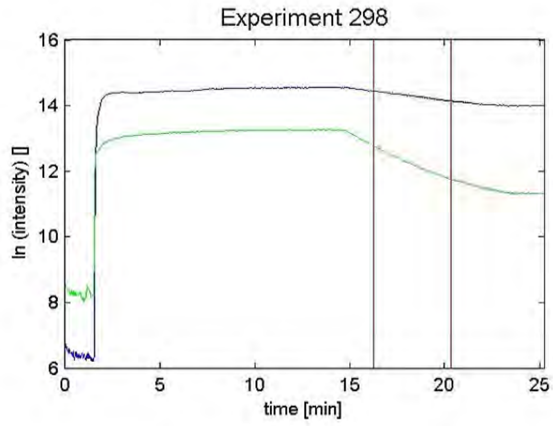

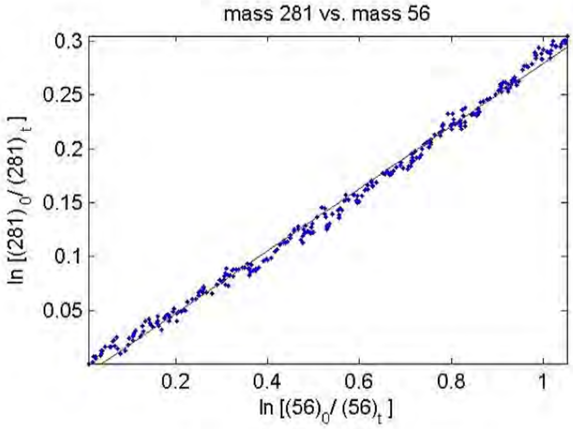

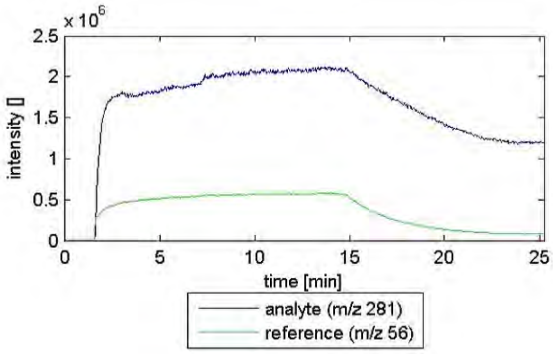

y= -0.011 + 0.291 * x
r= 0.997 n= 301
oven temperature: 40°C
analyte rate constant:
2.15e-12 cm^3 $molec^{-3}$ sec^{-1}
uncertainty range:
[1.93e-12;2.38e-12]
ln(rate)=-26.87
reference shift: 0.0 minutes

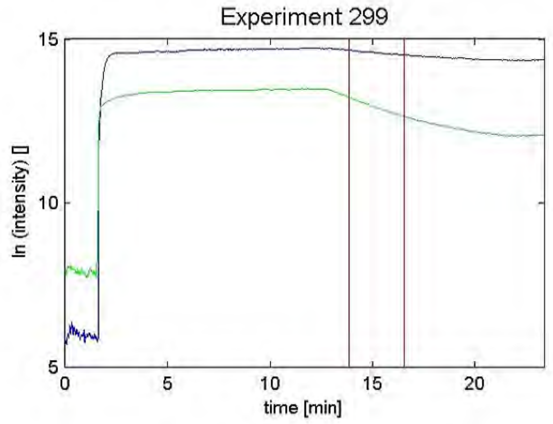

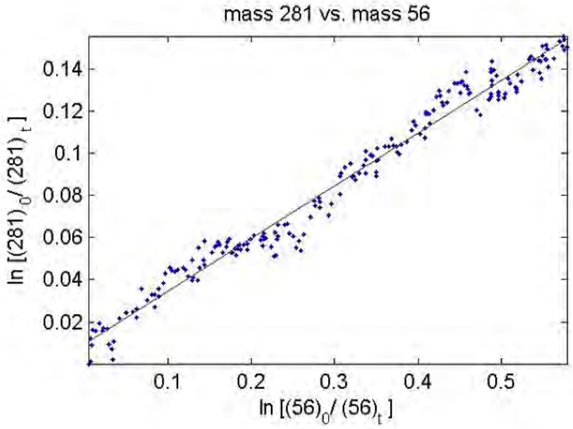

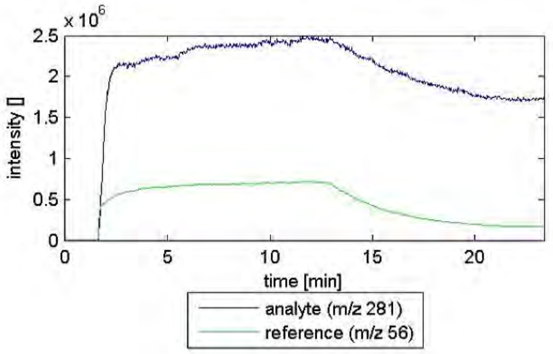

y= 0.010 + 0.250 * x
r= 0.988 n= 201
oven temperature: 40°C
analyte rate constant:
1.84e-12 cm^3 $molec^{-3}$ sec^{-1}
uncertainty range:
[1.66e-12;2.05e-12]
ln(rate)=-27.02
reference shift: 0.0 minutes

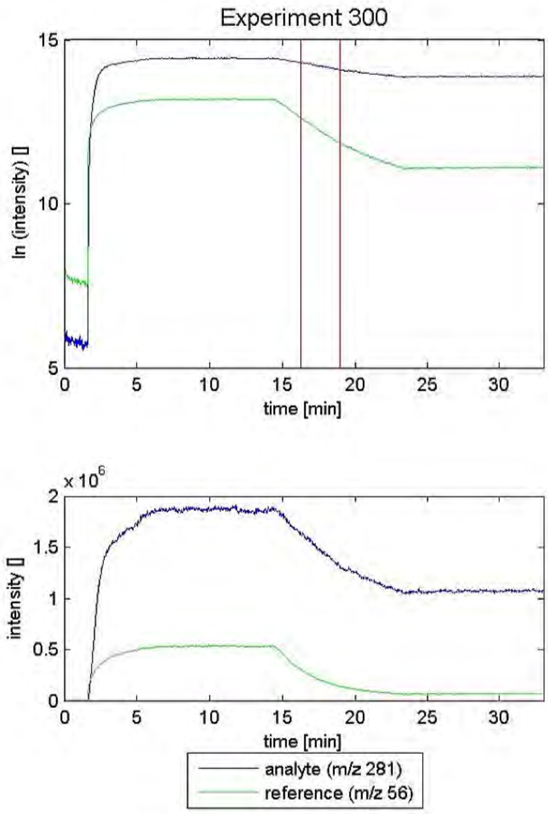

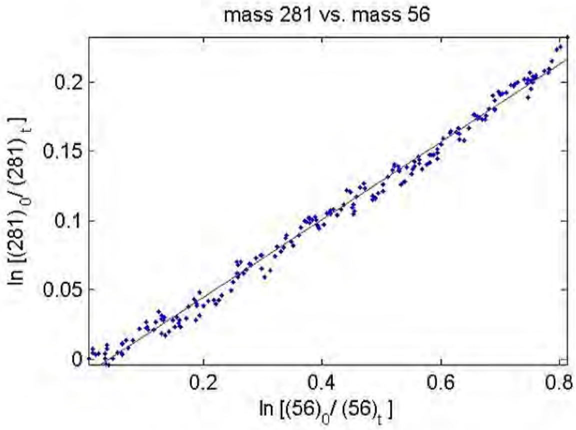

y= -0.012 + 0.281 * x
r= 0.995 n= 201
oven temperature: 40°C
analyte rate constant:
2.07e-12 cm^3 $molec^{-3}$ sec^{-1}
uncertainty range:
[1.87e-12;2.30e-12]
ln(rate)=-26.90
reference shift: 0.0 minutes

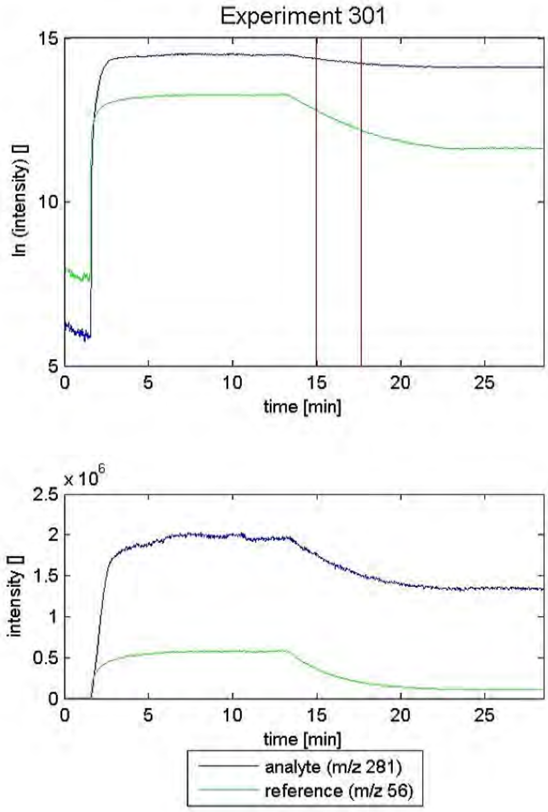

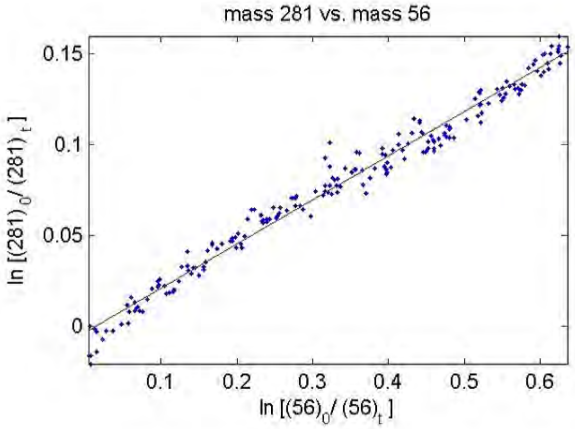

y= -0.003 + 0.242 * x
r= 0.989 n= 201
oven temperature: 40°C
analyte rate constant:
1.79e-12 cm^3 $molec^{-3}$ sec^{-1}
uncertainty range:
[1.61e-12;1.99e-12]
ln(rate)=-27.05
reference shift: 0.0 minutes

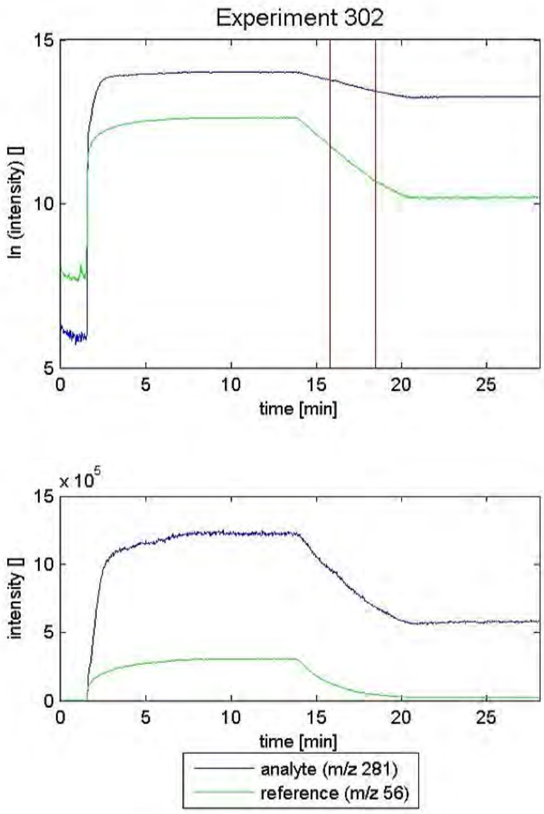

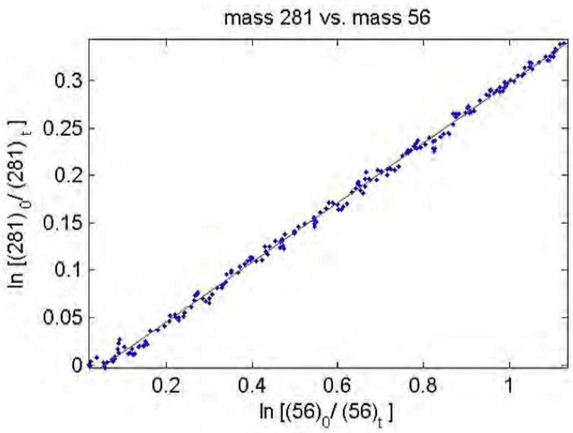

y= -0.018 + 0.315 * x
r= 0.999 n= 201
oven temperature: 40°C
analyte rate constant:
2.32e-12 cm^3 $molec^{-3}$ sec^{-1}
uncertainty range:
[2.09e-12;2.58e-12]
ln(rate)=-26.79
reference shift: 0.0 minutes

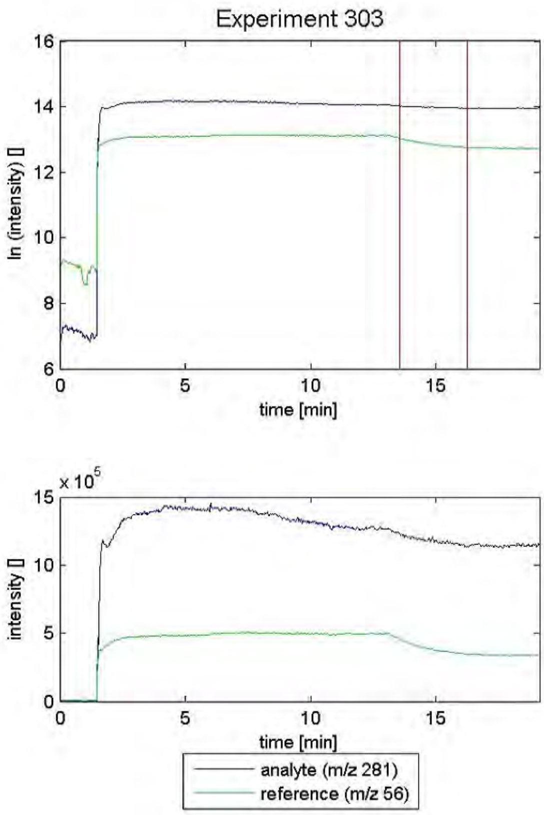

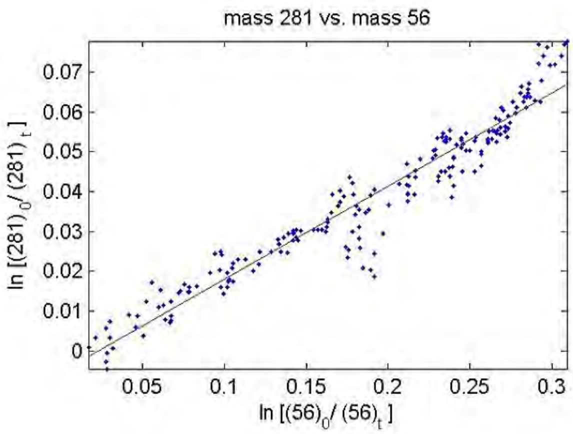

y= -0.005 + 0.234 * x
r= 0.964 n= 201
oven temperature: 80°C
analyte rate constant:
2.00e-12 cm^3 $molec^{-3}$ sec^{-1}
uncertainty range:
[1.82e-12;2.19e-12]
ln(rate)=-26.94
reference shift: 0.0 minutes

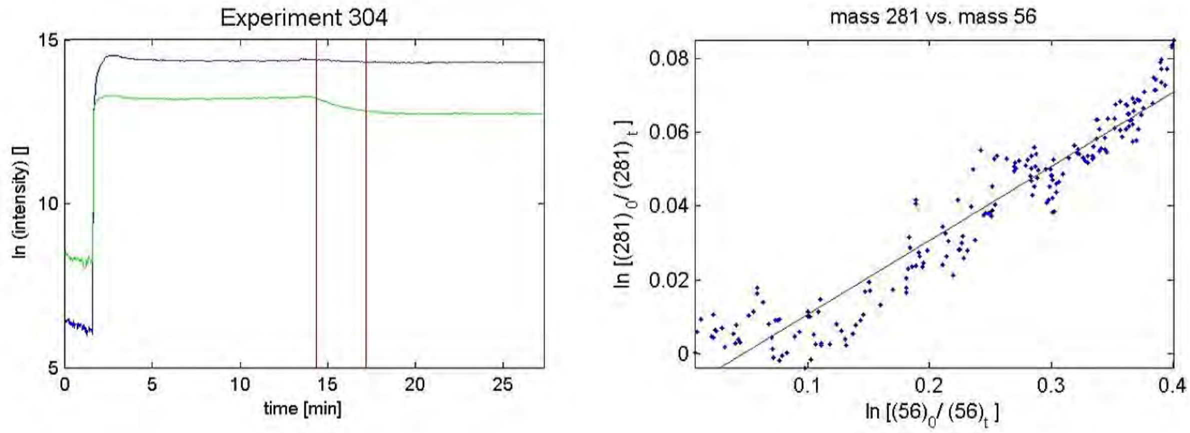

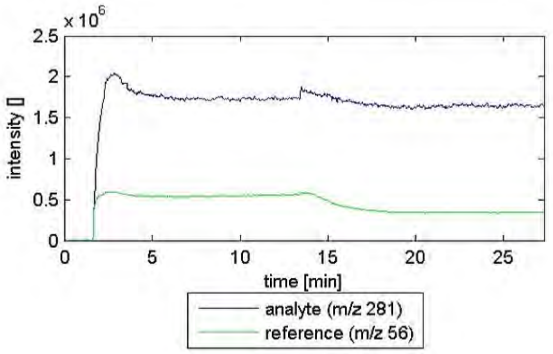

y= -0.009 + 0.200 * x
r= 0.957 n= 201
oven temperature: 80°C
analyte rate constant:
1.71e-12 cm^3 $molec^{-3}$ sec^{-1}
uncertainty range:
[1.56e-12;1.87e-12]
ln(rate)=-27.10
reference shift: 0.0 minutes

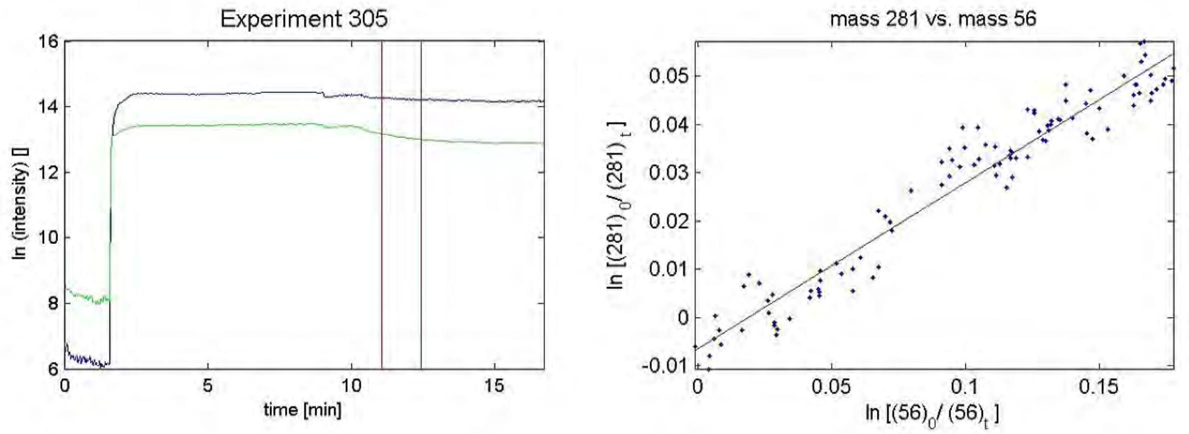

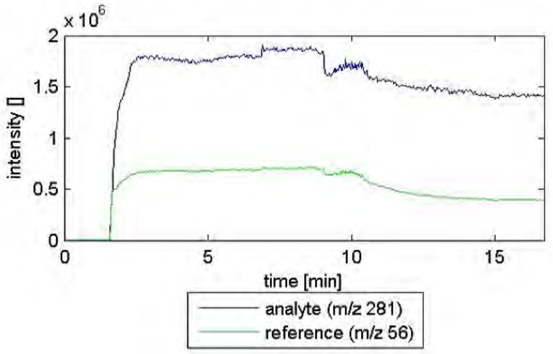

y= -0.006 + 0.344 * x
r= 0.970 n= 101
oven temperature: 80°C
analyte rate constant:
2.93e-12 cm^3 $molec^{-3}$ sec^{-1}
uncertainty range:
[2.67e-12;3.22e-12]
ln(rate)=-26.55
reference shift: 0.0 minutes

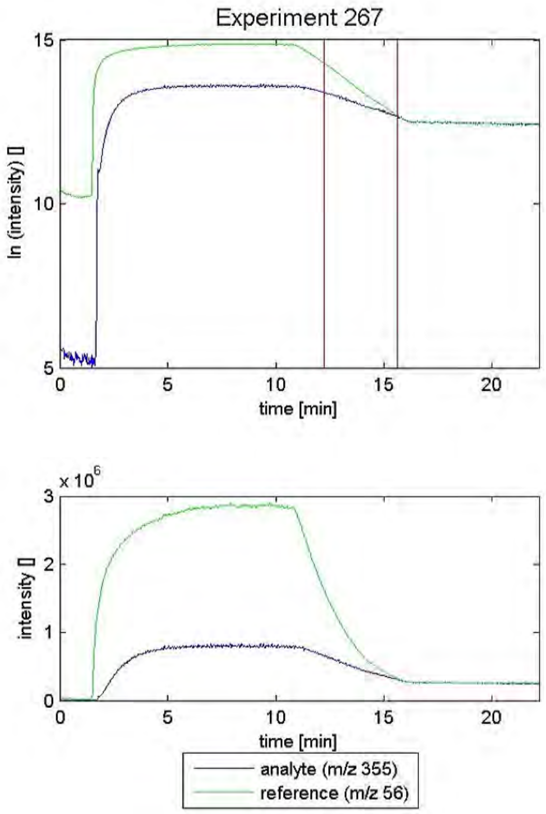

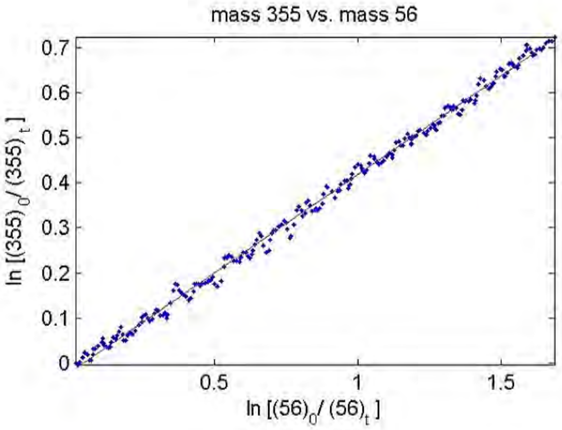

y= -0.020 + 0.439 * x
r= 0.998 n= 251
oven temperature: 40°C
analyte rate constant:
3.24e-12 cm^3 $molec^{-3}$ sec^{-1}
uncertainty range:
[2.92e-12;3.60e-12]
ln(rate)=-26.46
reference shift: 0.0 minutes

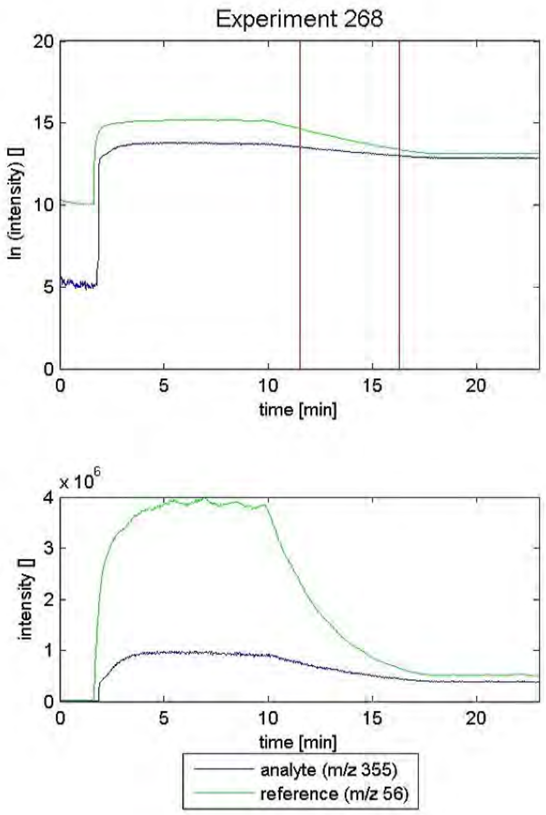

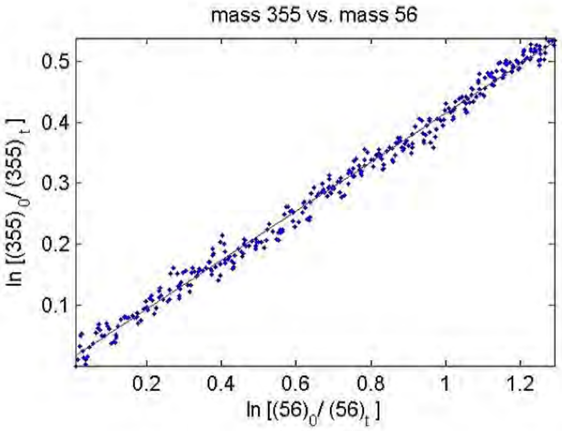

y= 0.012 + 0.403 * x
r= 0.996 n= 351
oven temperature: 40°C
analyte rate constant:
2.97e-12 cm^3 $molec^{-3}$ sec^{-1}
uncertainty range:
[2.68e-12;3.30e-12]
ln(rate)=-26.54
reference shift: 0.0 minutes

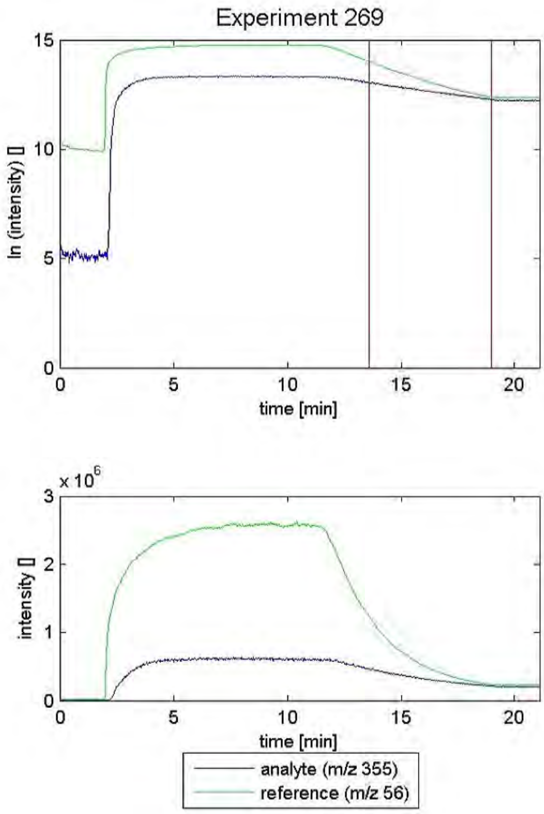

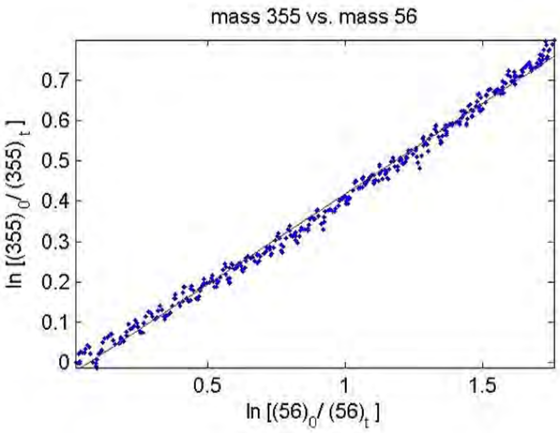

y= -0.030 + 0.448 * x
r= 0.997 n= 401
oven temperature: 40°C
analyte rate constant:
3.30e-12 cm^3 $molec^{-3}$ sec^{-1}
uncertainty range:
[2.97e-12;3.67e-12]
ln(rate)=-26.44
reference shift: 0.0 minutes

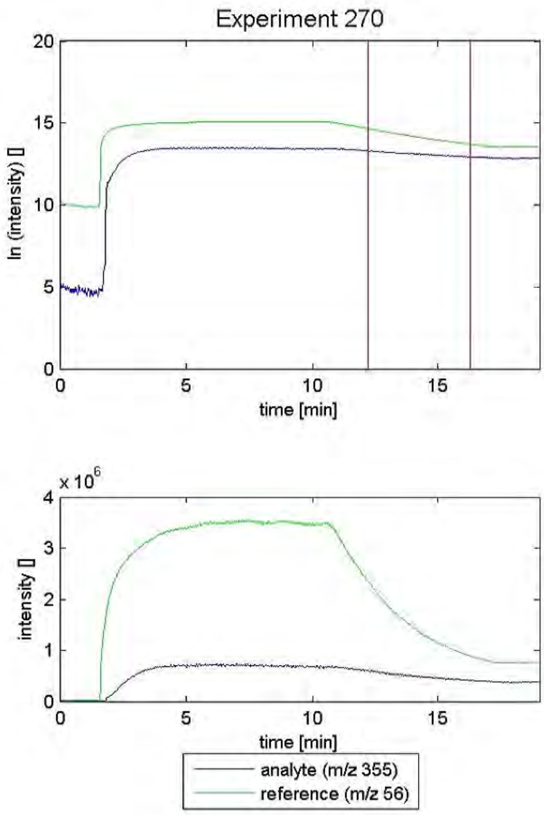

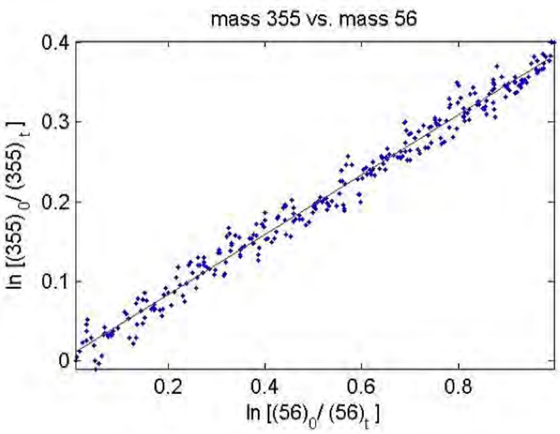

y= 0.008 + 0.376 * x
r= 0.991 n= 301
oven temperature: 40°C
analyte rate constant:
2.78e-12 cm^3 $molec^{-3}$ sec^{-1}
uncertainty range:
[2.50e-12;3.08e-12]
ln(rate)=-26.61
reference shift: 0.0 minutes

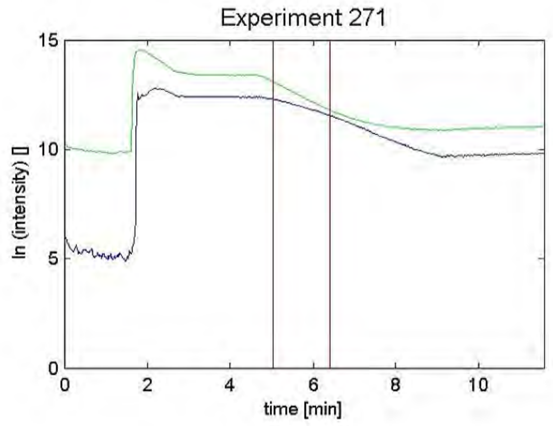

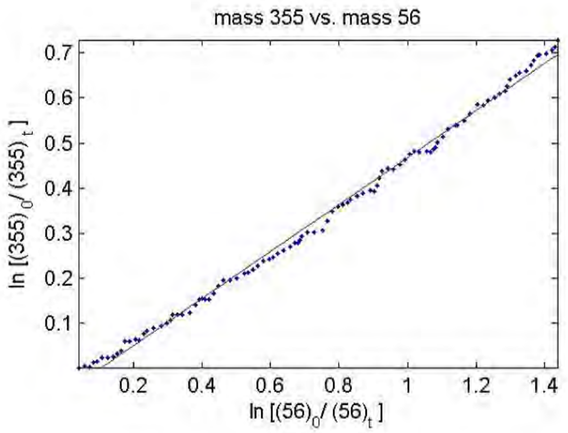

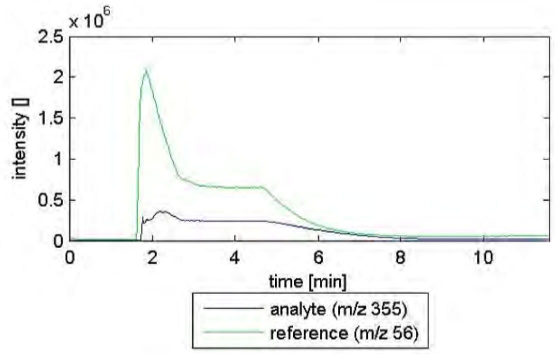

y= -0.055 + 0.523 * x
r= 0.998 n= 101
oven temperature: 60°C
analyte rate constant:
4.15e-12 cm^3 molec^{-3} sec^{-1}
uncertainty range:
[3.76e-12;4.59e-12]
ln(rate)=-26.21
reference shift: 0.0 minutes

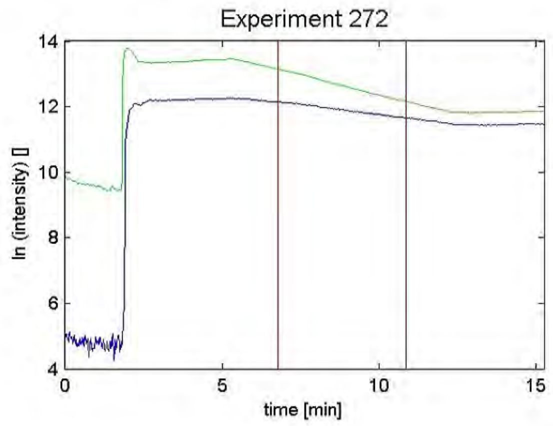

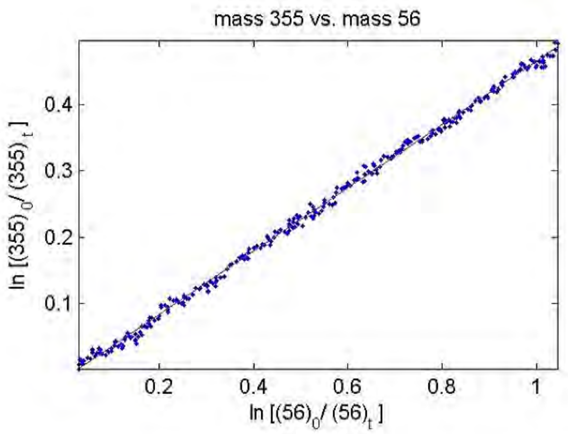

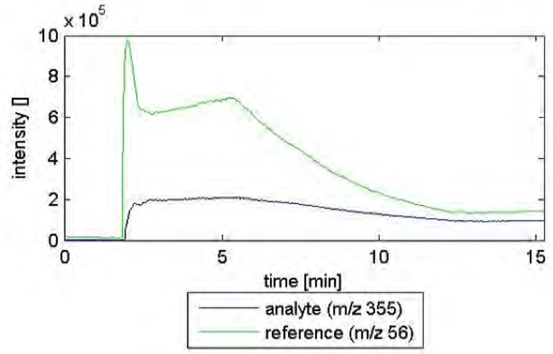

y= -0.013 + 0.478 * x
r= 0.999 n= 301
oven temperature: 60°C
analyte rate constant:
3.80e-12 cm^3 molec^{-3} sec^{-1}
uncertainty range:
[3.44e-12;4.19e-12]
ln(rate)=-26.30
reference shift: 0.0 minutes

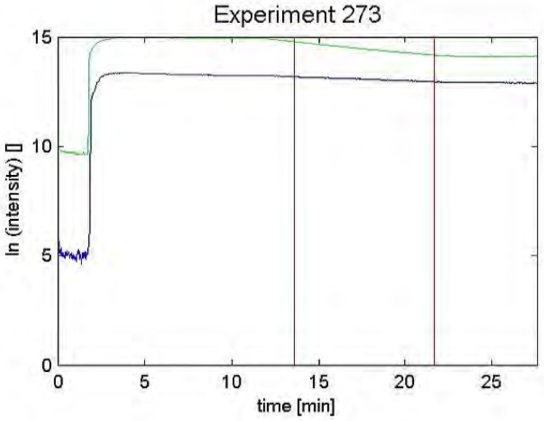

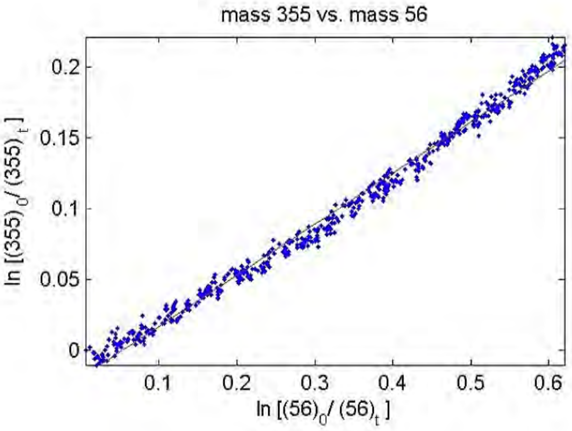

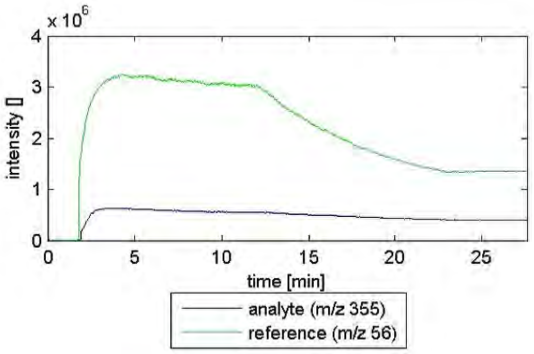

y= -0.019 + 0.361 * x
r= 0.995 n= 601
oven temperature: 60°C
analyte rate constant:
2.87e-12 cm^3 molec^{-3} sec^{-1}
uncertainty range:
[2.60e-12;3.17e-12]
ln(rate)=-26.58
reference shift: 0.0 minutes

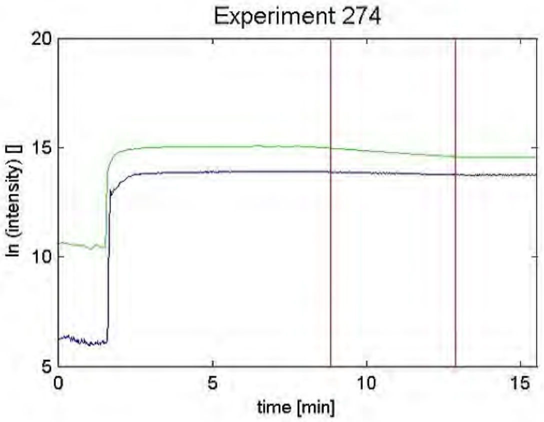

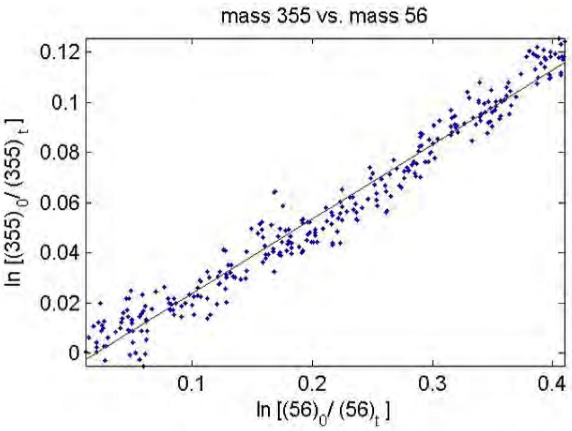

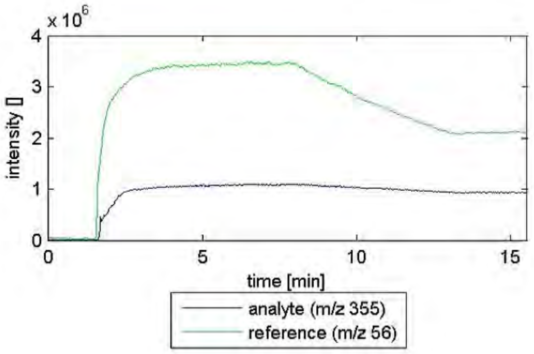

y= -0.006 + 0.297 * x
r= 0.981 n= 301
oven temperature: 60°C
analyte rate constant:
2.36e-12 cm^3 molec^{-3} sec^{-1}
uncertainty range:
[2.13e-12;2.60e-12]
ln(rate)=-26.77
reference shift: 0.0 minutes

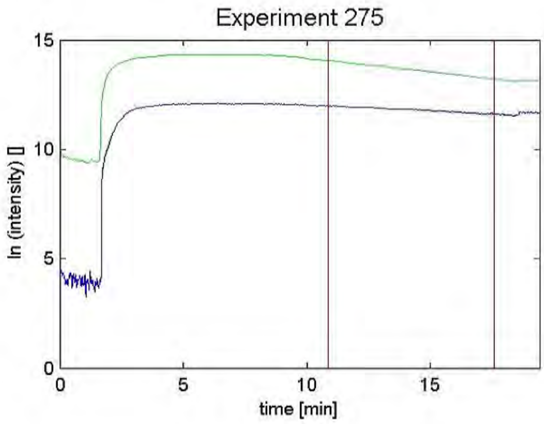

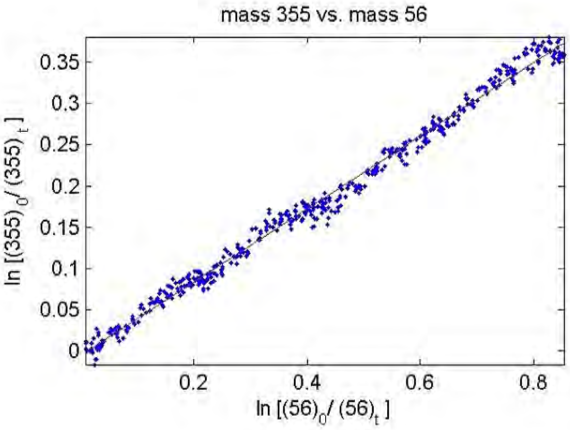

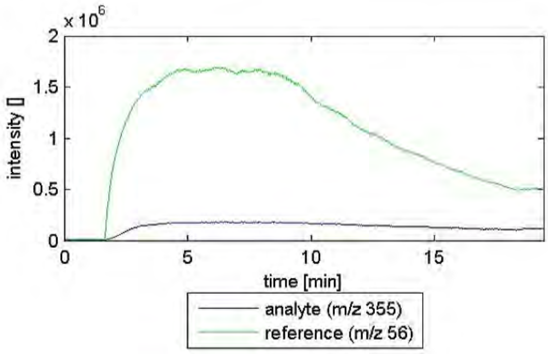

y= -0.005 + 0.443 * x
r= 0.996 n= 501
oven temperature: 60°C
analyte rate constant:
3.52e-12 cm^3 $molec^{-3}$ sec^{-1}
uncertainty range:
[3.19e-12;3.88e-12]
ln(rate)=-26.37
reference shift: 0.0 minutes

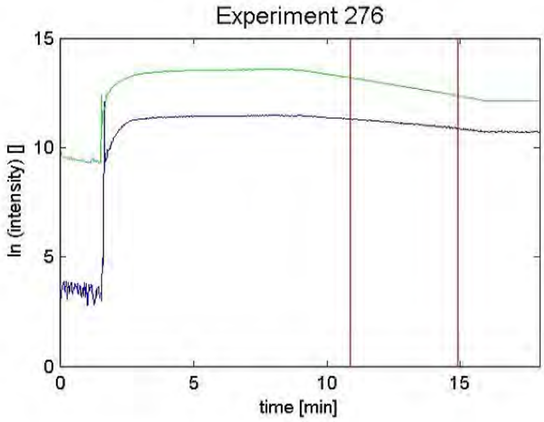

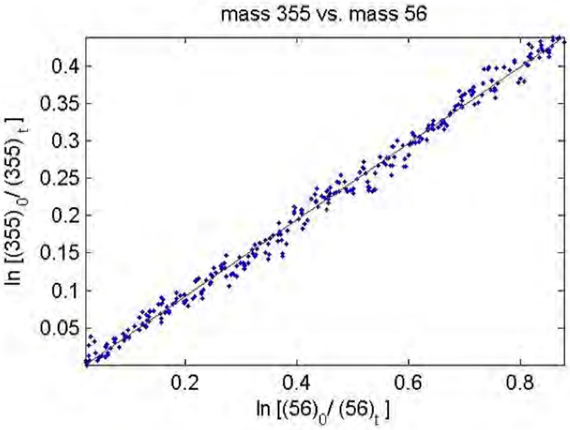

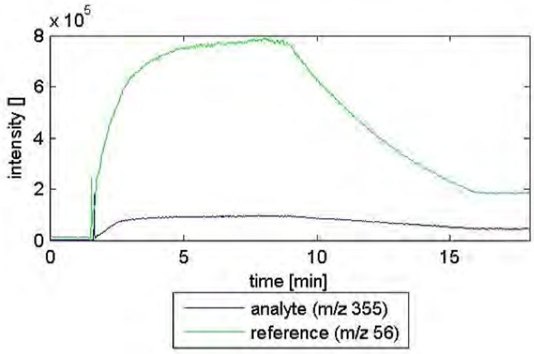

y= -0.010 + 0.511 * x
r= 0.996 n= 301
oven temperature: 60°C
analyte rate constant:
4.06e-12 cm^3 $molec^{-3}$ sec^{-1}
uncertainty range:
[3.67e-12;4.48e-12]
ln(rate)=-26.23
reference shift: 0.0 minutes

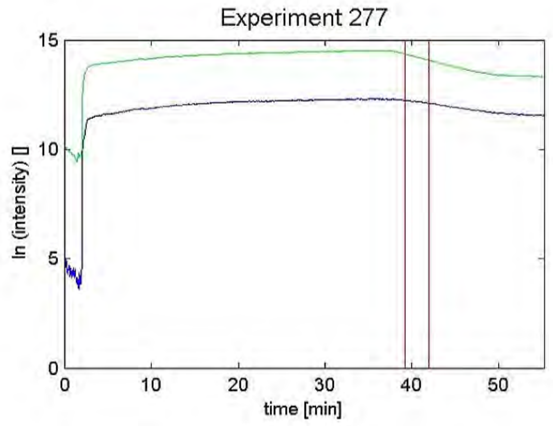

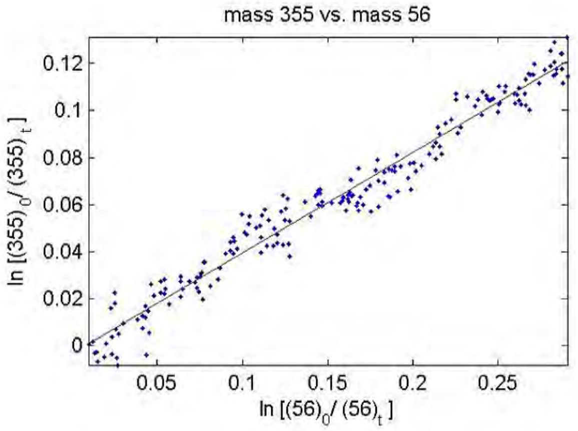

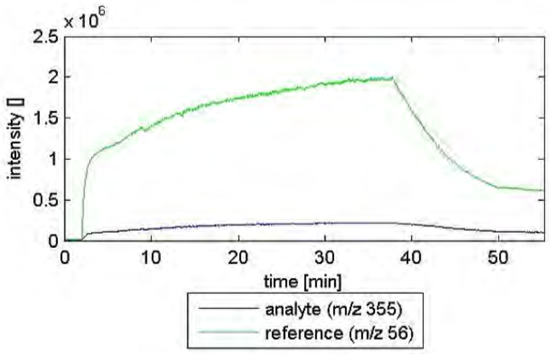

y= -0.003 + 0.428 * x
r= 0.982 n= 201
oven temperature: 80°C
analyte rate constant:
3.65e-12 cm^3 $molec^{-3}$ sec^{-1}
uncertainty range:
[3.33e-12;4.01e-12]
ln(rate)=-26.34
reference shift: 0.0 minutes

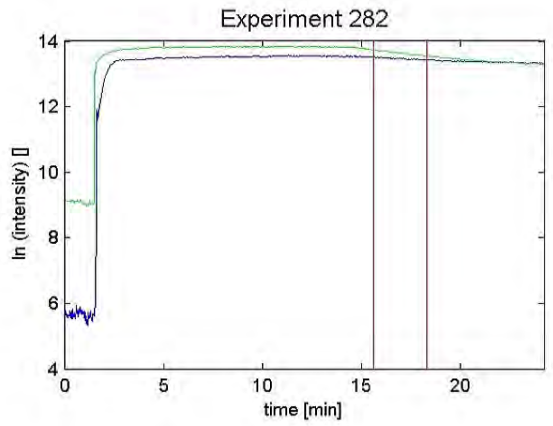

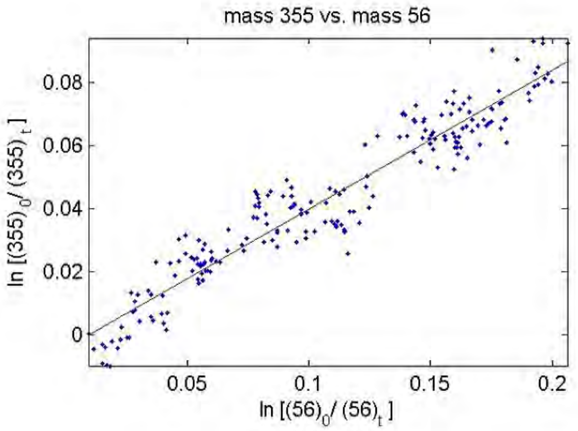

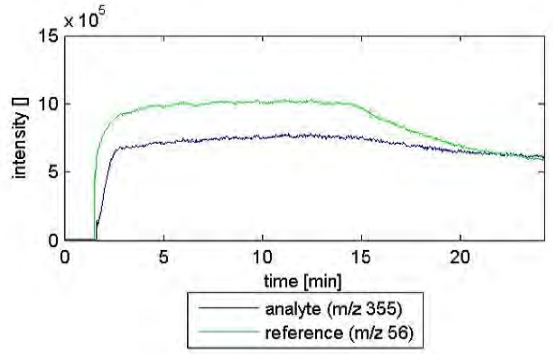

y= -0.004 + 0.440 * x
r= 0.955 n= 201
oven temperature: 80°C
analyte rate constant:
3.76e-12 cm^3 $molec^{-3}$ sec^{-1}
uncertainty range:
[3.42e-12;4.12e-12]
ln(rate)=-26.31
reference shift: 0.0 minutes

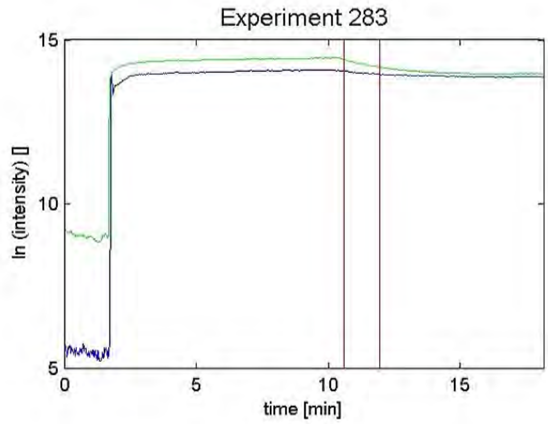

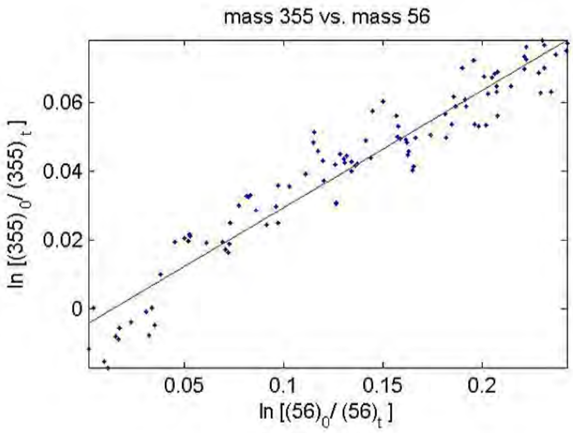

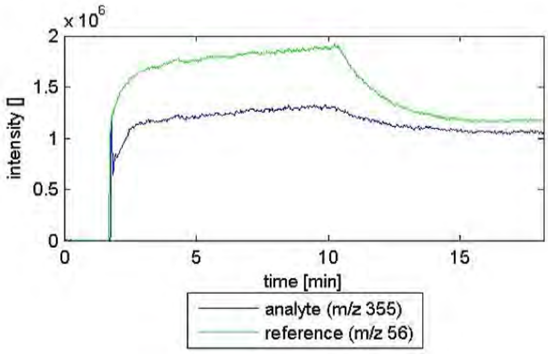

y= -0.005 + 0.341 * x
r= 0.959 n= 101
oven temperature: 80°C
analyte rate constant:
2.91e-12 cm^3 $molec^{-3}$ sec^{-1}
uncertainty range:
[2.65e-12;3.19e-12]
ln(rate)=-26.56
reference shift: 0.0 minutes

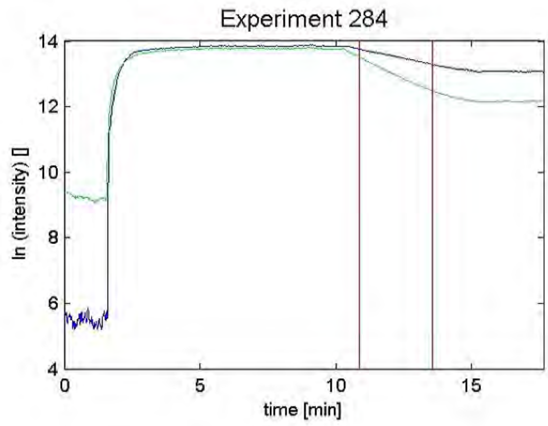

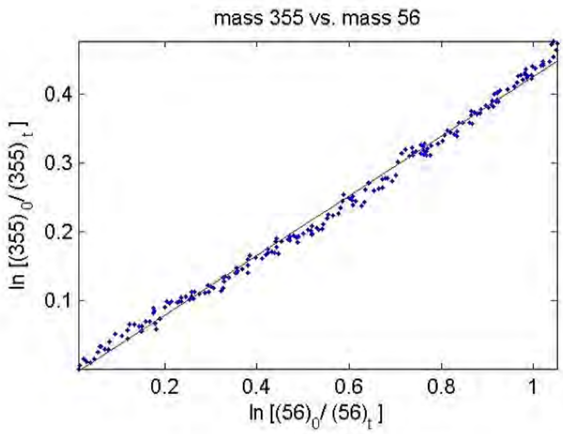

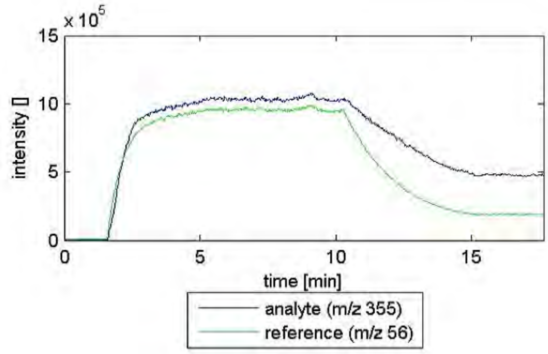

y= -0.009 + 0.434 * x
r= 0.997 n= 201
oven temperature: 80°C
analyte rate constant:
3.71e-12 cm^3 $molec^{-3}$ sec^{-1}
uncertainty range:
[3.38e-12;4.07e-12]
ln(rate)=-26.32
reference shift: 0.0 minutes

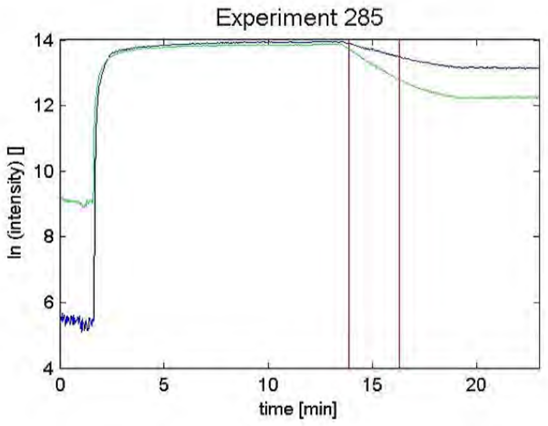

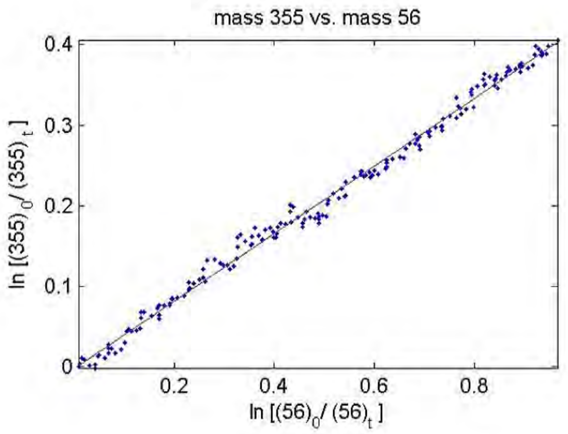

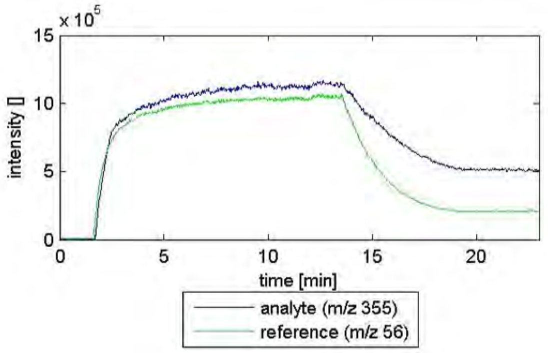

y= -0.003 + 0.420 * x
r= 0.997 n= 181
oven temperature: 80°C
analyte rate constant:
3.58e-12 cm^3 $molec^{-3}$ sec^{-1}
uncertainty range:
[3.26e-12;3.94e-12]
ln(rate)=-26.35
reference shift: 0.0 minutes

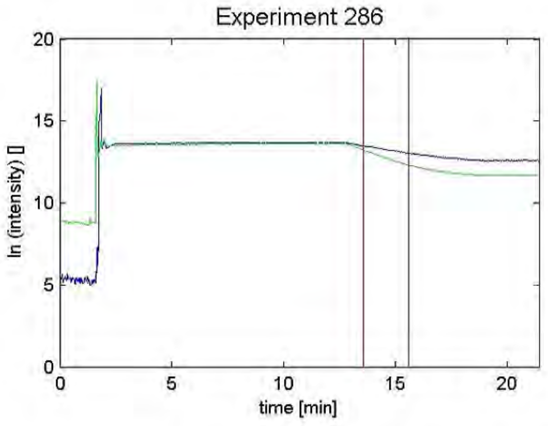

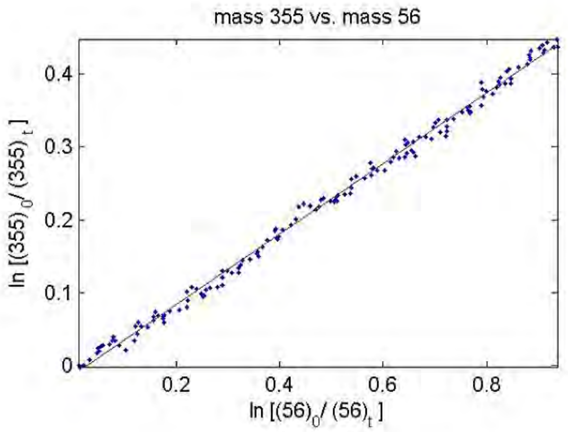

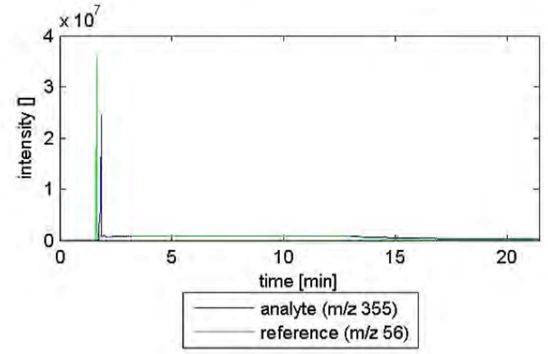

y= -0.013 + 0.483 * x
r= 0.998 n= 151
oven temperature: 80°C
analyte rate constant:
4.13e-12 cm^3 $molec^{-3}$ sec^{-1}
uncertainty range:
[3.76e-12;4.53e-12]
ln(rate)=-26.21
reference shift: 0.0 minutes

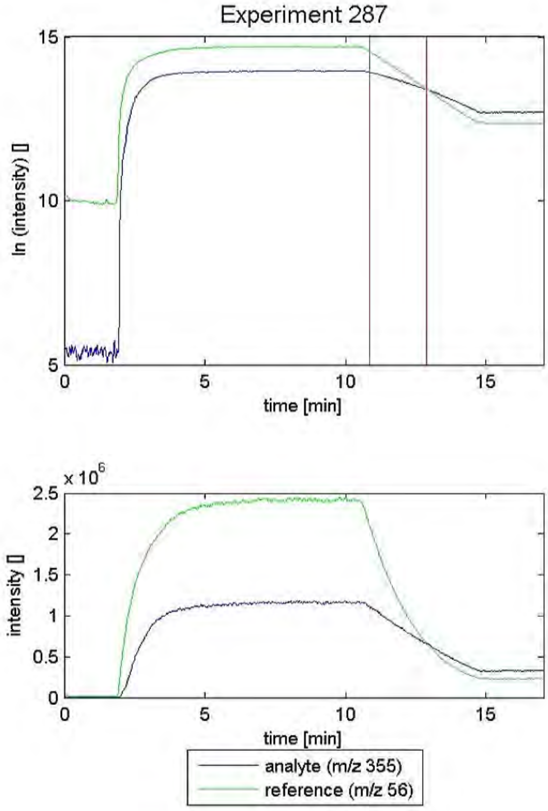

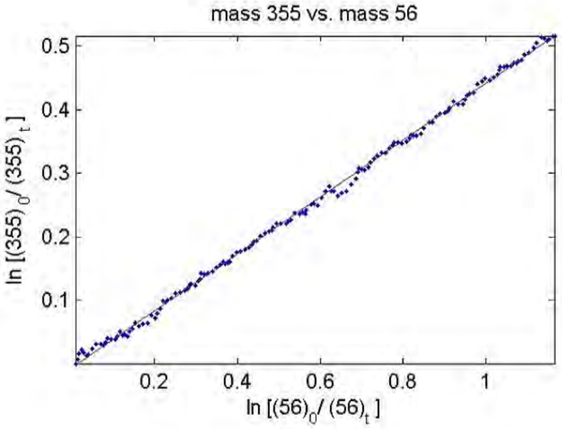

y= -0.007 + 0.448 * x
r= 0.999 n= 151
oven temperature: 60°C
analyte rate constant:
3.56e-12 cm^3 $molec^{-3}$ sec^{-1}
uncertainty range:
[3.23e-12;3.93e-12]
ln(rate)=-26.36
reference shift: 0.0 minutes

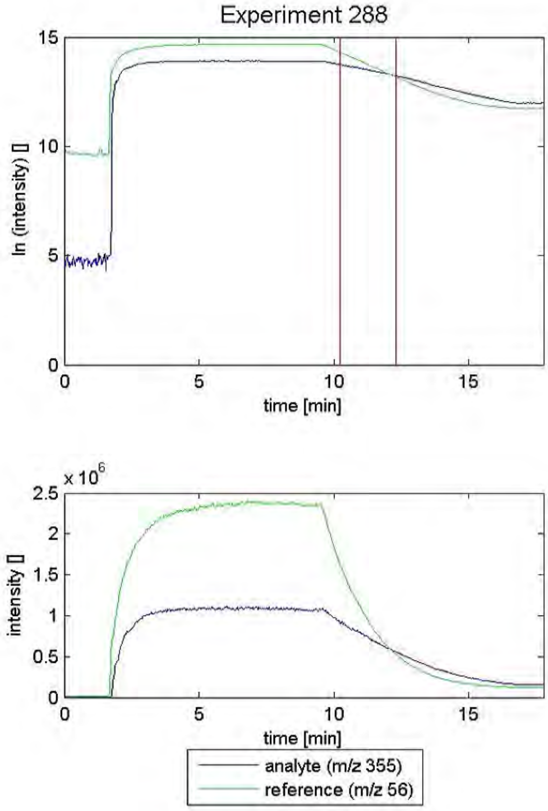

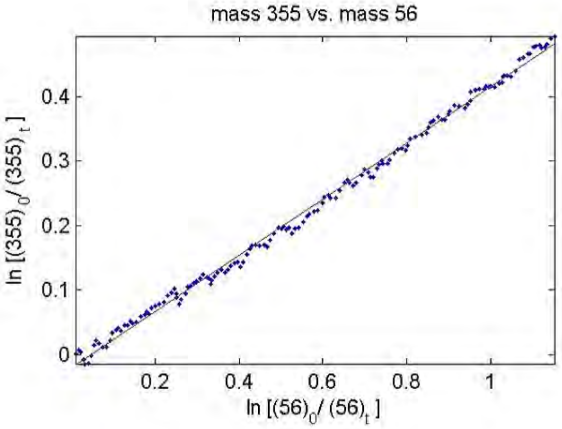

y= -0.021 + 0.435 * x
r= 0.998 n= 151
oven temperature: 60°C
analyte rate constant:
3.46e-12 cm^3 $molec^{-3}$ sec^{-1}
uncertainty range:
[3.13e-12;3.82e-12]
ln(rate)=-26.39
reference shift: 0.0 minutes

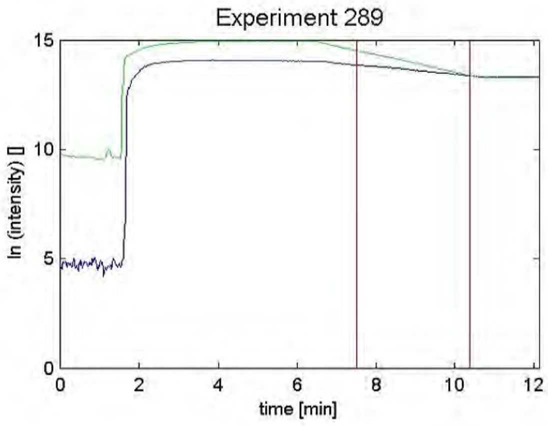

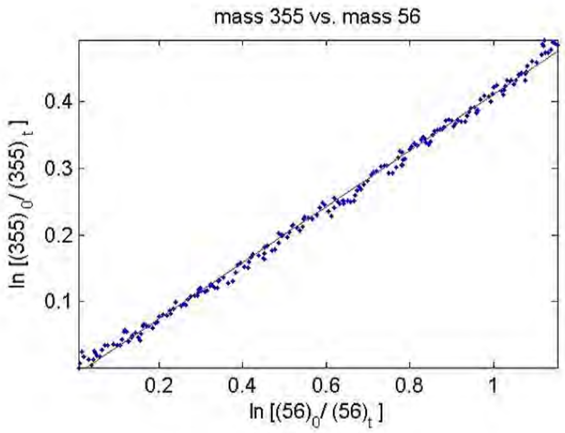

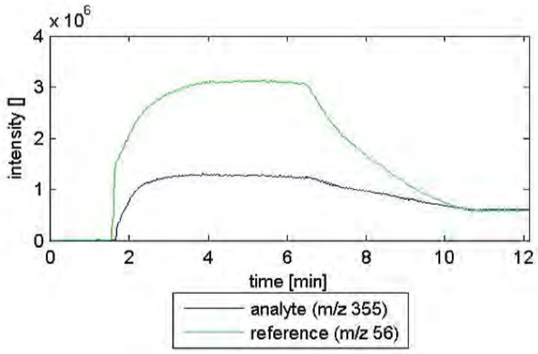

y= -0.011 + 0.422 * x
r= 0.998 n= 201
oven temperature: 50°C
analyte rate constant:
3.23e-12 cm^3 molec^{-3} sec^{-1}
uncertainty range:
[2.92e-12;3.58e-12]
ln(rate)=-26.46
reference shift: 0.0 minutes

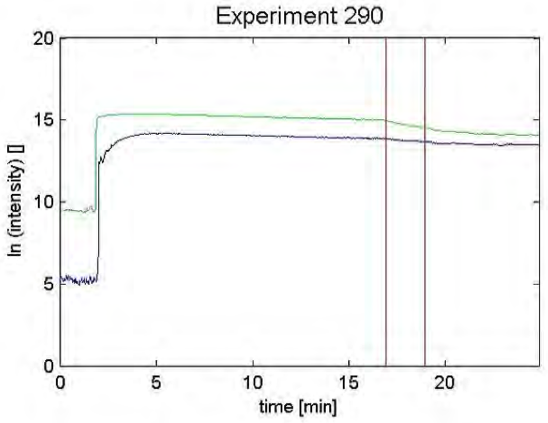

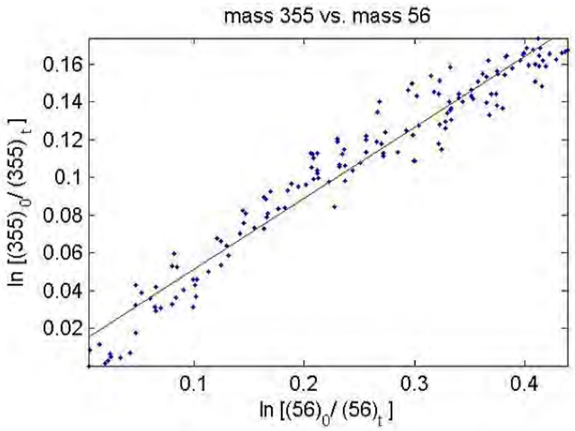

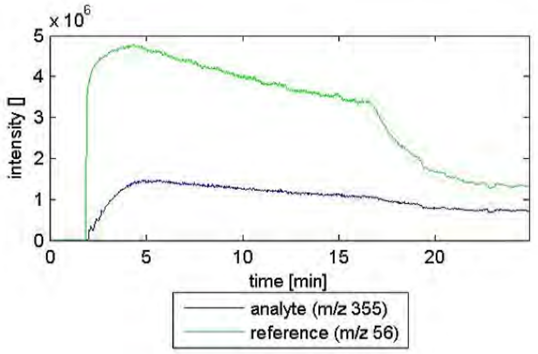

y= 0.014 + 0.375 * x
r= 0.972 n= 151
oven temperature: 50°C
analyte rate constant:
2.87e-12 cm^3 molec^{-3} sec^{-1}
uncertainty range:
[2.59e-12;3.18e-12]
ln(rate)=-26.58
reference shift: 0.0 minutes

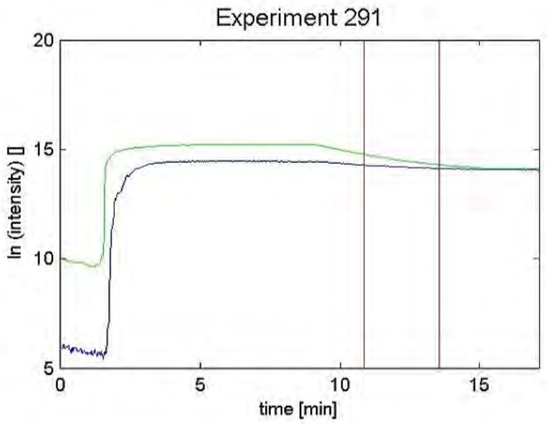

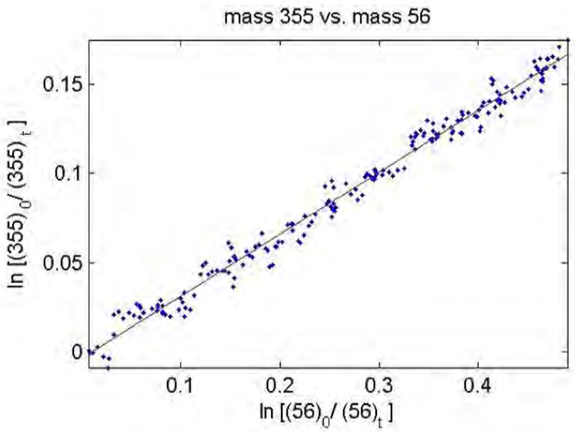

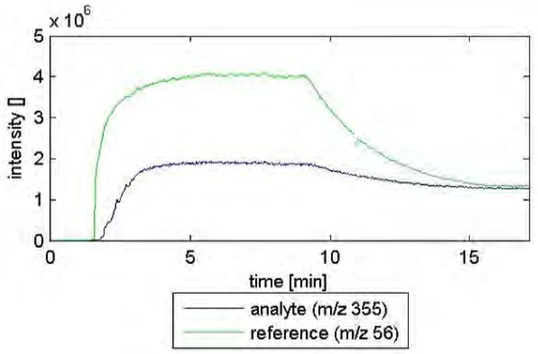

y= -0.004 + 0.348 * x
r= 0.992 n= 201
oven temperature: 50°C
analyte rate constant:
2.66e-12 cm^3 $molec^{-3}$ sec^{-1}
uncertainty range:
[2.40e-12;2.95e-12]
ln(rate)=-26.65
reference shift: 0.0 minutes

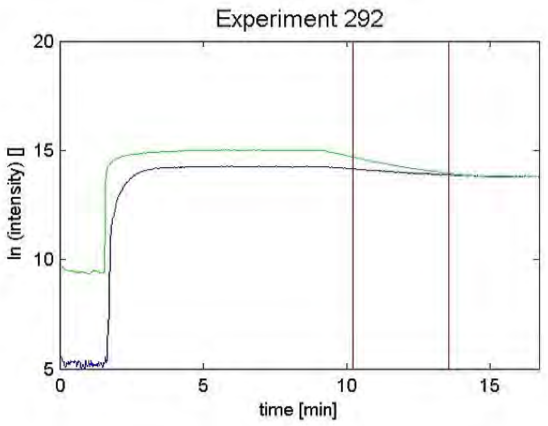

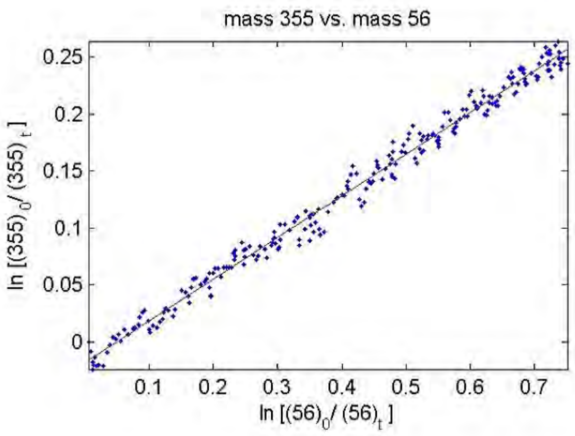

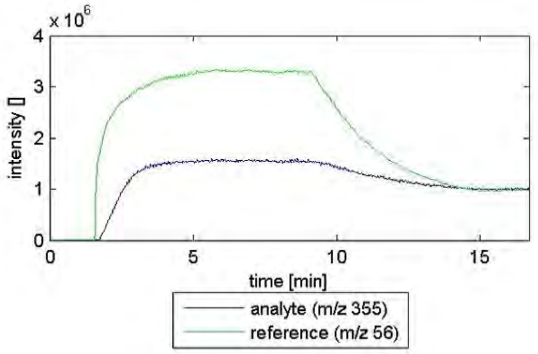

y= -0.018 + 0.365 * x
r= 0.995 n= 251
oven temperature: 50°C
analyte rate constant:
2.79e-12 cm^3 $molec^{-3}$ sec^{-1}
uncertainty range:
[2.52e-12;3.09e-12]
ln(rate)=-26.60
reference shift: 0.0 minutes

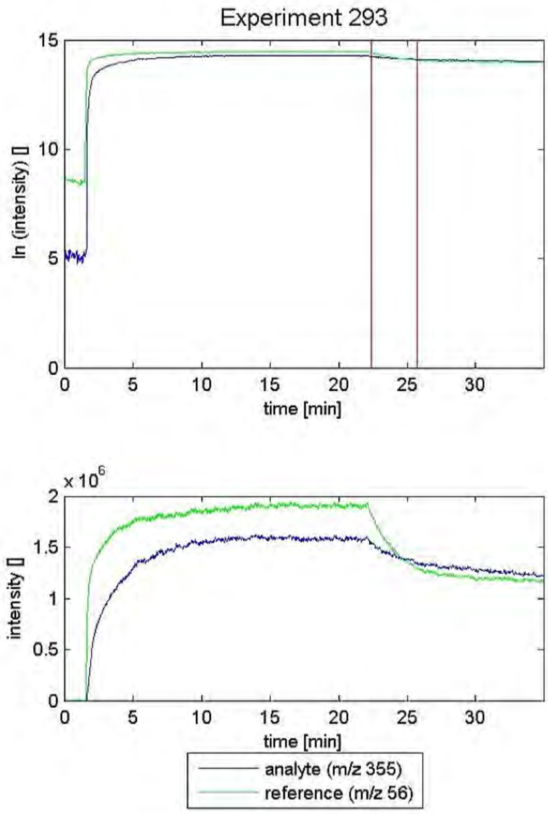

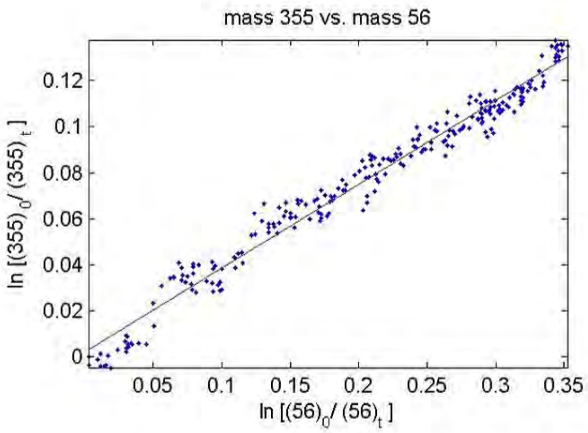

y= 0.002 + 0.364 * x
r= 0.986 n= 251
oven temperature: 70°C
analyte rate constant:
3.00e-12 cm^3 $molec^{-3}$ sec^{-1}
uncertainty range:
[2.73e-12;3.30e-12]
ln(rate)=-26.53
reference shift: 0.0 minutes

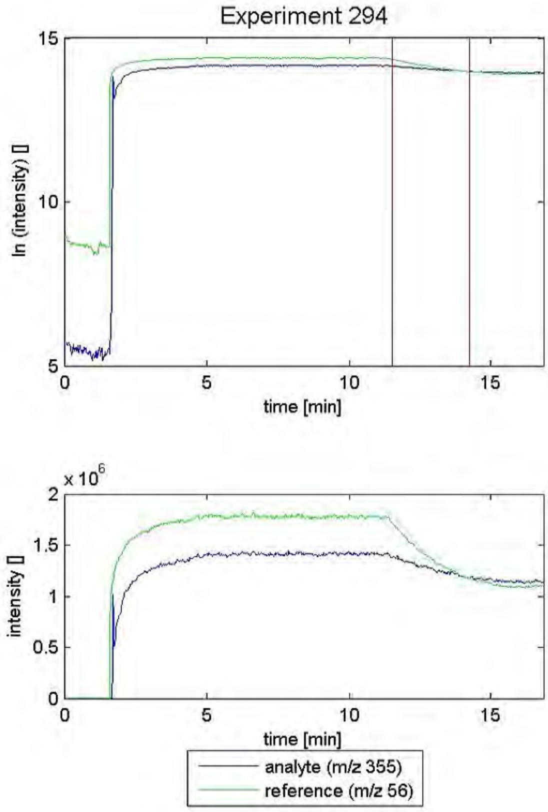

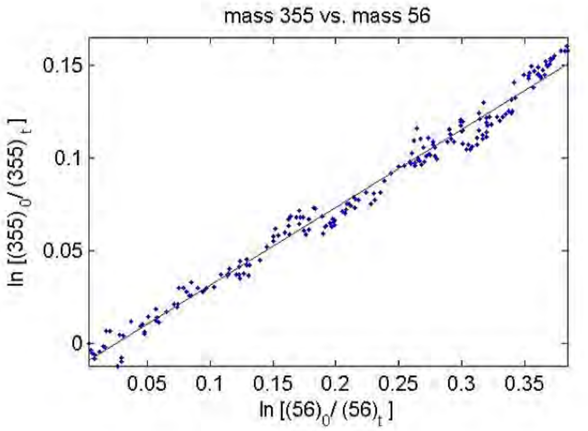

y= -0.011 + 0.420 * x
r= 0.992 n= 201
oven temperature: 70°C
analyte rate constant:
3.46e-12 cm^3 $molec^{-3}$ sec^{-1}
uncertainty range:
[3.14e-12;3.81e-12]
ln(rate)=-26.39
reference shift: 0.0 minutes

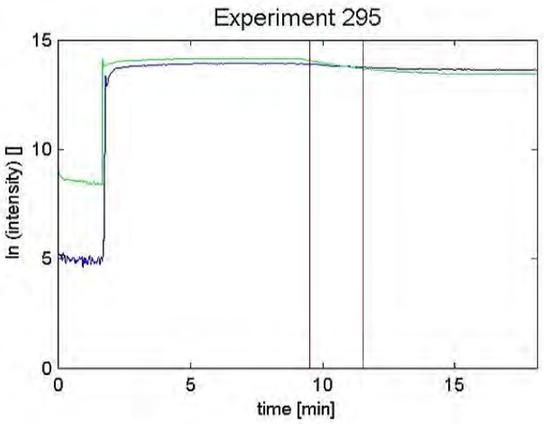

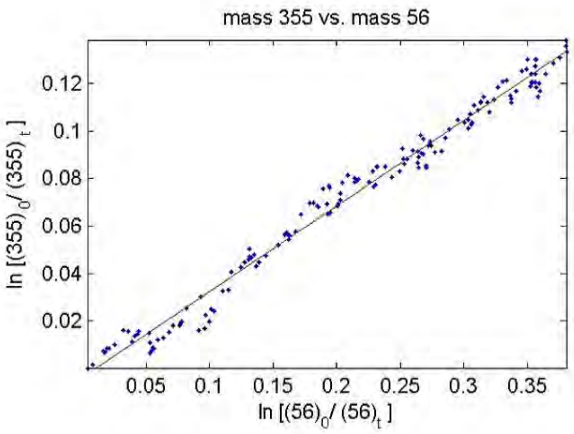

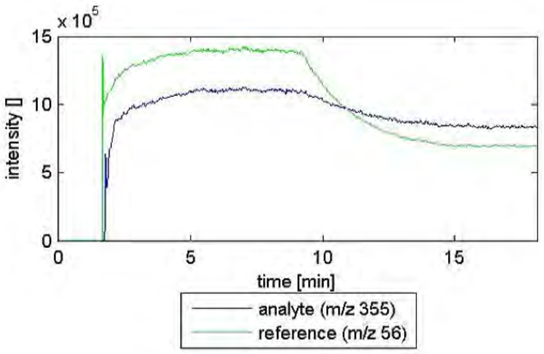

y= -0.004 + 0.361 * x
r= 0.992 n= 151
oven temperature: 70°C
analyte rate constant:
2.97e-12 cm^3 $molec^{-3}$ sec^{-1}
uncertainty range:
[2.70e-12;3.27e-12]
ln(rate)=-26.54
reference shift: 0.0 minutes

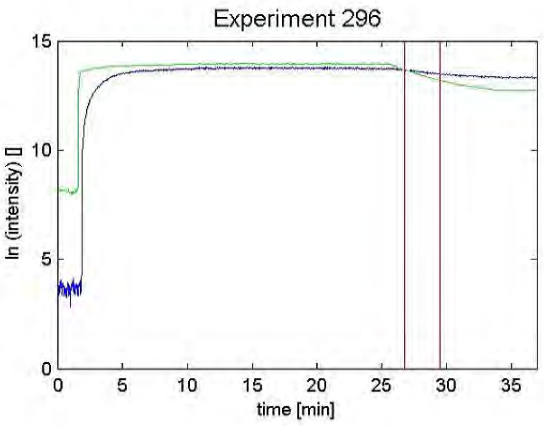

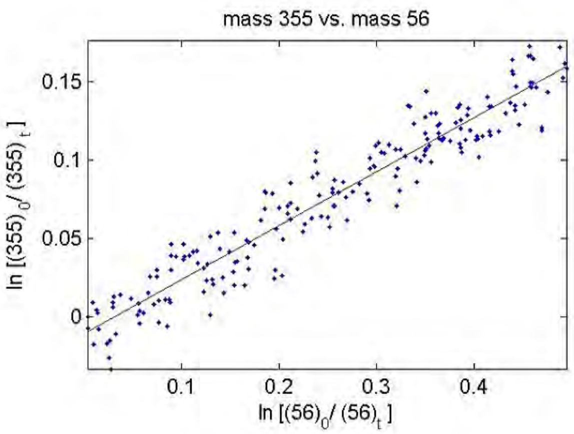

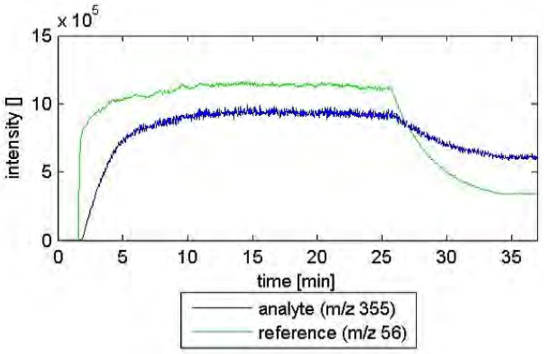

y= -0.011 + 0.344 * x
r= 0.958 n= 201
oven temperature: 40°C
analyte rate constant:
2.54e-12 cm^3 $molec^{-3}$ sec^{-1}
uncertainty range:
[2.29e-12;2.82e-12]
ln(rate)=-26.70
reference shift: 0.0 minutes

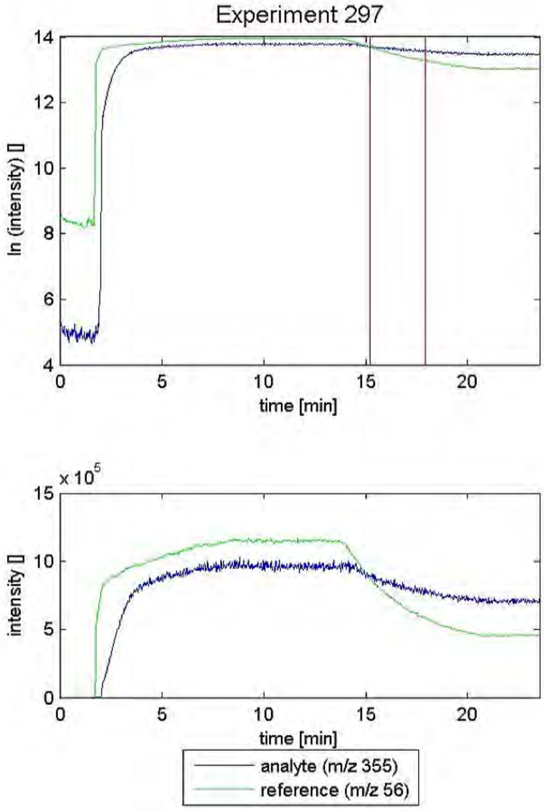

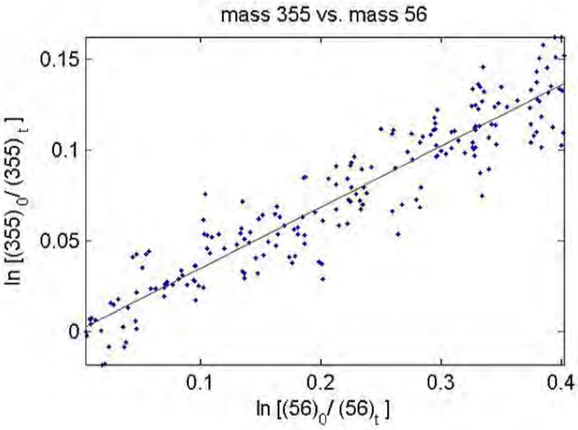

y= 0.001 + 0.337 * x
r= 0.937 n= 201
oven temperature: 40°C
analyte rate constant:
2.49e-12 cm^3 $molec^{-3}$ sec^{-1}
uncertainty range:
[2.24e-12;2.76e-12]
ln(rate)=-26.72
reference shift: 0.0 minutes

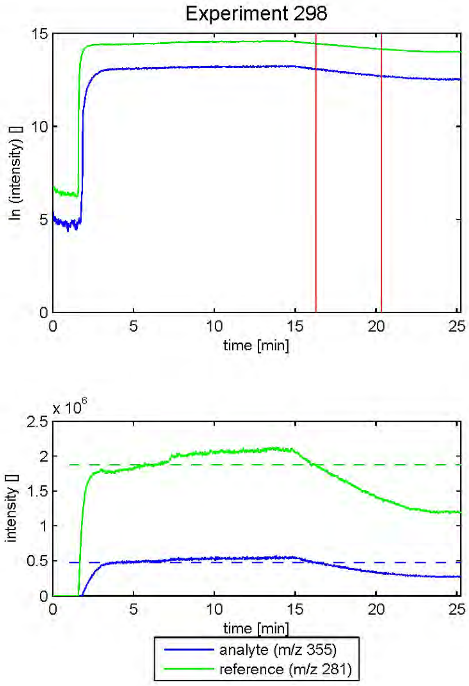

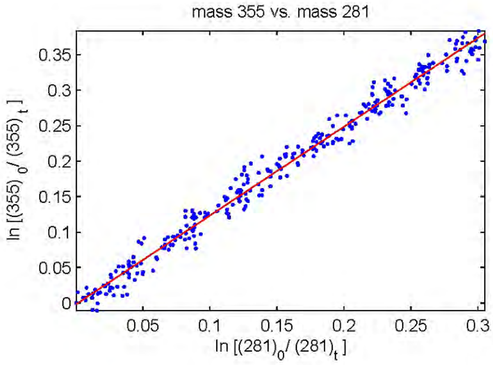

y= −0.002 + 1.253 * x
r= 0.992
oven temperature: 40°C
relative rate
1.25e+00
reference shift: 0 scans

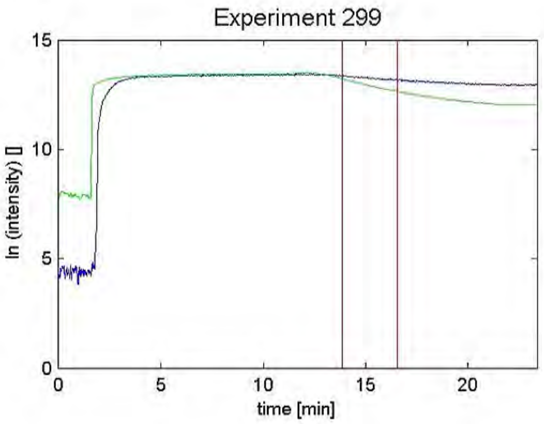

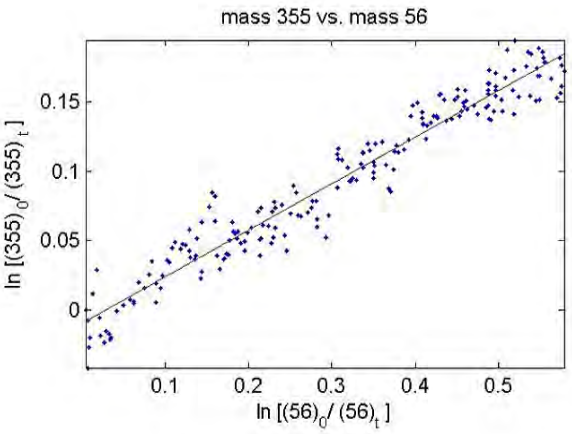

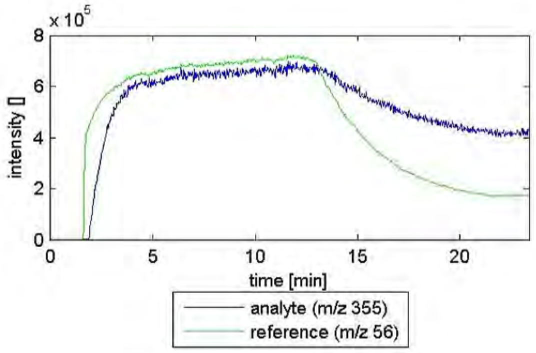

y= -0.010 + 0.336 * x
r= 0.970 n= 201
oven temperature: 40°C
analyte rate constant:
2.48e-12 cm^3 $molec^{-3}$ sec^{-1}
uncertainty range:
[2.23e-12;2.76e-12]
ln(rate)=-26.72
reference shift: 0.0 minutes

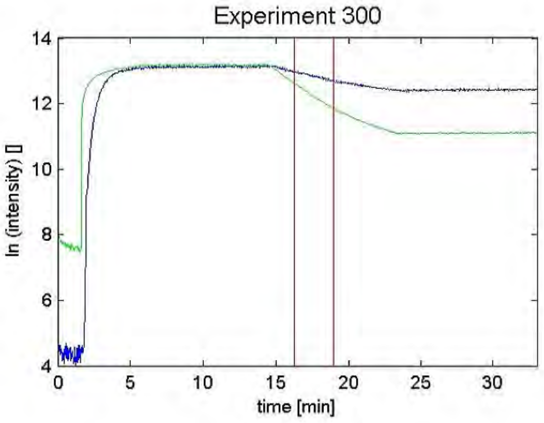

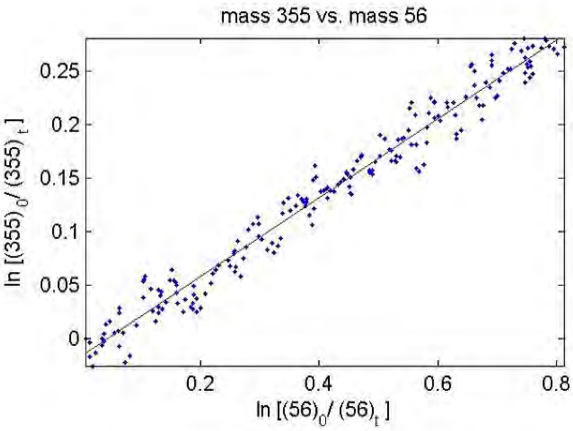

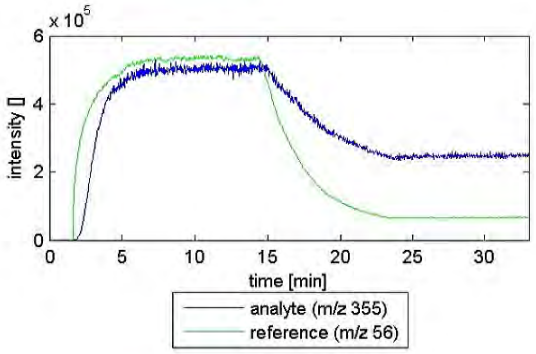

y= -0.016 + 0.370 * x
r= 0.985 n= 201
oven temperature: 40°C
analyte rate constant:
2.73e-12 cm^3 $molec^{-3}$ sec^{-1}
uncertainty range:
[2.46e-12;3.03e-12]
ln(rate)=-26.63
reference shift: 0.0 minutes

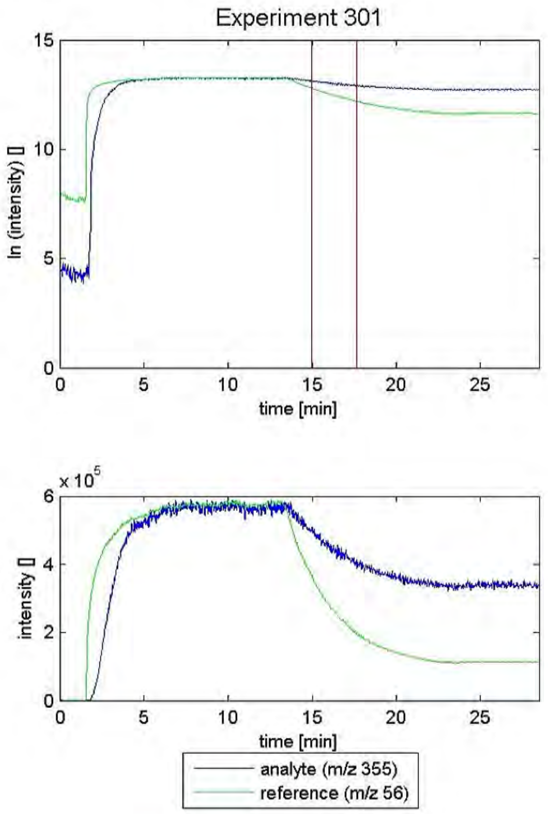

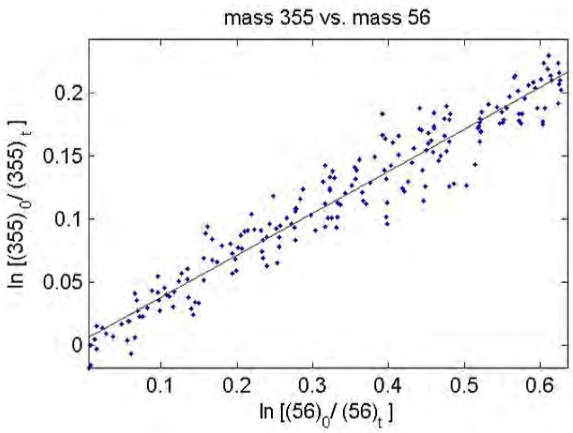

y= 0.004 + 0.333 * x
r= 0.965 n= 201
oven temperature: 40°C
analyte rate constant:
2.46e-12 cm^3 $molec^{-3}$ sec^{-1}
uncertainty range:
[2.21e-12;2.73e-12]
ln(rate)=-26.73
reference shift: 0.0 minutes

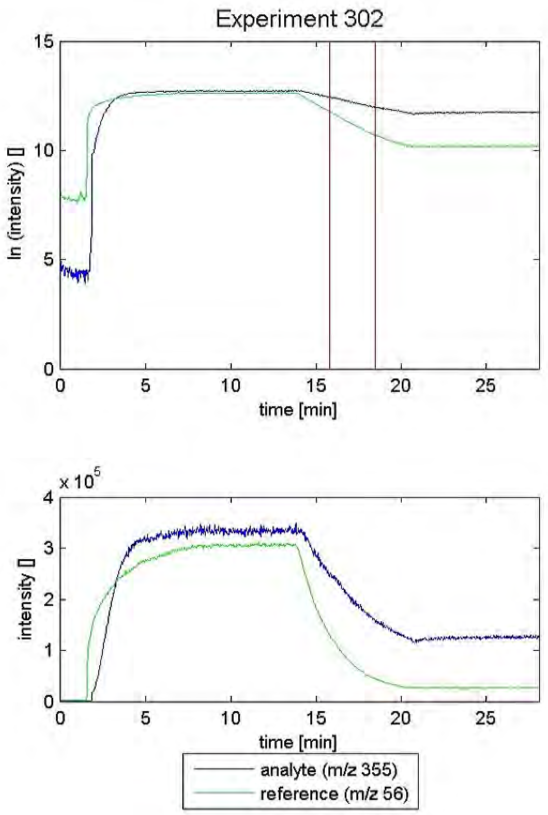

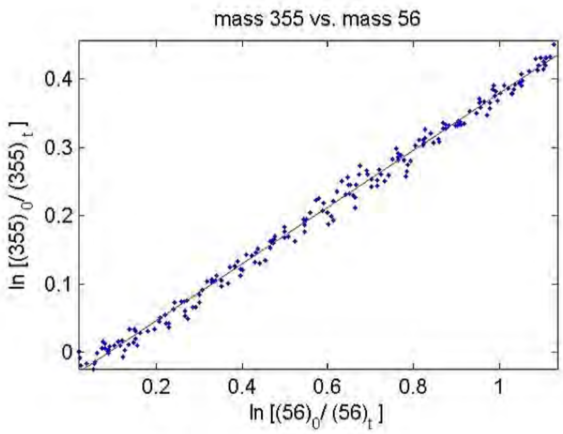

y= -0.037 + 0.416 * x
r= 0.997 n= 201
oven temperature: 40°C
analyte rate constant:
3.07e-12 cm^3 $molec^{-3}$ sec^{-1}
uncertainty range:
[2.76e-12;3.41e-12]
ln(rate)=-26.51
reference shift: 0.0 minutes

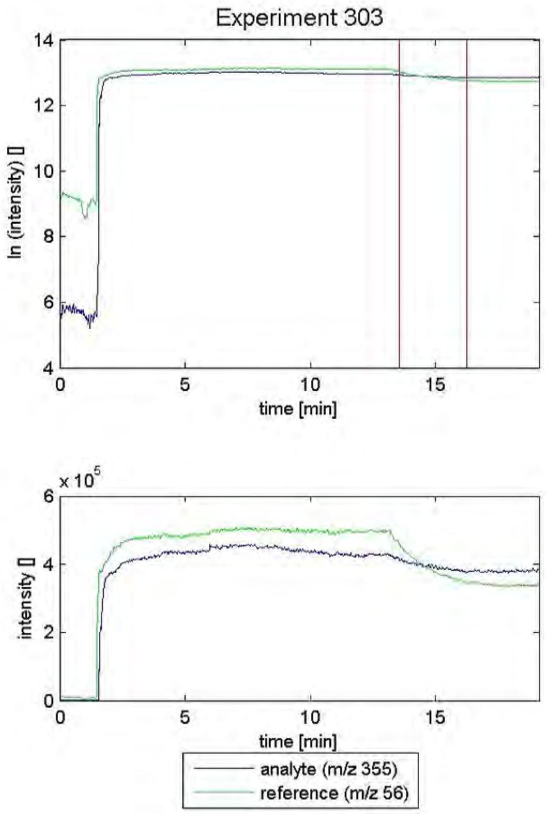

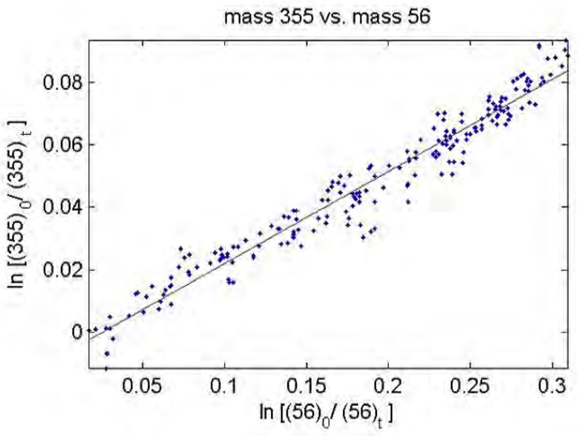

y= -0.008 + 0.295 * x
r= 0.976 n= 201
oven temperature: 80°C
analyte rate constant:
2.52e-12 cm^3 $molec^{-3}$ sec^{-1}
uncertainty range:
[2.29e-12;2.77e-12]
ln(rate)=-26.71
reference shift: 0.0 minutes

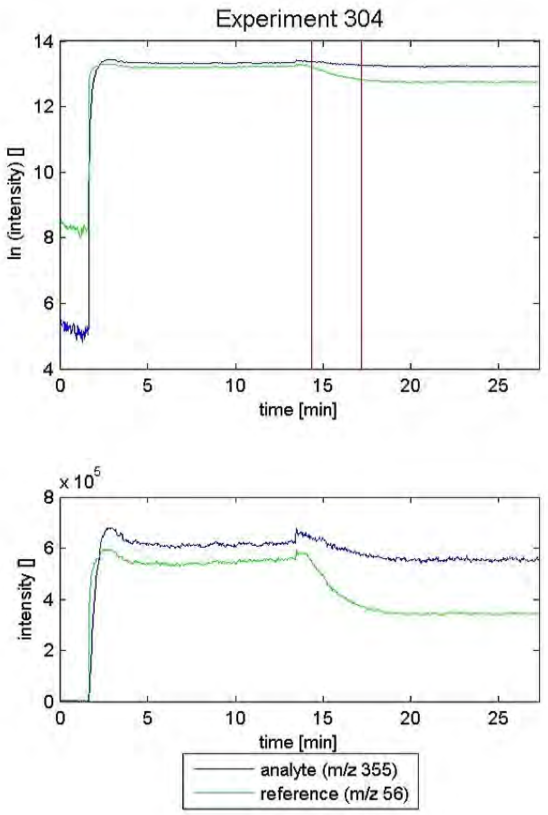

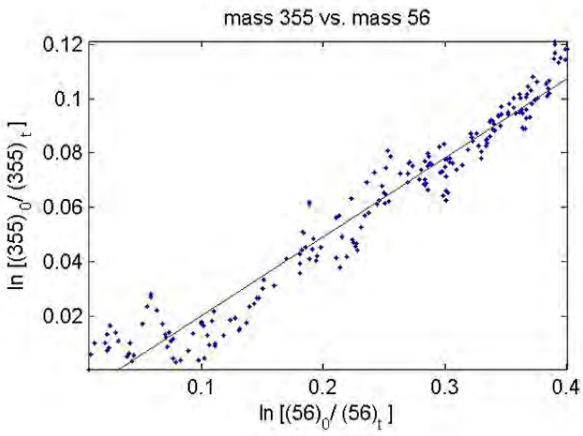

y= -0.009 + 0.290 * x
r= 0.973 n= 201
oven temperature: 80°C
analyte rate constant:
2.48e-12 cm^3 $molec^{-3}$ sec^{-1}
uncertainty range:
[2.26e-12;2.72e-12]
ln(rate)=-26.72
reference shift: 0.0 minutes

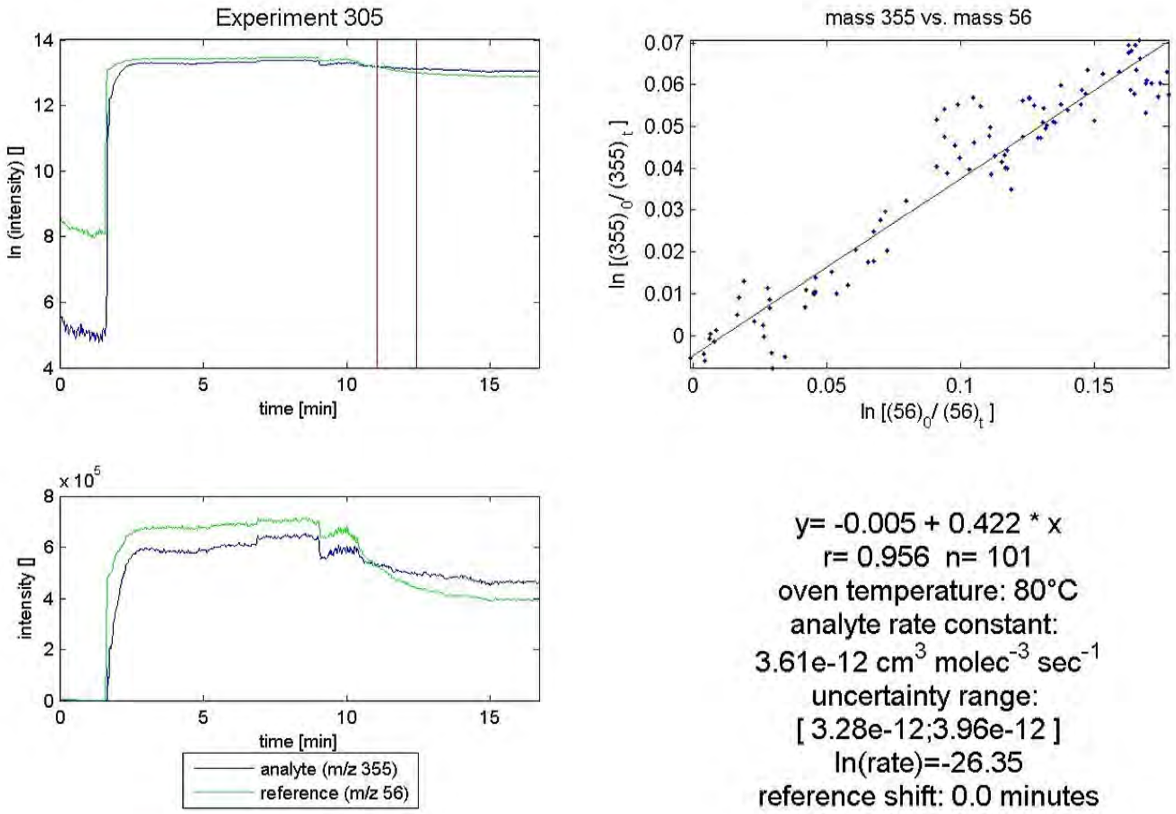
Experiment 305
ln (intensity) []
time [min]
intensity []
x 10^5
analyte (m/z 355)
reference (m/z 56)
mass 355 vs. mass 56
ln [(355)0/ (355)t]
ln [(56)0/ (56)t]
y= -0.005 + 0.422 * x
r= 0.956 n= 101
oven temperature: 80°C
analyte rate constant:
3.61e-12 cm3 molec-3 sec-1
uncertainty range:
[3.28e-12;3.96e-12]
ln(rate)=-26.35
reference shift: 0.0 minutes

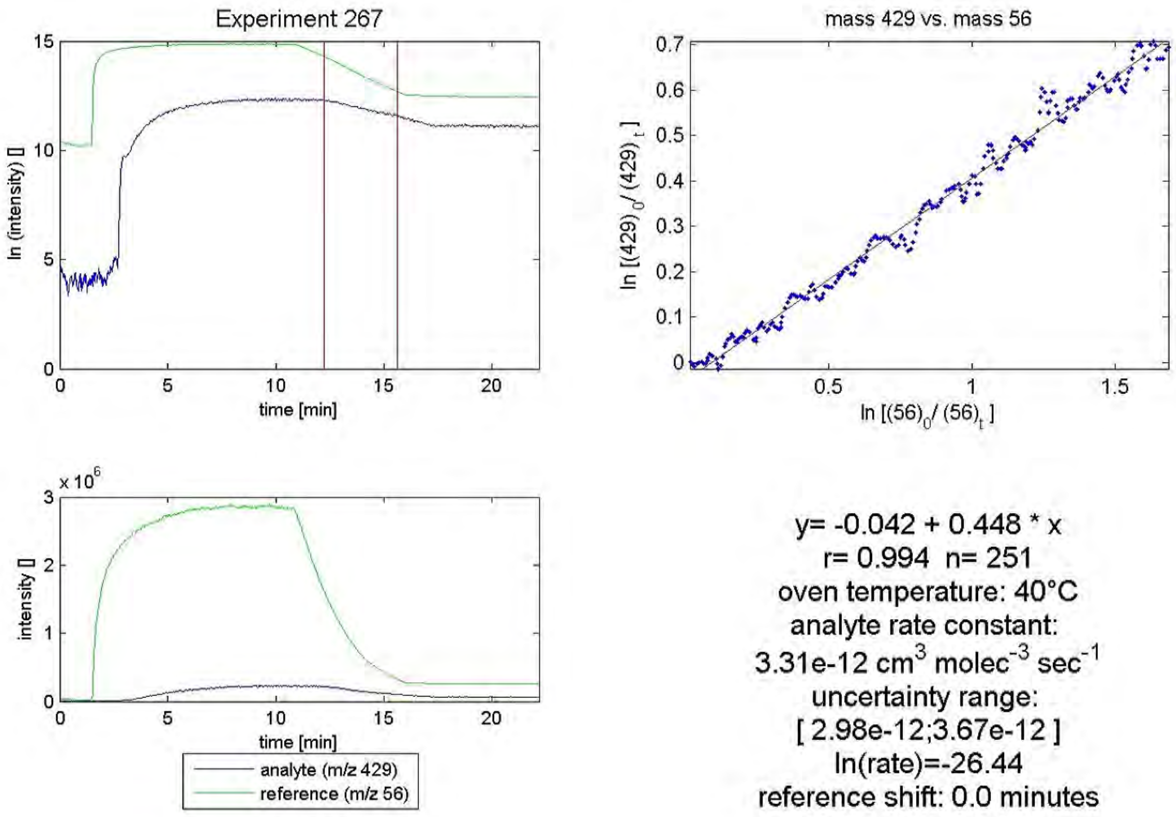
Experiment 267
ln (intensity) []
time [min]
intensity []
x 10^6
analyte (m/z 429)
reference (m/z 56)
mass 429 vs. mass 56
ln [(429)0/ (429)t]
ln [(56)0/ (56)t]
y= -0.042 + 0.448 * x
r= 0.994 n= 251
oven temperature: 40°C
analyte rate constant:
3.31e-12 cm3 molec-3 sec-1
uncertainty range:
[2.98e-12;3.67e-12]
ln(rate)=-26.44
reference shift: 0.0 minutes

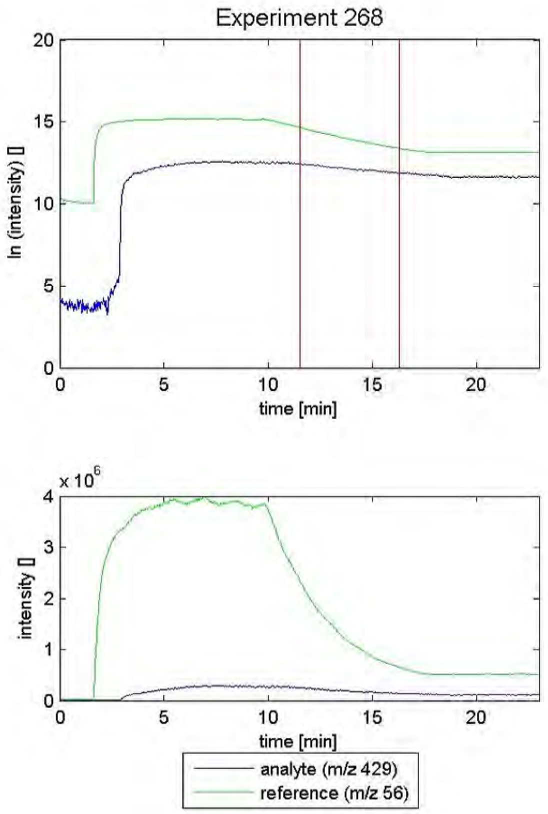

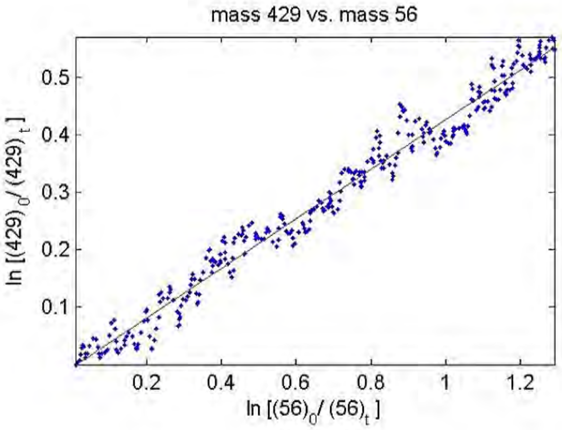

y= -0.005 + 0.431 * x
r= 0.987 n= 351
oven temperature: 40°C
analyte rate constant:
3.18e-12 cm^3 $molec^{-3}$ sec^{-1}
uncertainty range:
[2.87e-12;3.54e-12]
ln(rate)=-26.47
reference shift: 0.0 minutes

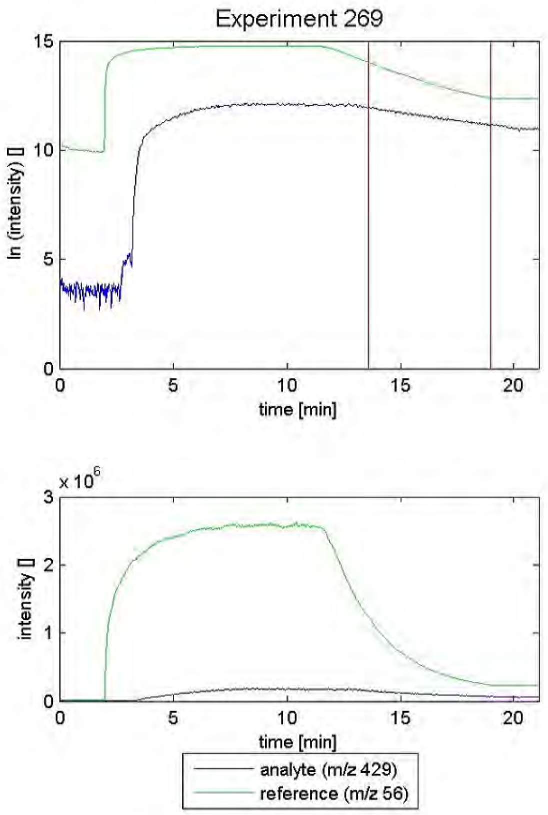

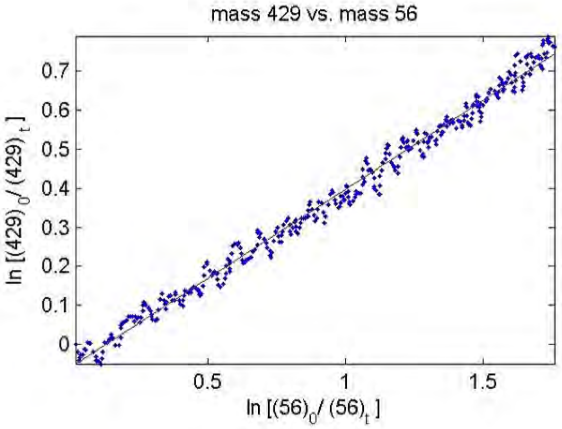

y= -0.058 + 0.454 * x
r= 0.994 n= 401
oven temperature: 40°C
analyte rate constant:
3.35e-12 cm^3 $molec^{-3}$ sec^{-1}
uncertainty range:
[3.02e-12;3.73e-12]
ln(rate)=-26.42
reference shift: 0.0 minutes

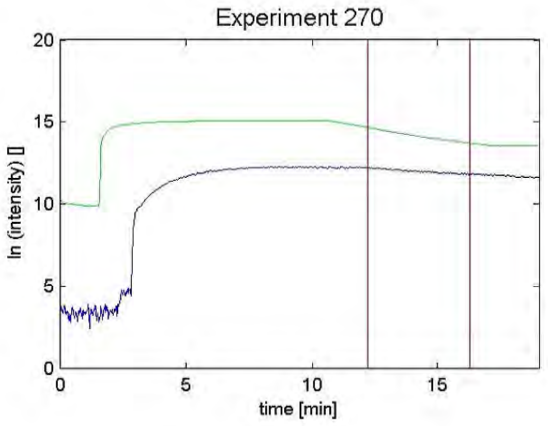

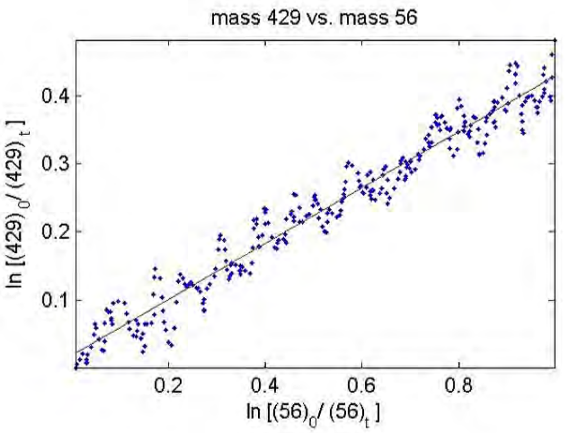

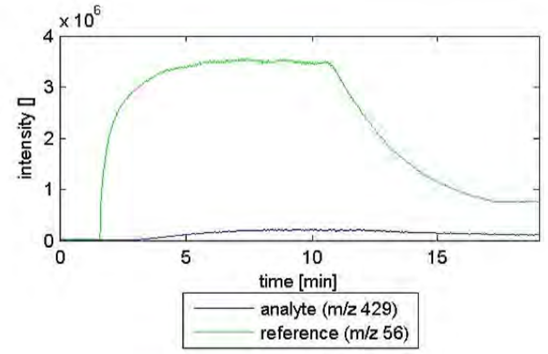

y= 0.018 + 0.412 * x
r= 0.978 n= 301
oven temperature: 40°C
analyte rate constant:
3.04e-12 cm^3 $molec^{-3}$ sec^{-1}
uncertainty range:
[2.74e-12;3.38e-12]
ln(rate)=-26.52
reference shift: 0.0 minutes

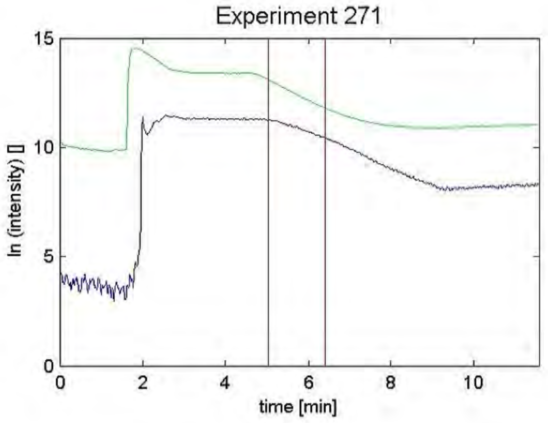

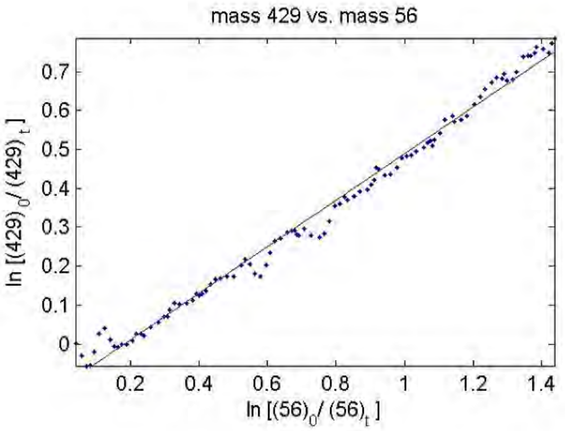

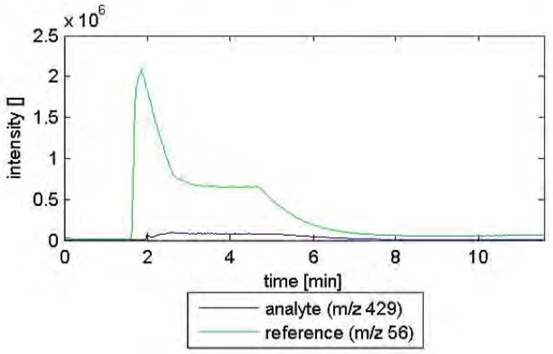

y= -0.109 + 0.598 * x
r= 0.994 n= 101
oven temperature: 60°C
analyte rate constant:
4.75e-12 cm^3 $molec^{-3}$ sec^{-1}
uncertainty range:
[4.30e-12;5.24e-12]
ln(rate)=-26.07
reference shift: 0.0 minutes

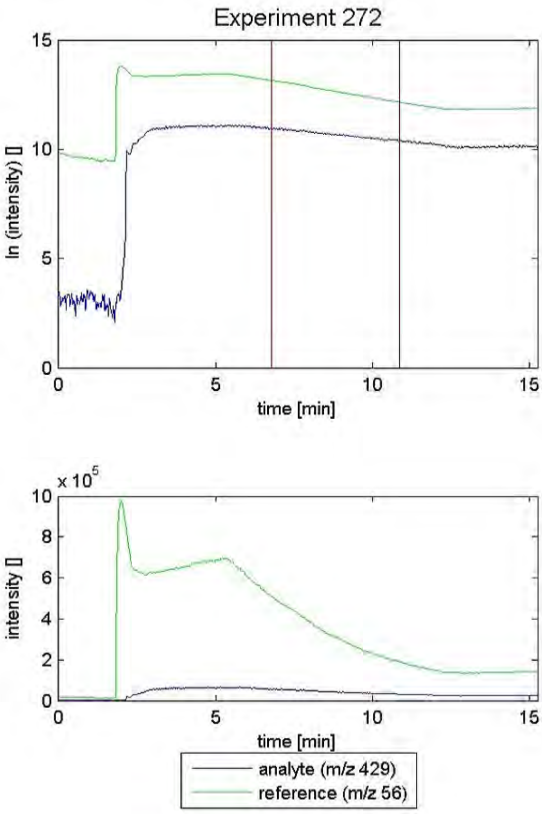

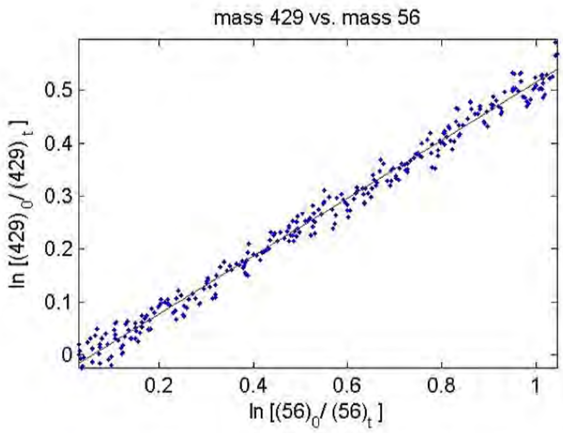

y= -0.032 + 0.546 * x
r= 0.994 n= 301
oven temperature: 60°C
analyte rate constant:
4.34e-12 cm^3 $molec^{-3}$ sec^{-1}
uncertainty range:
[3.93e-12;4.79e-12]
ln(rate)=-26.16
reference shift: 0.0 minutes

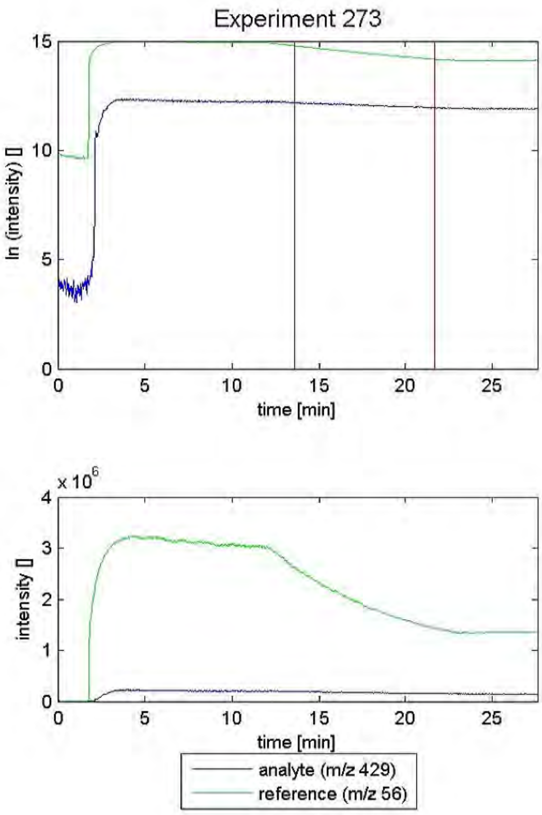

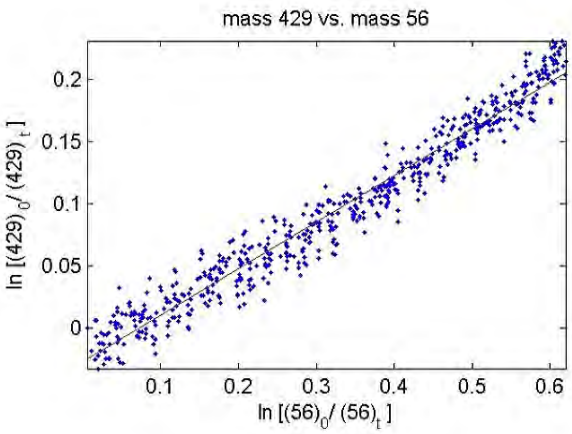

y= -0.027 + 0.375 * x
r= 0.979 n= 601
oven temperature: 60°C
analyte rate constant:
2.98e-12 cm^3 $molec^{-3}$ sec^{-1}
uncertainty range:
[2.69e-12;3.29e-12]
ln(rate)=-26.54
reference shift: 0.0 minutes

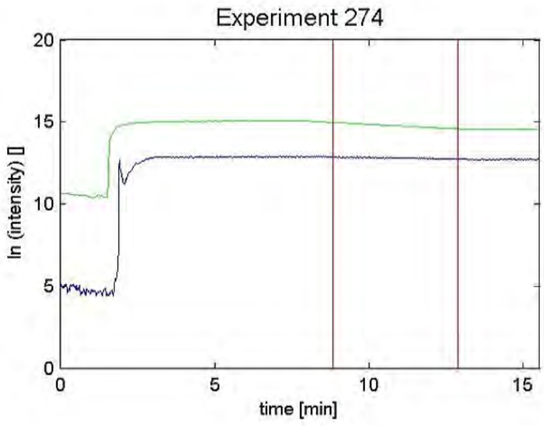

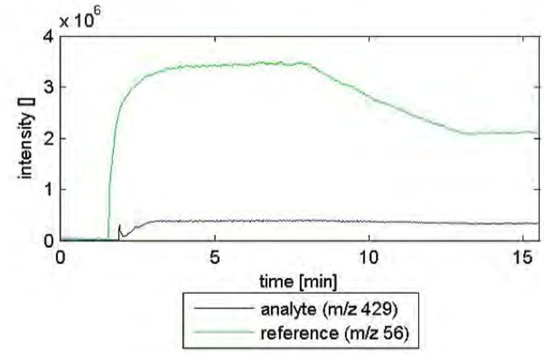

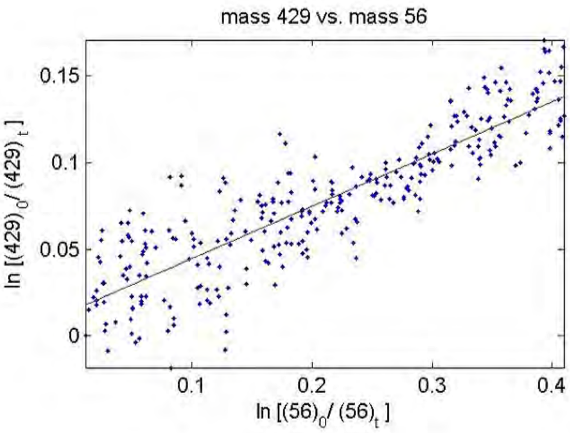

y= 0.014 + 0.301 * x
r= 0.879 n= 301
oven temperature: 60°C
analyte rate constant:
2.39e-12 cm^3 molec^{-3} sec^{-1}
uncertainty range:
[2.17e-12;2.64e-12]
ln(rate)=-26.76
reference shift: 0.0 minutes

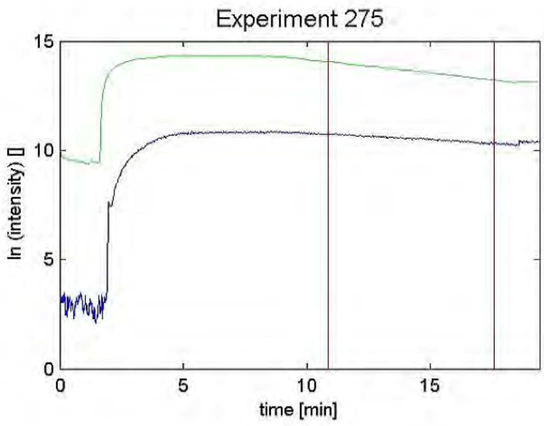

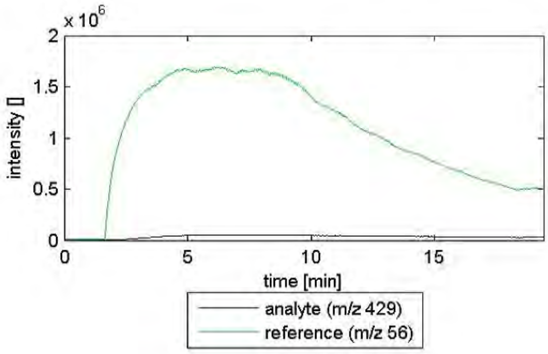

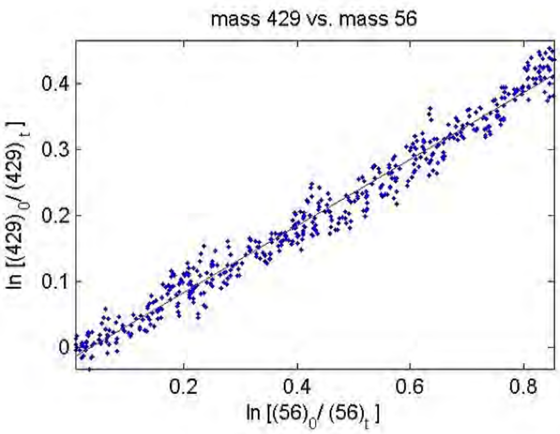

y= -0.017 + 0.504 * x
r= 0.986 n= 501
oven temperature: 60°C
analyte rate constant:
4.00e-12 cm^3 molec^{-3} sec^{-1}
uncertainty range:
[3.63e-12;4.42e-12]
ln(rate)=-26.24
reference shift: 0.0 minutes

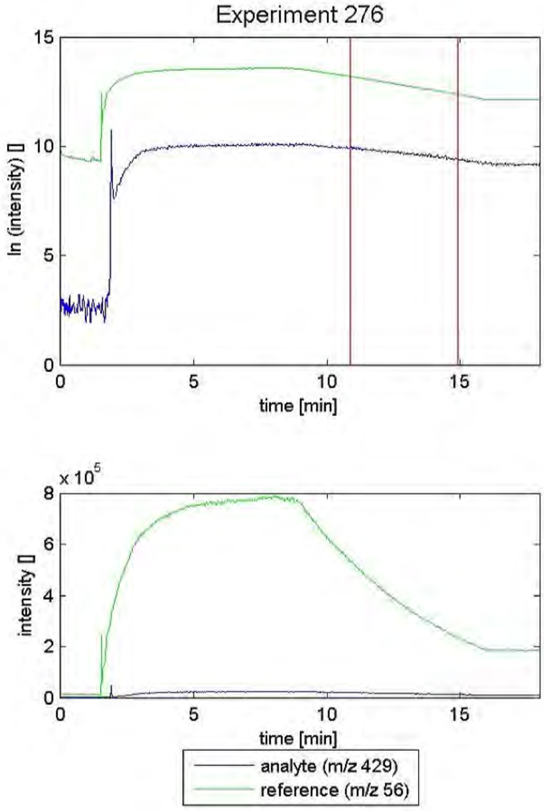

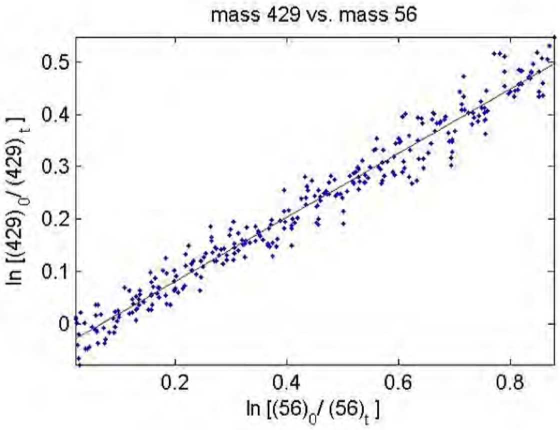

y= -0.042 + 0.612 * x
r= 0.982 n= 301
oven temperature: 60°C
analyte rate constant:
4.86e-12 cm^3 $molec^{-3}$ sec^{-1}
uncertainty range:
[4.40e-12;5.37e-12]
ln(rate)=-26.05
reference shift: 0.0 minutes

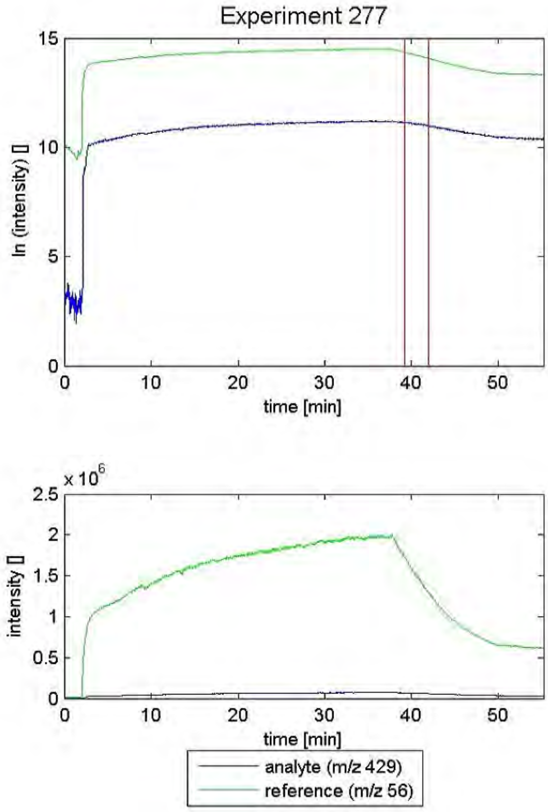

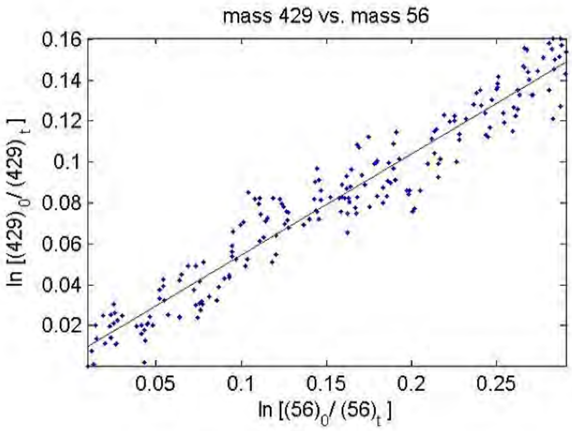

y= 0.005 + 0.495 * x
r= 0.966 n= 201
oven temperature: 80°C
analyte rate constant:
4.22e-12 cm^3 $molec^{-3}$ sec^{-1}
uncertainty range:
[3.85e-12;4.64e-12]
ln(rate)=-26.19
reference shift: 0.0 minutes

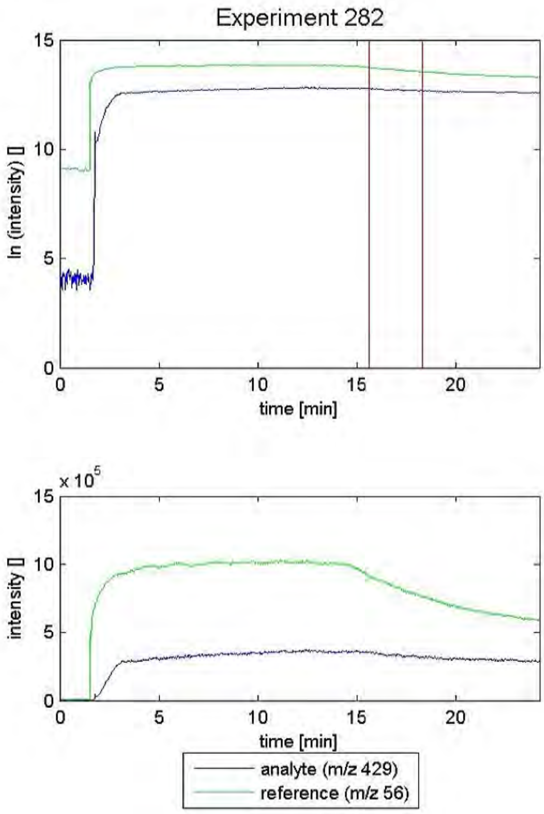

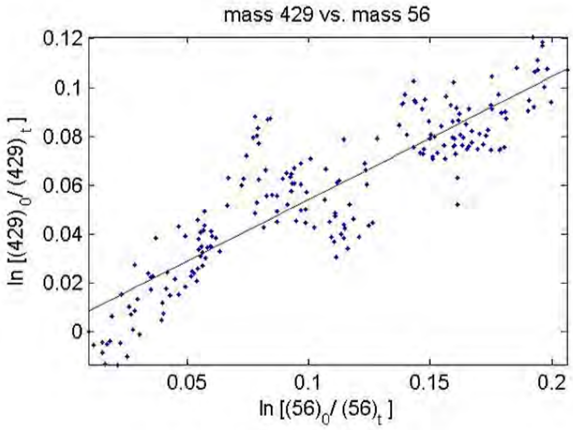

y= 0.004 + 0.503 * x
r= 0.880 n= 201
oven temperature: 80°C
analyte rate constant:
4.30e-12 cm^3 $molec^{-3}$ sec^{-1}
uncertainty range:
[3.91e-12;4.72e-12]
ln(rate)=-26.17
reference shift: 0.0 minutes

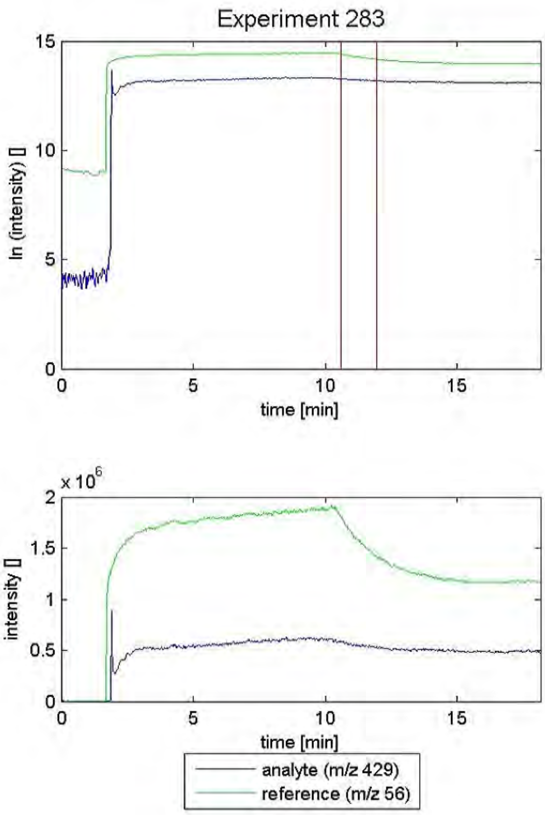

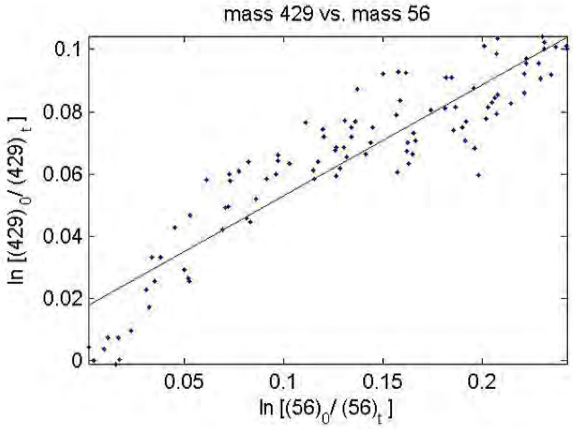

y= 0.017 + 0.357 * x
r= 0.913 n= 101
oven temperature: 80°C
analyte rate constant:
3.05e-12 cm^3 $molec^{-3}$ sec^{-1}
uncertainty range:
[2.77e-12;3.34e-12]
ln(rate)=-26.52
reference shift: 0.0 minutes

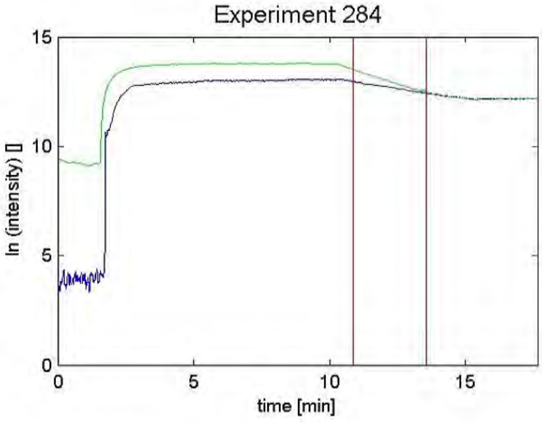

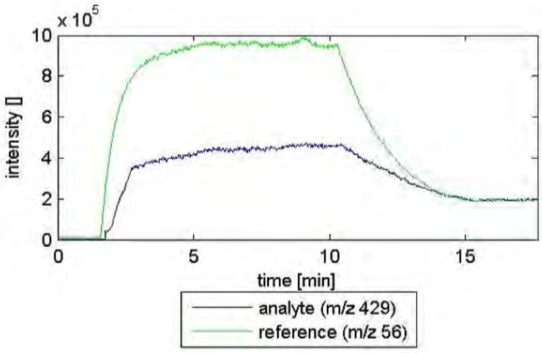

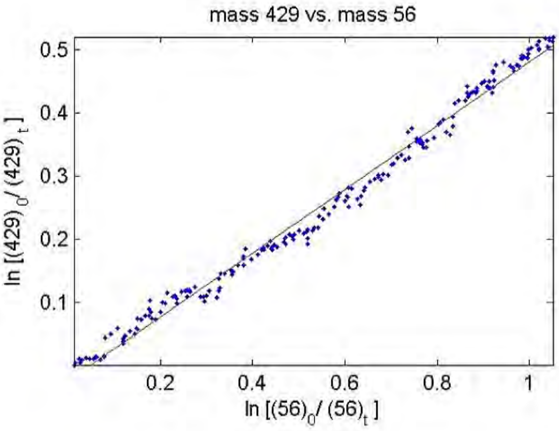

y= -0.025 + 0.505 * x

r= 0.994 n= 201

oven temperature: 80°C

analyte rate constant:

4.31e-12 cm^3 $molec^{-3}$ sec^{-1}

uncertainty range:

[3.93e-12;4.74e-12]

ln(rate)=-26.17

reference shift: 0.0 minutes

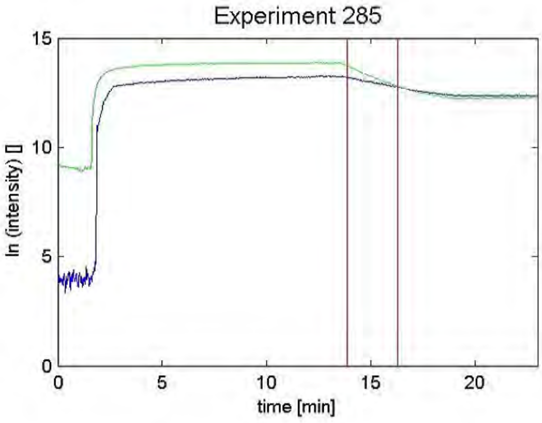

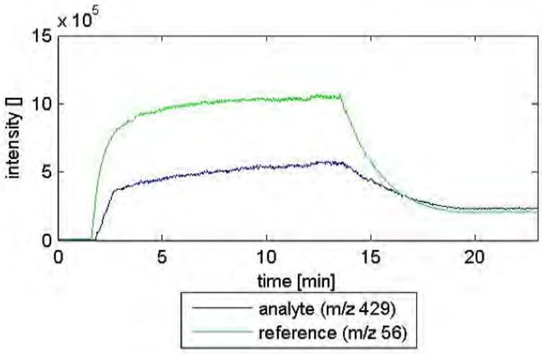

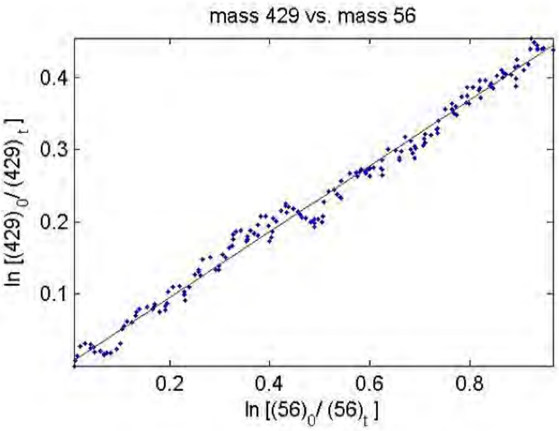

y= 0.003 + 0.457 * x

r= 0.994 n= 181

oven temperature: 80°C

analyte rate constant:

3.90e-12 cm^3 $molec^{-3}$ sec^{-1}

uncertainty range:

[3.55e-12;4.28e-12]

ln(rate)=-26.27

reference shift: 0.0 minutes

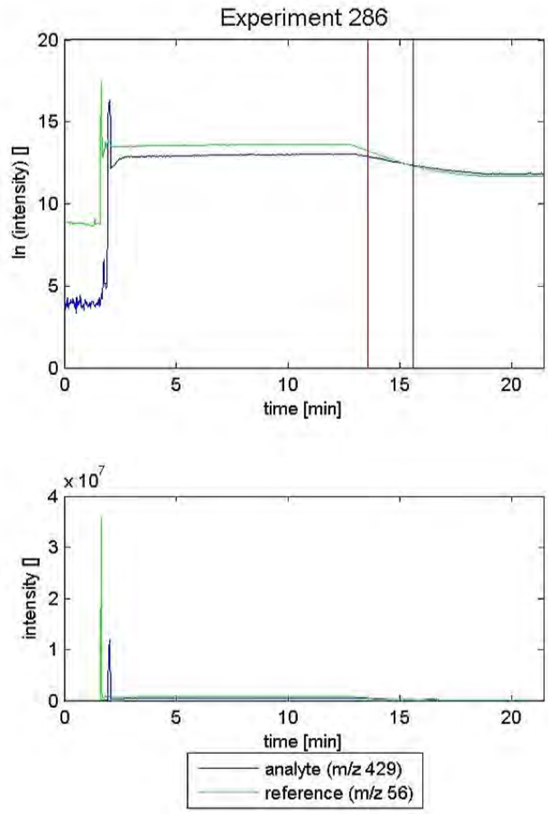

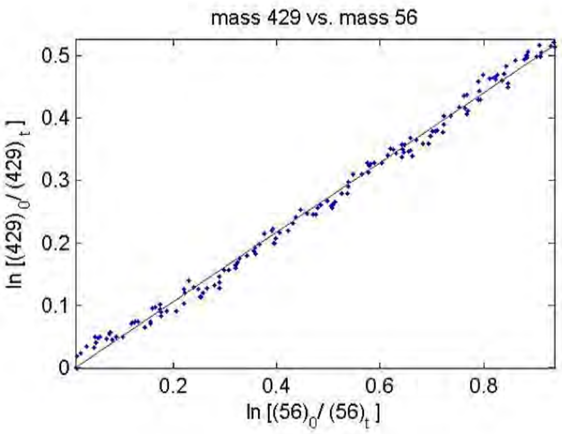

y= -0.007 + 0.559 * x
r= 0.997 n= 151
oven temperature: 80°C
analyte rate constant:
4.77e-12 cm^3 $molec^{-3}$ sec^{-1}
uncertainty range:
[4.34e-12;5.24e-12]
ln(rate)=-26.07
reference shift: 0.0 minutes

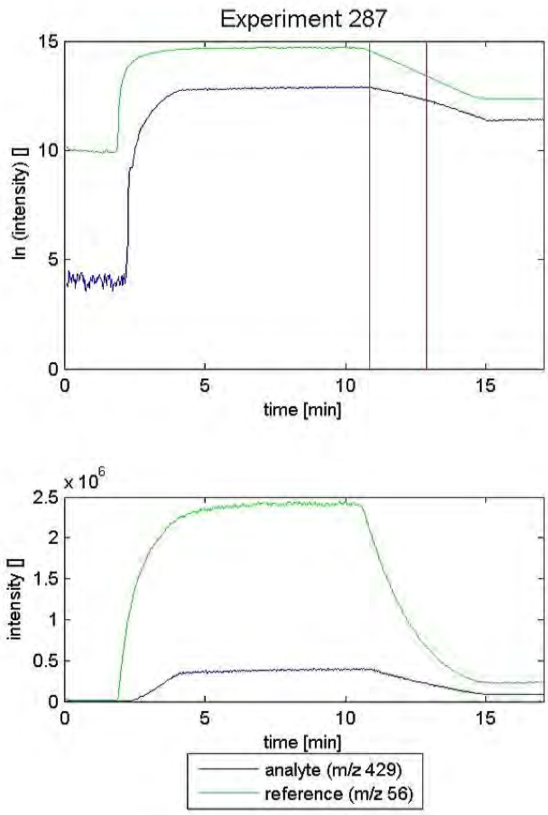

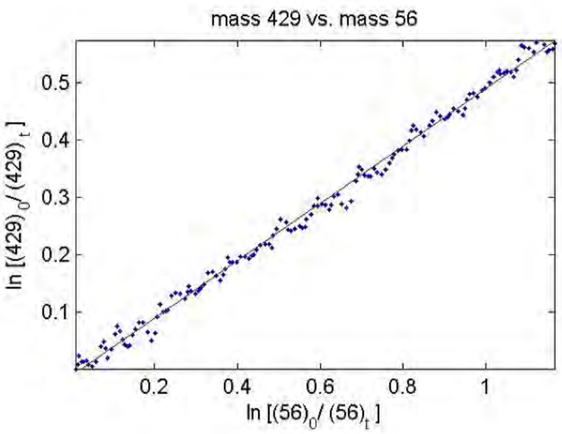

y= -0.012 + 0.502 * x
r= 0.997 n= 151
oven temperature: 60°C
analyte rate constant:
3.99e-12 cm^3 $molec^{-3}$ sec^{-1}
uncertainty range:
[3.61e-12;4.40e-12]
ln(rate)=-26.25
reference shift: 0.0 minutes

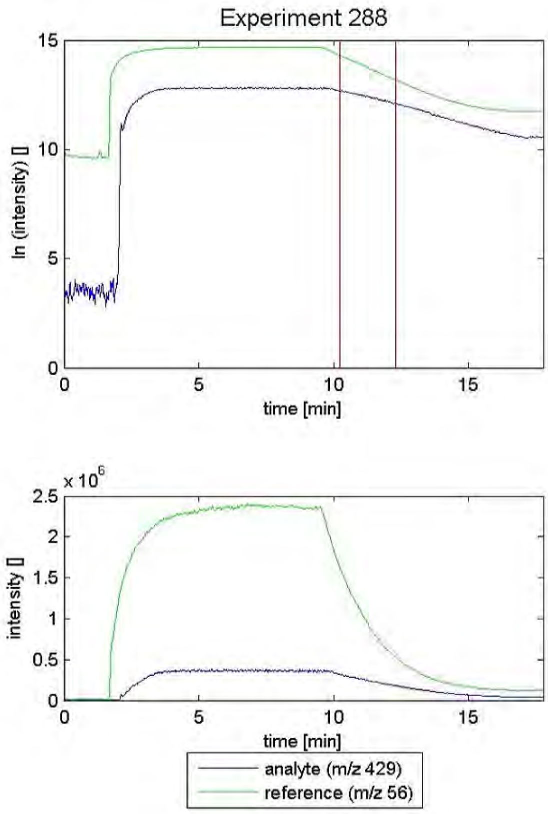

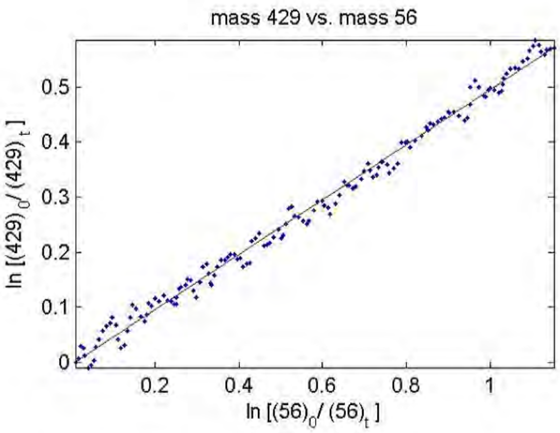

y= -0.004 + 0.499 * x
r= 0.996 n= 151
oven temperature: 60°C
analyte rate constant:
3.96e-12 cm^3 $molec^{-3}$ sec^{-1}
uncertainty range:
[3.59e-12;4.37e-12]
ln(rate)=-26.25
reference shift: 0.0 minutes

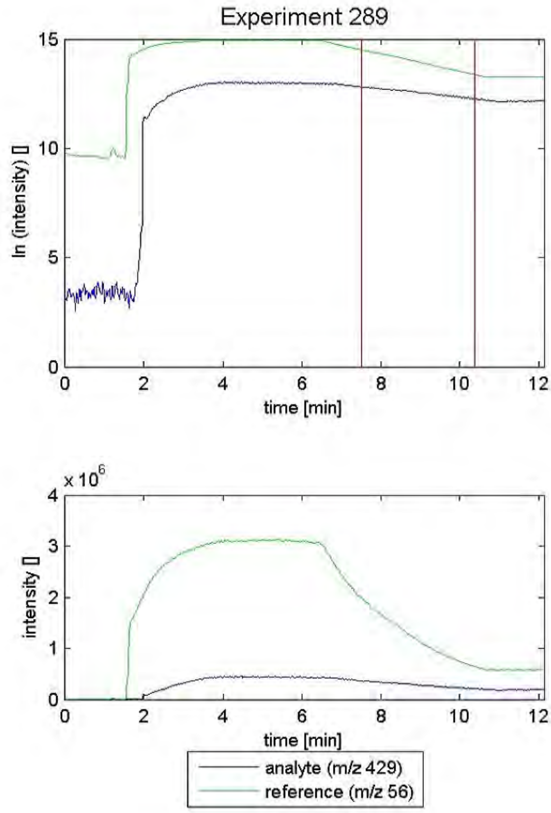

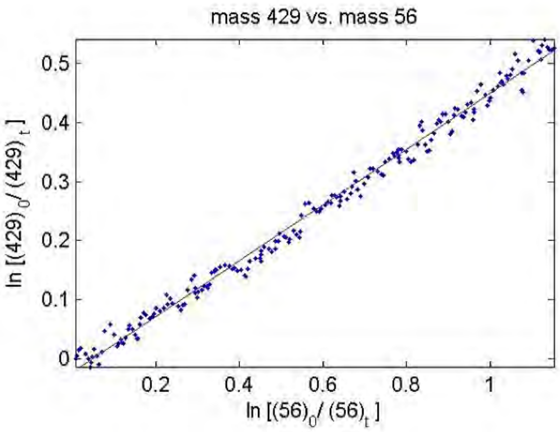

y= -0.023 + 0.471 * x
r= 0.995 n= 201
oven temperature: 50°C
analyte rate constant:
3.61e-12 cm^3 $molec^{-3}$ sec^{-1}
uncertainty range:
[3.26e-12;4.00e-12]
ln(rate)=-26.35
reference shift: 0.0 minutes

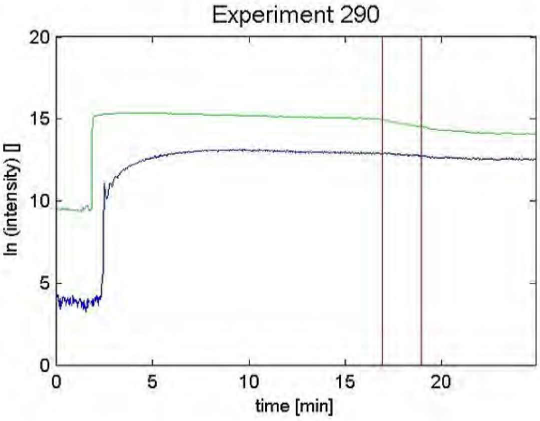

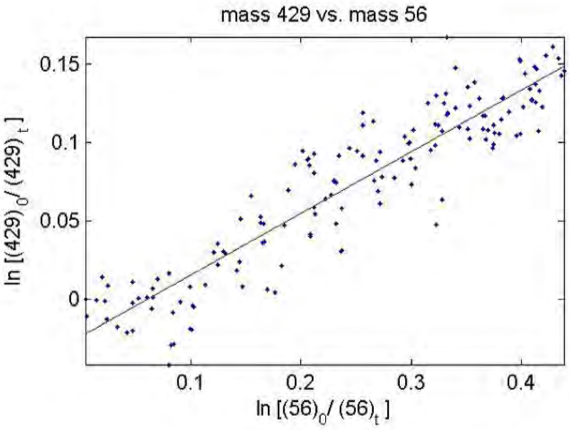

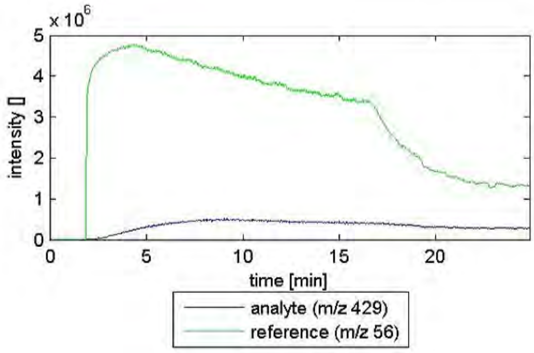

y= -0.024 + 0.393 * x
r= 0.924 n= 151
oven temperature: 50°C
analyte rate constant:
3.01e-12 cm^3 molec^{-3} sec^{-1}
uncertainty range:
[2.72e-12;3.33e-12]
ln(rate)=-26.53
reference shift: 0.0 minutes

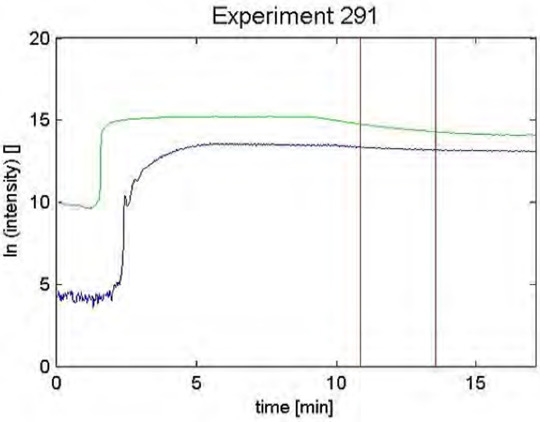

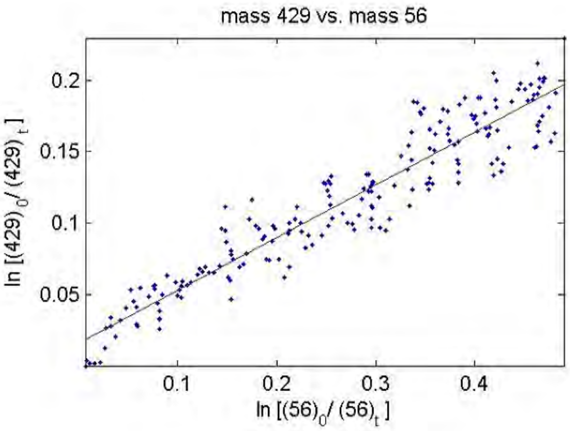

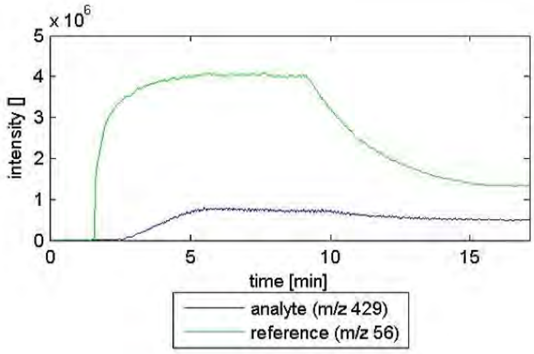

y= 0.016 + 0.369 * x
r= 0.947 n= 201
oven temperature: 50°C
analyte rate constant:
2.82e-12 cm^3 molec^{-3} sec^{-1}
uncertainty range:
[2.55e-12;3.13e-12]
ln(rate)=-26.59
reference shift: 0.0 minutes

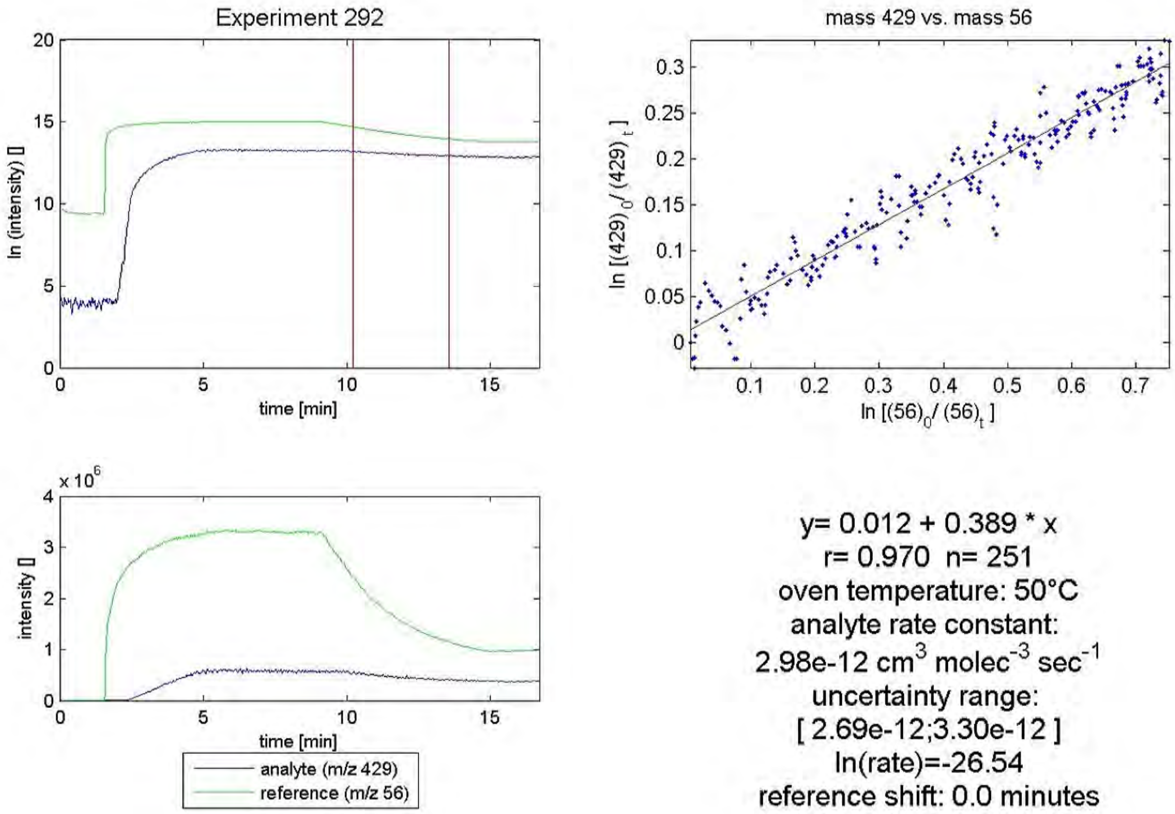

Experiment 292
ln (intensity) []
time [min]
x 10^6
intensity []
time [min]
analyte (m/z 429)
reference (m/z 56)
mass 429 vs. mass 56
ln [(429)_0 / (429)_t]
ln [(56)_0 / (56)_t]
y= 0.012 + 0.389 * x
r= 0.970 n= 251
oven temperature: 50°C
analyte rate constant:
2.98e-12 cm^3 molec^-3 sec^-1
uncertainty range:
[2.69e-12;3.30e-12]
ln(rate)=-26.54
reference shift: 0.0 minutes
